# Probabilistic modeling of cell cycle dynamics in response to cell cycle targeting chemotherapy drugs to guide treatment strategies

**DOI:** 10.1371/journal.pcbi.1013790

**Published:** 2025-12-16

**Authors:** Chenhui Ma, Evren Gurkan-Cavusoglu

**Affiliations:** Department of Electrical, Computer and Systems Engineering, Case Western Reserve University, Cleveland, Ohio, United States of America; University of Kiel Faculty of Medicine: Christian-Albrechts-Universitat zu Kiel Medizinische Fakultat, GERMANY

## Abstract

Understanding how chemotherapy perturbs cell cycle dynamics is critical for advancing cancer treatment. We develop a probabilistic, multi-generational framework based on a Bellman–Harris branching process to quantify treatment-induced shifts in tumor cell dynamics. The model incorporates key drug-responsive behaviors, including checkpoint activation, apoptosis, and checkpoint adaptation that propagates inherited DNA damage, enabling the characterization of heterogeneous survival outcomes after treatment. Biological parameters map directly onto DNA repair fidelity and cell-fate decisions, providing mechanistic insights beyond what is accessible from experiments alone. Dose-dependent extensions further allow exploration of treatment-induced perturbations. Model parameters were calibrated to empirical cell cycle measurements using the robust adaptive Metropolis algorithm. Global sensitivity analysis shows that scale parameters governing unfaithful DNA repair under G2/M- and S phase–specific agents exert major influence on model predictions, particularly at later time points. Across three chemotherapies, the framework reveals consistent dose-dependent alterations in cell cycle dynamics, with higher doses driving pronounced disruptions. Together, these results demonstrate how model-informed analyses can provide quantitative insight into treatment-induced cell cycle perturbations and support the refinement of therapeutic strategies.

## Author summary

Our work explores how chemotherapy impacts the complex life cycle of cancer cells to improve treatment strategies. Using a mathematical model, we simulated how drugs disrupt the cell cycle, a process critical for tumor growth. We focus on three widely used chemotherapeutic agents—paclitaxel, docetaxel, and gemcitabine—that affect different stages of the cell cycle, such as DNA replication and cell division. These drugs can block tumor growth, cause cell death, or even leave a lasting “memory” of damage in a cell’s offsprings, influencing their future behavior. By integrating experimental data and computational modeling, our approach provides insights into how cancer cells respond to treatment and exhibit heterogeneous survival outcomes. This framework offers a tool for exploring potential ways to optimize therapies, such as combining drugs or adjusting doses. By bridging biology and computation, our work aims to contribute to a better understanding of treatment effects and inform future research on improving cancer therapies.

## Introduction

Cell cycle progression is tightly regulated in normal tissues but becomes dysregulated in cancer, leading to uncontrolled cell proliferation. The cell cycle comprises distinct phases (G1, S, G2, and M). Each is critical for the accurate duplication and division of genetic material. Cancer treatments exploit these phases to effectively affect and kill tumor cells [1]. For instance, microtubule inhibitors such as docetaxel and paclitaxel interfere with mitotic spindle formation during the M phase, leading to mitotic arrest and cell death. Antimetabolites like gemcitabine block DNA replication in the S phase, thereby inhibiting cell proliferation. Additionally, defects in DNA damage checkpoints make cancer cells particularly susceptible to DNA-damaging agents such as platinum-based drugs that induce apoptosis [2]. Targeted therapies, such as CDK4/6 inhibitors, block the cell cycle transition, enhancing the effectiveness of chemotherapy by preventing efficient DNA repair and promoting cell death. Understanding the cell cycle’s role in cancer treatment is crucial, as it directly influences the radiation sensitivity of cells and the effectiveness of chemotherapeutic drugs. Specifically, the phase of the cell cycle determines a cell’s relative radiosensitivity; for example, cells are most radiosensitive in the G2/M phase [3]. Combining chemotherapeutics that affect different cell cycle phases can enhance antitumor effects, exploiting cellular vulnerabilities. Both radiation therapy and chemotherapeutic drugs can synchronize the cell cycle and arrest cells where repair mechanisms are activated [4, 5]. However, the effects of these drugs are influenced by factors like intra-tumor heterogeneity, cell plasticity, and tumor microenvironmental conditions. The interplay of these factors adds layers of complexity to the cell population dynamics, making it challenging to rely solely on experimental observations for a comprehensive understanding of therapeutic control.

Mathematical models of treatment-induced cell-cycle perturbations range from stochastic frameworks to population-balance formalisms, integrating phenomena such as DNA damage repair, checkpoint activation, and phase-specific progression to capture cellular responses to therapy [6]. Some models conceptualize the cell cycle as a series of compartments and utilize ordinary differential equations (ODEs) [7–11], integro-differential equations (IDEs) [12–14], and partial differential equations (PDEs) [15–18] to describe longitudinal dynamics of heterogeneous cell populations under treatment. While the population balance models are useful for studying population-level phenomena and are amenable to mathematical analysis, they may not fully capture the inherent stochasticity in the cell cycle processes and cell-to-cell variability in response to external stimuli. To address these limitations, probabilistic models, such as multi-stage models [19, 20], multi-generational models [21], and agent-based models [22–24], have been employed to address the inter-cell variability. One key probabilistic approach is the branching process model, which has been widely used to study the evolution of biological populations. In branching processes, cell division and cell death are treated as independent random events that dictate the fate of individual cells [25–27]. This framework enables the derivation of mean and variance formulas for cell numbers in different division classes, categories of cells grouped by the number of times they have divided, based on the distribution of cell cycle length [28]. Branching processes have been particularly useful in modeling the dynamics of cell populations under treatment. For example, Hyrien et al. [29] applied a multi-type age-dependent branching process to model the temporal development of precursor and terminally differentiated cells exposed to sublethal doses of carmustine (BCNU), a chemotherapeutic agent used to treat gliomas and non-Hodgkin’s lymphoma. In another study [30], a multi-type branching process was used to model the temporal development of cell populations subject to division and death during CFSE-labeling experiments. The theoretical underpinnings for the multi-type branching process are further explored in works by Norden et al. [31], and Li et al. [32].

We develop a probabilistic framework based on a multi-type Bellman–Harris branching process to quantify how phase-specific chemotherapies alter cancer cell population dynamics. These treatments affect different cell cycle phases. In particular, we examined paclitaxel and docetaxel, which disrupt mitosis, and gemcitabine, which inhibits DNA replication during the S phase. Our model is designed to track G2/M and S-specific lesions, checkpoint activation, apoptosis, and checkpoint adaptation in which cells progress despite unrepaired double-strand breaks (DSBs) [33–35]. Drawing on insights from Krenning et al.’s review [36] and experimental studies [37–39], we incorporated the intergenerational transmission of DNA damage, such that cell cycle status and repair history of mother cells influence the fate of daughter cells. The simulation workflow begins with an untreated Bellman-Harris branching process to establish baseline distributions of cell cycle phases. These distributions serve as initial conditions for simulating treatment-induced alterations in cell fate and proliferation dynamics. Drug-specific rules are then applied to both cells that are actively cycling at the onset of treatment and to newly generated cells, enabling us to capture both immediate and lineage-dependent responses to chemotherapy.

To capture the intergenerational effects of unresolved DNA damage, we incorporated a continuous time random walk (CTRW) model into our framework. CTRW, typically used in network diffusion contexts, is governed by a generalized integro-differential master equation for non-Poisson random walks on temporal networks [40, 41]. In our adaptation, the cell cycle is represented as a graph, with nodes corresponding to cell cycle phases and edges denoting transitions between them. This formulation enables us to account for how persistent DNA damage extends phase durations and alters population-level dynamics. By incorporating both the distribution and timing of cell cycle transitions, the CTRW framework captures both immediate treatment effects and long-term consequences across cell generations.

In our framework, heterogeneity in tumor response is captured as a consequence of differences in repair mechanisms, modeled as probabilistic cell-fate transitions during treatment. For example, when exposed to drugs affecting the G2/M phase, a cell may: (1) Evade checkpoint activation, continuing division despite damage associated with loss of cell cycle control [42]; (2) Undergo high-fidelity (faithful) repair, repairing damage and resuming normal cycling [43]; (3) Undergo low-fidelity (unfaithful) repair but resume division despite persistent DNA lesions, retaining residual damage [37–39, 44]; (4) Experience mitotic slippage, exiting mitosis without proper division [45, 46]. By modeling these transitions probabilistically, our framework captures how the stochastic nature of cell cycle progression and DNA damage repair contributes to heterogeneous survival outcomes following treatment, without any underlying geno- or phenotypic differences between cells prior to treatment. Even though such survivors may have the same drug sensitivity as the ancestral cells and could be eliminated with further treatment, they provide a pool of cells to re-populate the tumor. This is particularly true for those subpopulations that do not complete faithful repair and accumulate small or large-scale genetic aberrations, which can fuel the acquisition of resistance mutations.

This paper is organized as follows. We first describe the methodology used to construct the model without treatment. The treatment model is then adapted to evaluate the effects of three chemotherapeutic agents—docetaxel, paclitaxel, and gemcitabine. Docetaxel and paclitaxel primarily act on the G2/M phase, while gemcitabine affects the S phase. Model parameters are calibrated with *in vitro* flow cytometry data from two cancer cell lines: non-small cell lung cancer (NSCLC) and gastric cancer. These experiments, reported in [47, 48], inform both the baseline and treatment models. Parameter estimation is performed using the Robust Adaptive Metropolis (RAM) algorithm, with global sensitivity analysis applied to identify parameters that significantly influence model behavior. Dose-dependent parameters are introduced to investigate how varying drug concentrations affect treatment-induced cell cycle perturbations. Finally, we simulate cell cycle kinetics under combination therapy. The results of these simulations aim to inform dose-escalation trial design and support the development of more effective, personalized cancer treatment strategies. Table 1 summarizes the notations.

**Table 1:** Summary of the notations used in the model derivation throughout the paper.

Notation	Description
*T*	The sum of the duration of individual cell cycle phases
*G*(*T*)	The cumulative distribution function (cdf) of random time *T*
$N_{0}^{i}$	The count of the ancestor cells in the *i*h phase at simulation time zero
*τ*	The random variable for completion time of each phase in the cell cycle
$\psi_{i}(\tau )$	The probability density function (pdf) of the completion time *τ* for transitioning from phase *i* to phase *i* + 1
${\hat{\psi }}_{i}(s)$	The Laplace transform of $\psi_{i}(\tau )$
$g_{i}(s,t)$	The multivariate probability generating function (pgf) of the number of cells of all phases present in the process
initiated by an individual ancestor starting from the *i*th phase	
F(s,t)	The pgf of the entire cell cycle process
${𝐌}_{ij}(t)$	The expected number of progeny of type *j* produced by a cell of type *i*
*n*_*gen*_	The number of generations
m	Transition matrix
G(t)	A diagonal matrix of the cdf of time each cell spends in different states
E(·)	The expected value
$\Phi_{j}(t)$	The total number of cells in *j*th phase
*γ*	Growth rate of cell population
*p*_*i*_	The asymptotic percentage of cells in *i*th phase, i=G1,S,G2/M
*T*_*SSD*_	Time of treatment
*E*_*G*2/*M*_	Dose-dependent effect function describing inhibition of phase progression by G2/M phase–specific drug on phase progression
*E*_*d*,*G*2/*M*_	Dose-dependent probability of death following G2/M phase–specific drug
*E*_*S*_	Dose-dependent effect function describing inhibition of phase progression by S phase–specific drug on phase progression
*E*_*d*,*S*_	Dose-dependent probability of death following S phase–specific drug

## Methods

In developing our models for simulating tumor cell populations, both in the presence and absence of treatment, we based our approach on the Bellman-Harris branching process model [27]. This stochastic framework simulates the dynamics of proliferating cell populations by tracking their progression across generations. In a branching formulation, the modeling begins with a single ancestor cell, which divides after a specified time, resulting in a random number of offspring. Each of these new cells then repeats the cycle, continuing the process through generations. The Bellman-Harris process extends the classical branching models by allowing arbitrary lifetime (inter-division) distributions, thus accommodating realistic variability in cell cycle timing. Population evolution within this framework is described using renewal theory, which relates the number of cell divisions occurring over time to the distribution of intervals between successive division events [49]. In our case, interval distribution corresponds to the distribution of cell cycle division times, obtained as the convolution of individual phase duration distributions.

The model is built upon several assumptions. These include uniform drug delivery across the cell population, consistent levels of nutrients and oxygen, and the exclusion of nonlinear proliferation effects like contact inhibition, which is consistent with the characteristics of the *in vitro* data used in this study. Second, we have chosen not to explicitly include a separate G0 phase as a distinct state. Instead, cellular stalling typically attributed to G0 is represented through stochastic transitions from G1 to S. Finally, untreated cancer cells are assumed to progress through the cell cycle phases sequentially without spontaneous death events. This assumption is commonly seen in the study of cancer cell kinetics, particularly during the early stages of tumor growth [50, 51].

In this section, we first introduce a model describing progression through the cell cycle, and then extend it to incorporate the effects of chemotherapies that affect cells in the G2/M and S phases, acting on both the actively cycling cells at the time of treatment and those generated afterward.

### Branching process of dividing cells without treatment

In this section, we detail how we model the temporal evolution of both the percentage of cells within each cell cycle phase without treatment and the overall growth dynamics of the cellular population. As the Bellman-Harris model allows us to track cells in different cell cycle phases over time and derive closed-form expressions for them at any given time, we also explore the asymptotic behavior of these cell populations when growth reaches a balanced state, and the distribution of cells across the cell cycle becomes steady. Monitoring this asymptotic phase is important because it reflects the exponential growth stage of a tumor, which is typically the point at which treatments begin in *in vitro* experiments.

For more information about the derivations and methodologies, readers are referred to the ‘Branching process of dividing cells without treatment’ in the Supporting Information S1 File. In the main text, we focus on presenting the key steps and essential components of the model formulation to provide a concise overview.

Following the Bellman-Harris process, we began with an initial population of *N*_0_ ancestor cells at time *t* = 0. Fig A in Supplementary Information S1 File shows the illustration of the branching process of the cell cycle. Ancestor cells could theoretically start in any cell cycle phase. However, for model calibration, we assumed they are synchronized in the G1 phase at time 0 and run the simulation until balanced growth is reached. This assumption represents a specific scenario within the broader framework of a multi-type branching process. Nonetheless, to ensure the model’s generality, we also incorporated the possibility of ancestor cells starting in the S phase and G2/M phase in our calculations. Reflecting the cell cycle’s biology, each G1 and S phase cell progresses as a single cell to the next phase. After completing all cell cycle phases with a random time *T* with the cumulative distribution function (cdf) *G*(*T*), each G2/M phase cell produces two daughter cells simultaneously upon their mitosis. Each daughter cell then begins its cell cycle at *T* and lives for a random time with cdf *G*(*T*) before producing its progenies and initiating its branching process independently from its mother cells and sister cells. In the model derivations, $N_{0}^{i},i=1,2,3$ (1=G1,2=S,3=G2/M), represents the count of ancestor cells in the *i*th phase at time 0. The phase durations of each cell are independent and identically distributed.

The completion time of each phase in the cell cycle, denoted as *τ*, is treated as a random variable. A cell’s entry into the next phase depends on the time it has already spent in the current phase. The random variables $\tau_{1}$, $\tau_{2}$, and $\tau_{3}$ (representing the completion times of the G1, S, and G2/M phases, respectively) are modeled using the gamma distribution. The choice of the gamma distribution for modeling cell cycle phase time is well justified by both empirical measurements and *in silico* simulations, as shown in the study [52]. The function $\psi_{i}(\tau )$ in Eq (1) denotes the probability density function (pdf) of the phase duration *τ* for the transition from phase *i* to phase *i* + 1, with $\psi_{1}(\tau )$, $\psi_{2}(\tau )$, and $\psi_{3}(\tau )$ corresponding to the durations $\tau_{1}$ (G1), $\tau_{2}$ (S), and $\tau_{3}$ (G2/M), respectively. The corresponding cdfs are denoted by *G*_1_(*t*), *G*_2_(*t*), and *G*_3_(*t*).

$$\psi_{i}(\tau )=\frac{\beta_{i}^{\alpha_{i}}}{\Gamma (\alpha_{i})}\tau^{\alpha_{i}-1}e^{-\beta_{i}\tau }$$ (1)

Here, Γ represents the gamma function. $\beta_{i}$ and $\alpha_{i}$ are the rate and shape parameters respectively. We simplified the parameterization of the gamma distribution by setting a common rate parameter, $\beta_{i}=\beta_{0}$, for all phases (i=1,2,3). The utilization of a single rate parameter $\beta_{0}$ enables us to directly relate the mean cell cycle length and its variance to the sum of the shape parameters ($\sum_{i=1}^{3}\alpha_{i}$) and the common rate parameter $\beta_{0}$, through the relationships $\frac{\sum_{i=1}^{3}\alpha_{i}}{\beta 0}$ for the mean and $\frac{\sum_{i=1}^{3}\alpha_{i}}{\beta_{0}^{2}}$ for the variance. The values of $\alpha_{i}$ and $\beta_{0}$ were obtained by fitting the model to the literature data. The Laplace transform of $\psi_{i}(\tau )$ is given by

$${\hat{\psi }}_{i}(s)=\frac{\beta_{0}^{\alpha_{i}}}{(s+\beta_{0}{)}^{\alpha_{i}}}$$ (2)

where *s* is the complex frequency variable in the Laplace transform.

We later used the Laplace transform in the derivation of formulas for the steady state of cell cycle fractions given in Eq (8). The cell cycle length *T*, representing the time lapse between the entry into G1 until the exit out of G2/M, is a random variable in our model. Mathematically, it is defined as the sum of the duration of these individual phases: $T=\tau_{1}+\tau_{2}+\tau_{3}$. Its pdf is the convolution of the pdfs of $\tau_{1}$, $\tau_{2}$, and $\tau_{3}$ [53].

A useful tool for handling distributions of such random sums is the probability generating function (pgf), as it can be used to calculate the moments of the random variables. We obtained the multivariate generating function of the number of cells of all types (G1, S, G2/M phase cells) present in the process initiated by an individual ancestor starting from the *i*th phase, denoted as $g_{i}(s,t)$, conditioned on the waiting time *τ* of ancestor cell in the *i*th phase, as shown in Eq (3). Readers can refer to Appendix A “Multivariate Probability Generating Functions” in [27] and the appendix in [31] for background and related formulations relevant to Eq (3).

$$g_{i}(s,t)=s_{i}[1-G_{i}(t)]+\int_{0}^{t}f_{i}(g_{1}(s,t-\tau ),g_{2}(s,t-\tau ),g_{3}(s,t-\tau ))dG_{i}(\tau )$$ (3)

Here, $f_{i}(s)$ represents the multivariate progeny generating function of the *i*th type ancestor cells. s is a state vector of arbitrary variables, denoted as $s=[s_{1},s_{2},s_{3}]$, $\left|s_{1}\right|\leq 1,\ldots ,\left|s_{3}\right|\leq 1$. Specifically, the expression for *f*_*i*_(*s*) is $s_{1}^{2}$ for G2/M phase cells, *s*_2_ for G1 phase cells, and *s*_3_ for S phase cells.

As the individual branching processes are independently distributed, the pgf for the entire cell cycle process, denoted as F(s,t), is calculated as the product of the pgfs of all ancestor cells across the all three phases, i.e. $F(s,t)=\prod_{\;1.5pti=1}^{3}g_{i}(s,t{)}^{N_{0}^{i}}$. This gives us a comprehensive view of the cell cycle dynamics across all phases at any given time *t*.

To calculate the expected number of cells in a specific phase *j* at time *t* produced by the entire branching process, we substituted a specially constructed vector $s_{j}$ into F(s,t), where *s*_*j*_ corresponds to the phase of interest and all other components of s are set to 1. By differentiating F(s,t) with respect to *s*_*j*_ and then evaluating at *s*_*j*_ = 1, we can isolate the contribution of phase *j*, thereby determining the expected number of cells in that phase. The derivative $\frac{\partial F\left(s_{j},t\right)}{\partial s_{j}}$ at *s*_*j*_ = 1 then yields the expected number of cells in phase *j*, as shown in Eq (4).

$$E[\Phi_{j}(t)]={\frac{\partial F(s_{j},t)}{\partial s_{j}}|}_{s_{j}=1}={\sum_{i=1}^{3}N_{0}^{i}\frac{\partial g_{i}(s_{j},t)}{\partial s_{j}}|}_{s_{j}=1}$$ (4)

where $\Phi_{j}(t)$ is the total number of cells in *j* th phase.

Next, we solved for ${\frac{\partial g_{i}(s_{j},t)}{\partial s_{j}}|}_{s_{j}=1}$(denoted it as *M*_*ij*_(*t*)) and call it the expected number of cells in *j*th phase at time *t* in the process initiated by an ancestor cell in phase *i*. To capture the dynamics of cell division across generations, we extended this analysis by incorporating the generation factor *k* into the calculation of the expected number of cells. Specifically, we include *k* in *M*_*ij*_(*t*). The expected number of cells in phase *j* in generation *k*_2_, initiated by cells in phase *i* in generation *k*_1_, is denoted as $M_{i+3\left(k_{1}-1\right),j+3\left(k_{2}-1\right)}(t)$. For example, to determine how many S phase cells in the second generation are produced in the branching process initiated by the G1 cell in the first generation, it is necessary to calculate *M*_1,5_(*t*).

We then expressed the equation for *M*_*ij*_(*t*) in matrix form, denoted as 𝐌(t). Each element in 𝐌(t), denoted as ${𝐌}_{ij}(t)$, represents the expected number of progeny of type *j* produced by a cell of type *i*. At this stage, generation information is encoded in the indices *i* and *j*. The closed-form solution for 𝐌(t), shown in Eq (5), is derived using the Neumann series. The complete derivation of 𝐌(t), along with the algorithm used to compute it, is provided in Supporting Information S1 File.

$$𝐌(t)=\sum_{k=0}^{3n_{gen}}(Gm{)}^{*k}(t)*[𝐈-𝐆(t)]$$ (5)

where *n*_*gen*_ represents the number of generations. **m** represents the transition matrix. $G(t)=\mathrm{diag}(G_{1}(t),\ldots ,G_{K}(t)),K>1$ represents a diagonal matrix with each diagonal component representing the cdf of time each cell spends in different states.

Based on 𝐌(t), we were able to obtain the expected number of cells in the *j*th phase at any given time *t*. To match the experimental setup used in the literature data, it is assumed that the cell population starts in the G1 phase. The expected number of cells in the *j*th phase across all *n*_*gen*_ generations is given by Eq (6).

$$E\left[\Phi_{j}(t)\right]=N_{0}^{1}\sum_{k=1}^{n_{gen}}M_{1,3(k-1)+j}(t),j=1,2,3$$ (6)

We further used the fraction $E\left[\Phi_{j}(t)\right]/(E\left[\Phi_{1}(t)\right]+E\left[\Phi_{2}(t)\right]+E\left[\Phi_{3}(t)\right])$, the estimated cell fraction in the *j*th phase, to compare against the experimental data.

We analyzed the asymptotic behavior of the total cell population Φ(t). When t≫1, and in the absence of limiting factors such as space, nutrients, or cancer therapies, the tumor colony is expected to grow exponentially [54]. During this phase of exponential growth, the distribution of cells across the different phases of the cell cycle stabilizes, reaching stationary fractions. As a result, the expected number of cells for t≫1 can be described using Eq (7).

E[Φ(t)]~θexp(γt) as t≫1 (7)

where γ represents the mean growth rate, while *θ* is a scaling factor that accounts for the initial conditions of the system.

A key component of this study involves estimating the equilibrium fractions of cells in different cell cycle phases. Cowan established a relationship between the distribution of phase durations and the overall cell cycle time (*T*), showing that the fractional occupancy of each phase can be approximated from the distribution of its duration [55]. In the same work, Cowan derived analytical expressions for the probabilities that a cell resides in one of two hypothetical phases, which correspond to the asymptotic fractions of cells in each phase when the expected population size E[Φ(t)] becomes large (t→∞). Here, we extended the two-phase analysis to three phases. Eq (8) provides the numerical solution for the asymptotic fractions of cells in the G1, S, and G2/M phases.

$$p_{G1}=\frac{m\left[1-{\hat{\psi }}_{1}(\gamma )\right]}{m-1}=2\left[1-(\frac{\beta_{0}}{\gamma +\beta_{0}}{)}^{\alpha_{1}}\right]\;p_{S}=\frac{m\left[{\hat{\psi }}_{1}(\gamma )-{\hat{\psi }}_{1}(\gamma ){\hat{\psi }}_{2}(\lambda )\right]}{m-1}=2\left[(\frac{\beta_{0}}{\gamma +\beta_{0}}{)}^{\alpha_{1}}-(\frac{\beta_{0}}{\gamma +\beta_{0}}{)}^{\left(\alpha_{1}+\alpha_{2}\right)}\right]\;p_{G2/M}=\frac{m\left[{\hat{\psi }}_{1}(\gamma ){\hat{\psi }}_{2}(\gamma )-{\hat{\psi }}_{1}(\gamma ){\hat{\psi }}_{2}(\gamma ){\hat{\psi }}_{3}(\gamma )\right]}{m-1}=2\left[(\frac{\beta_{0}}{\gamma +\beta_{0}}{)}^{\left(\alpha_{1}+\alpha_{2}\right)}-(\frac{\beta_{0}}{\gamma +\beta_{0}}{)}^{\left(\alpha_{1}+\alpha_{2}+\alpha_{3}\right)}\right]$$ (8)

where *m* denotes the mean of the number of daughter cells produced by each cell. In the case of cell division, *m* is fixed as 2. We also provided the approximate solutions of γ in Supporting Information S1 File. The derivation for Eq (8) can be found in Section ‘Derivation of steady state’ in Supporting Information S1 File.

### The asymptotic state is used as the initial condition for the model with treatment

Under normal conditions, cells are considered to be in a state of asynchronous balanced growth when the percentage of cells in each of the three phases of the cycle remains constant, and cells are growing exponentially. This state is experimentally observed as unperturbed exponential growth in an in vitro system, where cells are kept far from confluence. In our model, the state of asynchronous balanced growth is used as the initial condition for the model with treatment.

To determine when the numerical solution of the model is asymptotically approaching an equilibrium state, we followed the method suggested in [56] and measured the percentage of cells in the G1 phase at each time step. We compared this to the percentage of cells in the G1 phase five hours earlier. If the squared difference between these two values was less than 10e–4, we conclude that an exponential growth state had been reached and denote that time as *T*_*SSD*_ where *SSD* stands for steady state distribution.

The experimental data used to calibrate the model without treatment inform how the model is initialized. Because experimental data indicate that cells are already in balanced growth prior to drug exposure, we initialize the untreated model with 100% G1-phase cells and simulate Eq (6) until the system reaches a steady state. We then compared this simulated steady state with the experimentally observed one. Additionally, we computed the analytical solution in Eq (8) and compared it with the observed steady-state distribution. Incorporating both numerical and analytical comparisons increases the number of model outputs available for fitting, which helps improve the robustness and reliability of parameter estimation for the untreated model.

### Branching process model under drug exposure

The chemotherapeutic drugs examined in our study are cytotoxic agents that can either directly or indirectly induce various types of DNA lesions (refer to Table 2 for details of their mechanisms of action (MoA)). DNA damage repair pathways are activated based on these specific lesion types. Two primary pathways, homologous recombination (HR) and non-homologous end joining (NHEJ), are utilized when facing double-strand breaks (DSBs). NHEJ operates throughout the cell cycle, including G1, S, G2, and mitotic phases, by directly ligating broken DNA ends [57]. It is particularly dominant in the G1 phase. In contrast, HR is primarily effective during the S and G2 phases when a sister chromatid is available, utilizing it for accurate repair of double-strand breaks. Other pathways include mismatch repair (MMR), which corrects mismatched bases, and base excision repair (BER) for smaller base lesions. However, these pathways can be defective, especially in cancer cells [58]. Defects in homologous recombination and double-strand break repair can be found in multiple malignancies, including non-small cell lung cancer (NSCLC) [59], ovarian cancer [60], and breast cancer [61], making them more vulnerable to certain DNA-damaging agents. To model the cell fate decision, we incorporated DNA damage repair as treatment-triggered cell states and categorized it into two broad classes: faithful and unfaithful repair, as these are consistently observed across various tumor cell lines. The two repair states account for the treatment-induced cell cycle arrest, as the processes of cellular repair and arrest are often regulated by interconnected signaling pathways. Our framework captures heterogeneity in treatment response by considering varying degrees of repair fidelity [62, 63] and the possibility of escape from cell cycle arrest. Faithfully repaired cells are assumed to resume cycling and produce progeny without additional damage, thereby sustaining population growth under treatment. The unfaithfully repaired cells can propagate genomic abnormalities to their offspring, contributing to treatment-induced cytotoxicity. We grouped the apoptosis arising from the failure of DNA damage repair and non-apoptotic cell death linked with mitotic catastrophe [64] into the cell death state.

**Table 2:** Mechanism of action (MoA) of three chemotherapeutic agents considered in the study.

Drug	MoA
Paclitaxel	Paclitaxel affects the G2/M phase by stabilizing microtubules, thereby disrupting mitotic spindle function and inducing mitotic arrest. Prolonged arrest may lead to mitotic slippage, allowing cells to exit mitosis without proper division and become polyploid. This process is associated with increased DNA damage, partly due to reactive oxygen species (ROS), and is typically repaired through the NHEJ pathway. Paclitaxel-induced stress can also lead to single-strand DNA breaks and trigger apoptosis or cell cycle arrest [65–70].
Docetaxel	Both paclitaxel and docetaxel act as G2/M phase inhibitors by disrupting microtubule dynamics, inducing mitotic arrest, and promoting apoptosis. While they share similar mechanisms, docetaxel differs in pharmacokinetics, exhibiting faster cellular uptake, longer intracellular retention, and a longer plasma half-life compared to paclitaxel. These differences can affect their cytotoxic profiles and treatment efficacy [71–74].
Gemcitabine	Gemcitabine (dFdC) is a nucleoside analog primarily active in the S phase, where it inhibits DNA synthesis by incorporating into DNA and blocking replication. It is phosphorylated intracellularly to dFdCTP, its active form, and also inhibits ribonucleotide reductase (RNR), reducing deoxynucleotide pools. This dual mechanism halts DNA synthesis and triggers cell cycle arrest. Although its primary action occurs in the S phase, gemcitabine may also affect the G1 and G2/M phases at higher concentrations due to nucleotide depletion and replication stress [75–78].

We also account for the inheritance of unresolved DNA damage (“memory mechanisms”), whereby daughter cells of surviving mothers carry replication stress forward and consequently exhibit quiescence or arrest in the next G1 phase, or impaired S phase re-entry.

When modeling treatment effects, we start the simulation at time zero under control conditions until it reaches the exponential growth phase at *T*_*SSD*_. Upon reaching *T*_*SSD*_, we introduced the impact of the treatment by modifying the transition matrix **m** to include the probability of a cell being arrested in different repair states and the corresponding likelihood of cell death. Additionally, the diagonal distribution matrix **G** is adjusted to reflect the cdf of cells arresting in these varied repair states. Details on these adjustments are provided in the Supporting Information S1 File. To facilitate comparison with experimental data, we rescaled the treatment time to 0.

We further categorized the cell population into two groups based on their state at the time of treatment and tracked how drug exposure differed between cells that were already mid-cycle when treatment began and those generated afterward. These categories reflect the timing of the most recent mitotic event relative to $T_{SSD}$, and thus define when each cell first encounters the drug. Fig C in Supporting Information S1 File illustrates the two groups.

1. **Cells actively cycling at $T_{SSD}$**: In the model, these cells correspond to those for which $M_{1,i}(T_{SSD})>1$, *i* represents the absolute order phase since simulation time 0. These cells are somewhere in the G1, S, G2/M phases when the drug is introduced. For each one, we computed the residual time it will spend in its current phase after $T_{SSD}$ (see Supporting Information S1 File). Each such cell will initiate a new branching process with dynamics that depend on whether it is in a phase directly affected by the drug. Cells in non-affected phases continue with baseline kinetics until they transition into a drug-affected phase (for example, a cell in G1 at $T_{SSD}$ is initially unaffected by G2/M phase drugs but becomes affected upon entering G2/M phase). In contrast, cells already in an affected phase experience altered transition probabilities and fate choices, as detailed in Sections “Cell cycle dynamics following the G2/M phase drugs" and “Cell cycle dynamics under S phase drug". Dashed lines in Fig C upper panel of the Supporting Information S1 File represent cells that remain actively cycling at *T*_*SSD*_.
2. **Cells generated after** $T_{SSD}$: In the model, these cells correspond to those for which ${\hat{𝐌}}_{p,1,i}(0)$ = 0. Here, ${\hat{𝐌}}_{p,1,i}(t)$, defined in Eq (S1.36) of Supporting Information S1 File, represents the number of cells in state *i* at time *t* after treatment in the branching process initiated by a cycling cell in phase *p* at *T*_*SSD*_. These cells are generated by cohort (1) after treatment has begun. Although they are not immediately affected by the drug, two exceptions apply: (i) When they progress into a drug-affected phase, they are directly affected; and (ii) they carry unrepaired DNA damage inherited from their mother cell, which can influence checkpoint activation and progression dynamics. Cells generated after *T*_*SSD*_ are represented by colorful dot-dashed lines in the Supporting Information S1 File.

In tailoring our branching model for drug exposure, we developed two distinct versions of the model, one capturing the effect of the G2/M phase drug and the other for the S phase drug, to reflect the impacts of chemotherapeutic drugs affecting specific phases of the cell cycle. This strategic approach enables an exploration of representative scenarios of G2/M and S phase-affecting chemotherapies. Specifically, we have chosen paclitaxel (PTX) and docetaxel (DTX) as illustrative compounds for testing the model’s performance with drugs primarily affecting the G2/M phase, given their established clinical relevance in cancer treatment. The S phase drug considered in our model is gemcitabine (GEM). The description of the model parameters and measurement noise parameters is summarized in Table 3 for both with and without treatment scenarios.

**Table 3:** Description of the parameters in the cell cycle phase transition distributions without treatment and with treatment (Refer to text for detailed description).

Parameters	Description	Units
**Parameters in cell cycle phase transition distribution**		
$\alpha_{1}$	The shape parameter of distribution of completion time in G1 phase	a
$\alpha_{2}$	The shape parameter of the distribution of completion time in S phase	a
$\alpha_{3}$	The shape parameter of distribution of completion time in G2/M phase	a
$\beta_{0}$	The rate parameter of completion time distribution in each cell cycle phase	a
**Treatment specific parameters (G2/M phase drug)**		
*q*_1,*G*2/*M*_	The probability of a G2/M cell, under drug exposure, entering a state of unfaithful DNA repair instead of progressing to the next phase	a
*q*_2,*G*2/*M*_	The probability of a G2/M cell, under drug exposure, entering a state of faithful DNA repair instead of progressing to the next phase	a
*q*_3,*G*2/*M*_	The probability that a cell exposed to the drug will arrest in G2/M before slipping out of G2/M without undergoing normal cytokinesis	a
*q*_4,*G*2/*M*_	The probability that the daughter cells proceed to the S phase without residual damage after arrest in the G1 phase	
$m_{d,FR,G2/M}^{max}$	The probability of cells in the state of faithful DNA repair dying in G2/M when the drug is at the maximal effect	a
$m_{d,UR,G2/M}^{max}$	The probability of cells in the state of unfaithful DNA repair dying in G2/M when the drug is at the maximal effect	a
$m_{d,MS,G2/M}^{max}$	The probability of cells in the state of arrest before mitotic slippage dying when the drug is at the maximal effect	a
$m_{d,G1arrest}^{max}$	The probability of daughter cells with residual damage dying in G1 when drug is at the maximal effect	a
$m_{d,Sarrest}^{max}$	The probability of daughter cells with residual damage dying in the S phase when the drug is at the maximal effect	a
$\lambda_{d,G1}^{max}$	The scale parameter of *D*_*d*,*G*1_(*t*) at the drug’s maximal effect	a
$\lambda_{UR,G2/M}^{max}$	The scale parameter of $G_{UR}^{G2/M}(t)$ at the drug’s maximal effect	a
$\lambda_{FR,G2/M}^{max}$	The scale parameter of $G_{FR}^{G2/M}(t)$ at the drug’s maximal effect	a
$\lambda_{MS,G2/M}^{max}$	The scale parameter of $G_{MS}^{G2/M}(t)$ at the drug’s maximal effect	a
$\lambda_{G1arrest}^{max}$	The scale parameter of *D*_*G*1*arrest*_(*t*) at the drug’s maximal effect	a
$\lambda_{d,S}^{max}$	The scale parameter of *D*_*d*,*S*_(*t*) at the maximal effect	a
$\lambda_{Sarrest}^{max}$	The scale parameter of *D*_*Sarrest*_(*t*) at the maximal effect	a
*b*_*G*2/*M*_	The shape parameter shared by *D*_*G*1*arrest*_(*t*), *D*_*Sarrest*_(*t*), $G_{UR}^{G2/M}(t)$ , $G_{FR}^{G2/M}(t)$, $G_{MS}^{G2/M}(t)$	a
*b*_*d*,*G*2/*M*_	The shape parameter of *D*_*d*,*G*1_(*t*) and *D*_*d*,*S*_(*t*)	a
*n*_*G*2/*M*_	The shape parameter in the sigmoidal Hill type function	a
*EC*_50,*d*,*G*2/*M*_	Half-maximal effective concentration for the probability of cell death	nM
*EC*_50,*G*2/*M*_	Half maximal parameter	nM
*a*_*S*_	The scale parameter of S phase in the first generation following treatment	a
*b*_*S*_	The shape parameter of S phase in the first generation following treatment	a
**Treatment specific parameters (S phase drug)**		
*q*_1,*S*_	The probability of cells being arrested in G1 phase	a
*q*_2,*S*_	The probability that an S phase cell exposed to the drug will be prevented from moving into the next phase and instead will enter a state of unfaithful DNA repair	a
*q*_3,*S*_	The probability that an S phase cell exposed to the drug will be prevented from moving into the next phase and instead will enter a state of faithful DNA repair	a
$m_{d,FR,S}^{max}$	The probability of cells in the state of faithful DNA repair dying in the S phase when the drug is at the maximal effect	a
$m_{d,UR,S}^{max}$	The probability of cells in the state of unfaithful DNA repair dying in the S phase when the drug is at the maximal effect	a
$m_{d,G2/Marrest}^{max}$	The probability of cells with residual damage dying in G2/M phase when drug is at the maximal effect	a
$m_{d,G1block}^{max}$	The probability of cells being arrested at the G1/S border dying under the maximal drug effect	a
$\lambda_{d,G2/M}^{max}$	The scale parameter of *D*_*d*,*G*2/*M*_(*t*) at the maximal effect	a
$\lambda_{UR,S}^{max}$	The scale parameter of $G_{UR}^{S}(t)$ at the maximal effect	a
$\lambda_{FR,S}^{max}$	The scale parameter of $G_{FR}^{S}(t)$ at the maximal effect	a
$\lambda_{G2/Marrest}^{max}$	The scale parameter of *D*_*G*2/*Marrest*_(*t*) at the maximal effect	a
$\lambda_{G1block}^{max}$	The scale parameter of *G*_*G*1*block*_(*t*) at the maximal effect	a
*b*_*S*_	The shape parameter shared by *D*_*G*2/*Marrest*_(*t*), $G_{UR}^{S}(t)$, $G_{FR}^{S}(t)$, and *G*_*G*1*block*_(*t*)	a
*b*_*d*,*S*_	The shape parameter of *D*_*d*,*G*2/*M*_(*t*)	a
*n*_*S*_	The shape parameter in the sigmoidal Hill type function	a
*EC*_50,*d*,*S*_	Half-maximal effective concentration for the probability of cell death	nM
*EC*_50,*S*_	Half maximal parameter	nM
*a*_*G*2/*M*_	The scale parameter of G2/M phase in the first generation following treatment	a
*b*_*G*2/*M*_	The shape parameter of G2/M phase in the first generation following treatment	a
**Parameters**	**Description**	**Units**
**Measurement noise for calibrating the model without treatment**		
$\sigma_{1,untreated}^{2}$	Variance of measurement noise for cell distribution in the G1 phase	a
$\sigma_{2,untreated}^{2}$	Variance of measurement noise for cell distribution in the S phase	a
$\sigma_{3,untreated}^{2}$	Variance of measurement noise for cell distribution in the G2/M phase	a
**Measurement noise for calibrating the model with treatment**		
$\sigma_{1,treated}^{2}$	Variance of measurement noise for cell distribution in the G1 phase	a
$\sigma_{2,treated}^{2}$	Variance of measurement noise for cell distribution in the S phase	a
$\sigma_{3,treated}^{2}$	Variance of measurement noise for cell distribution in the G2/M phase	a

a:dimensionless

#### Cell cycle dynamics following the G2/M phase drugs.

**Fig 1:**
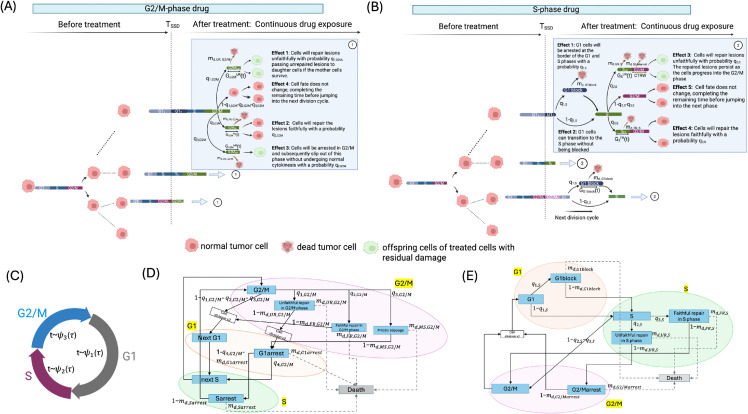
Schematic representation of cell state transitions under G2/M phase and S phase drug treatments. (A) Simulation of cell population dynamics under G2/M phase drug treatment, initiated at simulation time 0. Cells start in G1 and progress through the cycle until treatment is applied at time *T*_*SSD*_. After treatment, G2/M phase cells can transition into one of three states: (1) Unfaithful repair with probability *q*_1,*G*2/*M*_ and death rate *m*_*d*,*UR*,*G*2/*M*_, with duration modeled by the cdf $G_{G2/M}^{UR}(t)$; (2) Faithful repair with probability *q*_2,*G*2/*M*_ and death rate *m*_*d*,*FR*,*G*2/*M*_, cdf $G_{G2/M}^{FR}(t)$; (3) Mitotic slippage with probability *q*_3,*G*2/*M*_ and death rate *m*_*d*,*MS*,*G*2/*M*_, cdf $G_{G2/M}^{MS}(t)$. Created in BioRender. Ma, C. (2025) https://BioRender.com/nf7zhsn. (B) Simulation of cell population dynamics under S phase drug treatment. After treatment at *T*_*SSD*_, G1 cells may be arrested at the G1/S border with probability *q*_1,*S*_, with arrest time modeled by cdf *G*_*G*1*block*_(*t*). Cells that progress to S phase may: (1) Undergo unfaithful repair with probability *q*_2,*S*_ and death rate *m*_*d*,*UR*,*S*_, cdf $G_{S}^{UR}(t)$; (2) Undergo faithful repair with probability *q*_3,*S*_ and death rate *m*_*d*,*FR*,*S*_, cdf $G_{S}^{FR}(t)$. Both models start with 100% G1 cells. Treatment is applied when the cell population is in the exponential growth stage (at the *T*_*SSD*_). *P*_1_ and *P*_2_ represent the duration of phase P∈{G1,S,G2/M} before drug exposure and after drug exposure. Created in BioRender. Ma, C. (2025) https://BioRender.com/0zr6sxj. (C) Baseline model of untreated cell cycle dynamics, where cells sequentially transition through G1, S, and G2/M. Phase durations follow Gamma distributions $\psi_{1}(\tau ),\psi_{2}(\tau ),\psi_{3}(\tau )$. The population-level dynamics are governed by a Bellman-Harris branching process, which allows for the analytical derivation of steady-state phase distributions under exponential growth. Created in BioRender. Ma, C. (2025) https://BioRender.com/hhyr81h. (D) State transition diagram for G2/M phase drug. Transition rates between states are also elements of the matrix **m**; (E) State transition diagram for S phase drug. Transition rates between states are elements of the matrix **m**.

In this section, we examine the impact of the chemotherapeutic agents PTX and DTX on the cell cycle dynamics of G2/M phase cells. Both drugs are known to disrupt normal cell cycle progression by stabilizing microtubules, thereby inhibiting mitosis and triggering cell cycle arrest in the G2/M phase. The possible transitions for the impacted G2/M cells under these drugs are shown in Fig 1A and 1D. The G1 cells in the cycle remain unaffected by these treatments. After the G1 phase residual time is completed, cells advance to the S phase with recalibrated dynamics altered by the treatment exposure. The fates of both the newly produced G2/M cells after exposure and cycling G2/M cells at *T*_*SSD*_ will be changed, resulting in four possible outcomes shown as follows.

1. **Effect 1: Unfaithful Repair (UR)**.
  The G2/M phase cells that are affected by the drug will repair the lesions unfaithfully with a probability *q*_1,*G*2/*M*_. Lesions that remain unrepaired in mother cells are passed down to daughter cells if the mother cells survive the initial damage. This unsuccessful repair state is denoted as UR (Unfaithful Repair). In this state, the G2/M cells face a specific death rate, represented by *m*_*d*,*UR*,*G*2/*M*_ (see Eq (10)). This rate quantifies the likelihood of cell death attributable to the persistence of DNA damage that compromises genetic integrity. The cdf of time elapsed in the unfaithful repair state is denoted as $G_{G2/M}^{UR}(t)$, which follows a Weibull distribution, describing the distribution of time that cells persist in this state before transitioning to the next phase or undergoing cell death.
2. **Effect 2: Faithful Repair (FR)**.
  The G2/M phase cells that are affected by the drug will repair the lesions faithfully with a probability denoted by *q*_2,*G*2/*M*_. When repair is executed faithfully, it ensures that no unrepaired damage is transferred to the daughter cells. This successful repair state is denoted as FR (Faithful Repair). The death rate of G2/M cells in the FR state is expressed by *m*_*d*,*FR*,*G*2/*M*_ (see Eq (10)). Although these cells have repaired their DNA damage effectively, the death rate *m*_*d*,*FR*,*G*2/*M*_ still accounts for the residual risk of apoptosis or other forms of cell death that might occur due to factors such as residual stress from the damage and repair processes. The cdf of time elapsed in the faithful repair state is denoted as $G_{G2/M}^{FR}(t)$, which models the Weibull distribution of time that cells spend in this repair state before transitioning to the next phase or undergoing cell death.
3. **Effect 3: Mitotic Slippage (MS)**.
  The G2/M phase cells that are affected by the drug will be arrested in G2/M due to spindle assembly checkpoint activation and may undergo mitotic slippage (MS), a process in which cells exit mitosis without proper chromosome segregation or cytokinesis. In other words, they “slip out” of mitosis without dividing, often becoming tetraploid or polyploid. This occurs with a probability denoted by *q*_3,*G*2/*M*_. After slippage, the cells may progress into the G1 phase, where they continue their repair processes, although their viability is compromised. Alternatively, these cells might undergo cell death or enter a state of senescence. The rate at which G2/M cells in the mitotic slippage state die is denoted by *m*_*d*,*MS*,*G*2/*M*_ (see Eq (10)). This rate quantifies the likelihood of cells leaving the active cell cycle through cell death, reflecting the risks associated with abnormal cell division and unresolved damage. The cdf of time elapsed in the mitotic slippage state is denoted as $G_{G2/M}^{MS}(t)$, which follows a Weibull distribution.
4. **Effect 4: Normal G2/M → G1 Transition**.
  The cell fate does not change with a probability denoted by $1-q_{1,G2/M}-q_{2,G2/M}-q_{3,G2/M}$. This type of G2/M cells will complete the remaining time before entering the subsequent cell cycle.

The newborn G1 cells and S cells that appear after *T*_*SSD*_ exhibit different dynamics compared to their mother cells, as their behavior is affected by the mother cells’ state of repair. Conventional models of cell cycle dynamics, deterministic or simple stochastic formulations, generally neglect the complexities introduced by such residual damage. To capture these effects, we adopted the continuous-time random walk (CTRW) framework of Petit et al. [79]. This model is well-suited to describe G1 and S phase cells inheriting residual damage from their mother cells, either due to their mother cells’ incomplete repair or mitotic slippage. In the CTRW formulation, cell transitions within the daughter cells with residual damage are modeled as a form of a random walk on an acyclic graph, where the duration of the cell cycle phases for daughter cells is determined by a competition between “Downtime" and “Uptime" periods, alongside the normal cell cycle time. Here, “Downtime" refers to periods when repair processes halt cell cycle progression, including checkpoint activation and DNA damage responses. “Uptime" represents active progression through the cycle following the resolution of inherited drug-induced damage.

Newborn G1 cells inheriting damage from UR and MS cells were designed to have a mortality rate, denoted as *m*_*d*,*G*1*arrest*_, detailed in Eq (10). We refer to this state as G1arrest. Such damaged G1 cells can either progress to the S phase with DNA damage, a state we call Sarrest, where the damage from the G1 phase continues to be repaired, or they may enter a normal S phase with dynamics identical to the control case. The probabilities of transitioning to the damaged S phase and progressing to a normal S phase are 1 − *m*_*d*,*G*1*arrest*_ − *q*_4,*G*2/*M*_ and *q*_4,*G*2/*M*_, respectively. Notably, we do not distinguish between faithful and unfaithful repair in the newly produced damaged S phase cells post *T*_*SSD*_, as they will be affected again by the G2/M phase drug once they progress to the G2/M phase. Within the CTRW model, we use *D*_*G*1*arrest*_(*t*) and *D*_*Sarrest*_(*t*) to represent the downtime distributions at the edge to the next phase, reflecting the duration of the repair process in damaged G1 and S phase cells. Meanwhile, *D*_*d*,*G*1_(*t*) and *D*_*d*,*S*_(*t*) quantify the downtime distribution at the edge leading to cell death, signifying the apoptotic process in the G1 and S phase cells. For a detailed explanation of the CTRW model formulation, refer to Supplementary Information S1 File.

The shape parameters of the aforementioned downtime distributions *D*_*G*1*arrest*_(*t*), *D*_*Sarrest*_(*t*), *D*_*d*,*G*1_(*t*), *D*_*d*,*S*_, $G_{G2/M}^{UR}(t)$ , $G_{G2/M}^{FR}(t)$, $G_{G2/M}^{MS}(t)$ are kept the same and are denoted as *b*_*G*2/*M*_. The scale parameters of these distributions are denoted as $\lambda_{G1arrest}$, $\lambda_{Sarrest}$, $\lambda_{d,G1}$, $\lambda_{d,S}$, $\lambda_{UR,G2/M}$, $\lambda_{FR,G2/M}$, $\lambda_{MS,G2/M}$. These parameters are assumed to be dose-dependent using Hill equations and mathematically expressed as Eq (9), similar to our previous work in [80].

$$E_{G2/M}=\left(\frac{{\left({𝒟}_{G2/M}/EC_{50,G2/M}\right)}^{n_{G2/M}}}{1+{\left({𝒟}_{G2/M}/EC_{50,G2/M}\right)}^{n_{G2/M}}}\right)\;\lambda_{UR,G2/M}=\lambda_{UR,G2/M}^{max}E_{G2/M}\;\lambda_{FR,G2/M}=\lambda_{FR,G2/M}^{max}E_{G2/M}\;\lambda_{MS,G2/M}=\lambda_{MS,G2/M}^{max}E_{G2/M}\;\lambda_{G1arrest}=\lambda_{G1arrest}^{max}E_{G2/M}\;\lambda_{Sarrest}=\lambda_{Sarrest}^{max}E_{G2/M}\;\lambda_{d,G1}=\lambda_{d,G1}^{max}E_{G2/M}\;\lambda_{d,S}=\lambda_{d,S}^{max}E_{G2/M}\;$$ (9)

Here, *E*_*G*2/*M*_ represents the effect of the treatment on G2/M phase cells, modeled using the Hill equation, which describes dose-response relationships. ${𝒟}_{G2/M}$ denotes the dose of the drug affecting G2/M phase cells. *EC*_50,*G*2/*M*_ represents the half-maximal effective concentration. The Hill coefficient, *n*_*G*2/*M*_, indicates the steepness of the dose-response curve. The value of *E*_*G*2/*M*_ ranges from 0 (no effect) to 1 (maximal effect). This term scales the parameters of the downtime distributions, making them dose-dependent.

Eq (10) describes the dose-dependent probability that the cells in FR, UR, MS, G1arrest, and Sarrest states die following G2/M phase drug exposure.

$$E_{d,G2/M}=\left(\frac{{\left({𝒟}_{G2/M}/EC_{50,d,G2/M}\right)}^{n_{G2/M}}}{1+{\left({𝒟}_{G2/M}/EC_{50,d,G2/M}\right)}^{n_{G2/M}}}\right)\;m_{d,FR,G2/M}=m_{d,FR,G2/M}^{max}E_{d,G2/M}\;m_{d,UR,G2/M}=m_{d,UR,G2/M}^{max}E_{d,G2/M}\;m_{d,MS,G2/M}=m_{d,MS,G2/M}^{max}E_{d,G2/M}\;m_{d,G1arrest}=m_{d,G1arrest}^{max}E_{d,G2/M}\;m_{d,Sarrest}=m_{d,Sarrest}^{max}E_{d,G2/M}\;$$ (10)

where *EC*_50,*d*,*G*2/*M*_ signifies the half-maximal effective concentration for the probability of cell death. *n*_*G*2/*M*_ represents the Hill coefficient, which describes the steepness of the dose-response curve. The equations suggest the fraction response *m*_*d*,*FR*,*G*2/*M*_ is modeled as a sigmoidal curve, with $m_{d,FR,G2/M}^{\max}$ as the maximum response achievable. The response increases with increasing dose ${𝒟}_{G2/M}$ but saturates at high doses due to the sigmoidal nature of the curve. The Hill coefficient *n*_*G*2/*M*_ used in Eq (9) also controls the steepness of this curve. The other equations in Eq (10) follow a similar structure, representing fractional responses under different conditions or scenarios.

#### Cell cycle dynamics under S phase drug.

In this section, we explore the multifaceted impact of gemcitabine. Gemcitabine works by inhibiting DNA synthesis and affecting S phase cells. It also impacts the G1 phase of the cell cycle. Its effects include inducing variable, dose-dependent G1 blocks and causing delays at subsequent cell cycle checkpoints, particularly in the G2/M and G1 phases during cell cycle re-entry [81]. The transitions for the affected G1 and S cells under gemcitabine are illustrated in Fig 1B and E. The fate of cycling S phase cells, G1 phase cells, and new S cells after exposure at the time of treatment (*T*_*SSD*_) can result in five possible outcomes shown as follows.

1. **Effect 1: G1Block.**
  G1 cells will be arrested at the border of the G1 and S phases with a probability *q*_1,*S*_. This state is denoted as G1block. In this state, the death rate of G1 cells, represented by *m*_*d*,*G*1*block*_ (see Eq (12)), quantifies the likelihood of cell death while the cells are arrested at this transition point. The cdf of the time that cells spend when blocked in the G1 phase is denoted as *G*_*G*1*block*_(*t*), which models the Weibull distribution of the duration of arrest in G1block before cells either transition to the S phase or undergo apoptosis.
2. **Effect 2: Normal G1 → S Transition.**
  G1 cells can transition to the S phase without being blocked with a probability denoted by 1−*q*_1,*S*_.
3. **Effect 3: Unfaithful Repair (UR).**
  The S phase cells that are affected by the drug will repair the lesions unfaithfully with a probability denoted by *q*_2,*S*_. This state is referred to as UR (Unfaithful Repair). In this state, lesions that are not adequately repaired in the S phase persist as the cells progress into the G2/M phase. The death rate of S cells in this state, represented by *m*_*d*,*UR*,*S*_ (Eq (12)), quantifies the likelihood of cell death due to the persistence of these unrepaired lesions, which can compromise the cells’ genomic stability. The cdf of time elapsed in the unfaithful repair state is denoted as $G_{S}^{UR}(t)$, modeling the Weibull distribution of the duration that cells remain in this state before either advancing to the next cell cycle phase or undergoing cell death.
4. **Effect 4: Faithful Repair (FR).**
  The S phase cells that are affected by the drug will repair the lesions faithfully with a probability denoted by *q*_3,*S*_. This state is referred to as FR (Faithful Repair). In this state, all damage is successfully repaired. There will not be unrepaired damage taken to the next phase. The death rate for S cells in the FR state, represented by *m*_*d*,*FR*,*S*_ (see Eq (12)), quantifies the likelihood of cell death due to residual cellular stress or other cytotoxic effects of the drug. The cdf of time elapsed in the faithful repair state is denoted as $G_{S}^{FR}(t)$, modeling the Weibull distribution of the duration that cells remain in this state before either progressing in the cell cycle or undergoing cell death.
5. **Effect 5: Normal S → G2/M Transition.**
  The cell fate does not change with a probability of $1-q_{2,S}-q_{3,S}$. In this situation, the S phase cells will complete the remaining time before jumping into G2/M phase.

The dynamics of G2/M cells are influenced by residual damage carried forward from S phase cells that underwent unfaithful repair. As illustrated in Fig 1B, we employed the CTRW model for the G2/M cells receiving such damage. In the CTRW model, the downtime distribution associated with the transition to the next phase, representing the repair process, is denoted as *D*_*G*2/*Marrest*_(*t*). The downtime distribution on the edge to death, representing the apoptotic process, is denoted as *D*_*d*,*G*2/*M*_(*t*). These damaged G2/M cells are referred to as G2arrest. These damaged G2/M cells have a death rate denoted as *m*_*d*,*G*2/*Marrest*_ (Eq (12)). Damaged G2/M cells can have one of two fates. They might divide to produce new G1 cells. Alternatively, they might die in the G2/M phase. The respective probabilities for these transitions are $1\;-\;m_{d,G2/Marrest}$ and *m*_*d*,*G*2/*Marrest*_ (see Eq (12)).

The shape parameters of cdfs *D*_*G*2/*Marrest*_(*t*), *D*_*d*,*G*2/*M*_(*t*) and $G_{S}^{UR}(t)$, $G_{S}^{FR}(t)$, *G*_*G*1*block*_(*t*) are kept the same and are denoted as *b*_*S*_. The scale parameters of these cdfs are denoted as $\lambda_{G2/Marrest}$, $\lambda_{d,G2/M}$, $\lambda_{UR,S}$, $\lambda_{FR,S}$, and $\lambda_{G1block}$. They are assumed to be dose-dependent, and mathematically expressed as Eq (11).

$$E_{S}=\left(\frac{{\left({𝒟}_{S}/EC_{50,S}\right)}^{n_{S}}}{1+{\left({𝒟}_{S}/EC_{50,S}\right)}^{n_{S}}}\right)\;\lambda_{UR,S}=\lambda_{UR,S}^{max}E_{S}\;\lambda_{FR,S}=\lambda_{FR,S}^{max}E_{S}\;\lambda_{G2/Marrest}=\lambda_{G2/Marrest}^{max}E_{S}\;\lambda_{G1block}=\lambda_{G1block}^{max}E_{S}\;\lambda_{d,G2/M}=\lambda_{d,G2/M}^{max}E_{S}\;$$ (11)

where *E*_*S*_ represents the effect of the treatment on S phase cells, modeled using the Hill equation, which describes dose-response relationships. ${𝒟}_{S}$ denotes the dose of the drug affecting S phase cells. *EC*_50,*S*_ represents the half-maximal effective concentration. The Hill coefficient, *n*_*S*_, indicates the steepness of the dose-response curve. The value of *E*_*S*_ ranges from 0 (no effect) to 1 (maximal effect). This term scales the parameters of the downtime distributions, making them dose-dependent.

Eq (12) describes the dose-dependent probability of the cells that jump to the death state following the treatment of the S phase drug. ${𝒟}_{S}$ represents the dose of the S phase drug treatment.

$$E_{d,S}=\left(\frac{{\left({𝒟}_{S}/EC_{50,d,S}\right)}^{n_{S}}}{1+{\left({𝒟}_{S}/EC_{50,d,S}\right)}^{n_{S}}}\right)\;m_{d,FR,S}=m_{d,FR,S}^{max}E_{d,S}\;m_{d,UR,S}=m_{d,UR,S}^{max}E_{d,S}\;m_{d,G2/Marrest}=m_{d,G2/Marrest}^{max}E_{d,S}\;m_{d,G1block}=m_{d,G1block}^{max}E_{d,S}\;$$ (12)

where *EC*_50,*d*,*S*_ signifies the half-maximal effective concentration for the probability of cell death. *n*_*S*_ represents the Hill coefficient, which describes the steepness of the dose-response curve. The equations show the fraction response *m*_*d*,*FR*,*S*_ is modeled as a sigmoidal curve, with $m_{d,FR,S}^{\max}$ as the maximum response achievable. The response increases with increasing dose ${𝒟}_{S}$ but saturates at high doses due to the sigmoidal nature of the curve. The steepness of this curve is controlled by the Hill coefficient *n*_*S*_. The rest of the equations in Eq (12) follow a similar structure, representing fractional responses under different conditions or scenarios. Visualization of Eq (9), Eq (10), Eq (11), and Eq (12) can be found in Supplementary Information S2 Fig.

### Model calibration using adaptive metropolis algorithm

Markov Chain Monte Carlo (MCMC) is a computational technique used to estimate the distribution of parameters in complex models from observed data. Its goal is to generate samples that approximate the posterior distribution of these parameters, facilitating probabilistic inference and prediction. In this study, the posterior distribution is obtained by combining the likelihood of the observed data given the model parameters with the prior beliefs about these parameters. MCMC is then applied to infer the distributions of model parameters that are consistent with the observed data.

The model was implemented in MATLAB (version R2021a). Parameter sets are denoted as $\Theta_{i}\in {\mathbb{R}}^{m_{i}},i=1...M$, where *m*_*i*_ is the number of parameters in model *i* and *M* is the total number of data sets. The likelihood is formulated using the additive noise model that quantifies the difference between the observed values and model predictions. Specifically, for dataset $S_{i},i=1....M$, the likelihood function for estimating the parameter set $\Theta_{i}$ is calculated as Eq (13). In the untreated model, this likelihood Eq (13) incorporates the difference between the numerical solution of Eq (8), model simulations generated by Eq (6), and steady cell distribution percentages.

$${ℒ}_{i}(S_{i}|\Theta_{i},\sigma_{i})=\prod_{p=1}^{3}\frac{1}{(2\pi \sigma_{i,p}^{2}{)}^{n_{i,p}/2}}\exp\left(-\frac{1}{2\sigma_{i,p}^{2}}||f_{i,p}(\Theta_{i})-d_{\;1.5pti,p}|{|}^{2}\right)$$ (13)

where $d_{\;1.5pti,p}\in {\mathbb{R}}^{n_{i,p}},i=1...M$. $d_{\;1.5pti,p}$ represents the time series data of the percentage of *p*th phase cells at time *t* in data set *i*. cells in phase *p* at time *t* in dataset *i*. The corresponding model predictions are given by $f_{i,p}\left(\Theta_{i}\right)$, where $\Theta_{i}$ is the parameter set associated with dataset *i*. For each dataset, the discrepancy between the observed data at sampled time points, $d_{\;1.5pti,p}^{\;1.5ptj}$, $j=1,\ldots ,n_{i,p}$, and model predictions is represented by the measurement noise term, $\varepsilon_{i,p}$. *n*_*i*,*p*_ denotes the total number of time points in the *i*th dataset for the *p*th phase. We assumed that $\varepsilon_{i,p}$ are independent, normally distributed random variables with zero mean and the variance $\sigma_{i,p}^{2}$. Instead of keeping $\sigma_{i}$ known, we treated them as random variables whose marginal distributions can be learned from the data. All parameters are assumed independent random variables, so the joint posterior distribution of the parameter set $\Theta_{i},i=1...M$ is determined for each data set by Eq (14).

$$\pi_{i}(\Theta_{i}|S_{i})\propto {ℒ}_{i}(S_{i}|\Theta_{i},\sigma_{i})\pi (\sigma_{i})\pi (\Theta_{i})\;\propto {ℒ}_{i}(S_{i}|\Theta_{i},\sigma_{i})\prod_{p=1}^{3}\pi (\sigma_{i,p})\prod_{k=1}^{m_{i}}\pi (\theta_{ik})$$ (14)

where *m*_*i*_ represents the number of parameters that need to be estimated in $\Theta_{i}$. $\pi_{i}(\Theta_{i}|S_{i})$ represent the posterior distribution of $\Theta_{i}$. $\pi (\sigma_{i,p})$ and $\pi (\theta_{ik})$ represent the prior distributions of the parameter sets of $\sigma_{i,p}$ and $\theta_{ik}$. As in our previous work [80], we used the robust adaptive Metropolis algorithm (RAM) [82] for MCMC sampling. The uniform distribution is used as the prior distribution for all the parameters in the treatment models. This choice of non-informative priors is justified by the lack of prior knowledge about the specific parameter values. We used wide initial bounds with the RAM algorithm to enable broad exploration of parameter space, ensuring that convergence was achieved and that the priors covered all plausible parameter values.

## Results

### Cell cycle models for untreated cases

This study models cell populations using data from two studies. Table 4 overviews data sources for calibrating parameters in both untreated and treated models. Balcer-Kubiczek et al. [47] studied docetaxel’s effect on human gastric cancer cells’ low-dose radiation hypersensitivity, measuring cell survival and cycle distribution via flow cytometry. Kroep et al. [48] explored paclitaxel’s sequence-dependent effects on gemcitabine metabolism in non-small-cell lung cancer cell lines, assessing cell cycle progression and cytotoxicity with flow cytometry and biochemical assays.

**Table 4:** Data sources from the literature utilized for calibrating model parameters.

Model	Treatment	Dose Regimen	Cell Line	Ref
Model 1	Docetaxel	3 nM	AGS gastric cancer cells	[47]
Model 2	Paclitaxel	10 × IC-50 concentrations at 72h	H460 NSCLC cells	[48]
Model 3	Gemcitabine	10 × IC-50 concentrations at 72h	H460 NSCLC cells	[48]

**Fig 2:**
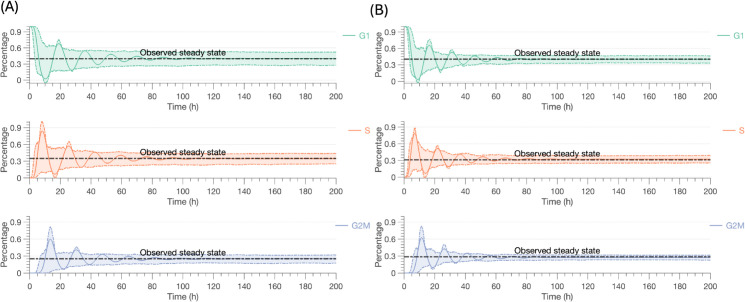
Simulated cell cycle trajectories over time for the baseline models. (A) Model 1; (B) Models 2 and 3. The solid lines represent the mean trajectory for each cell cycle phase: G1 (green), S (orange), and G2/M (blue). The shaded areas indicate the 50% credible intervals for the trajectories, reflecting the variability and uncertainty in the model predictions. The dashed horizontal lines mark the observed steady state percentages for each phase, demonstrating the model’s alignment with experimental data at equilibrium. The credible interval width reflects the spread of parameter values sampled from the posterior distribution, rather than from the inherent stochasticity of the branching process.

When developing the untreated model, we start with all the cells in the G1 phase and let the untreated model run until it reaches the steady state. The mean values of the estimated parameters with their credible intervals (CI) are reported in Table 5, and the corresponding posterior distributions are shown in the Supporting Information S2 File. Histograms of posterior distributions for shape parameters for cell cycle distributions $\alpha_{1}$ (G1), $\alpha_{2}$ (S), and $\alpha_{3}$ (G2/M) reveal unimodal shapes, indicating reasonable identifiability. Simulated cell cycle trajectories are shown in Fig 2, demonstrating stable convergence to the observed steady-state percentages across G1, S, and G2/M phases of the cell cycle. The alignment between the simulation and the observed equilibrium, shown by the trajectories settling into the dashed-line steady state, validates the model’s ability to reproduce long-term cell cycle dynamics. Uncertainty in model predictions is represented by the shaded regions showing the 50% credible intervals.

**Table 5:** Parameter estimates and their credible intervals for the three baseline models and corresponding models for the treatment cases.

	Value (50% CI)		Value (50% CI)	
**Parameters**	**Model 1**	**Model 2**	**Parameters**	**Model 3**
**Baseline model**			**Baseline model**	
$\alpha_{1}$	10.156(6.186,13.937)	9.215(7.174,11.155)	$\alpha_{1}$	9.215(7.174,11.155)
$\alpha_{2}$	11.068(7.243,14.021)	8.946(6.372,11.263)	$\alpha_{2}$	8.946(6.372,11.263)
$\alpha_{3}$	10.147(6.693,13.058)	10.017(8.112,12.070)	$\alpha_{3}$	10.017(8.112,12.070)
$\beta_{0}$	1.797(0.972,2.498)	1.824(1.040,2.466)	$\beta_{0}$	1.824(1.040,2.466)
$\sigma_{1,untreated}^{2}$	0.248(0.128,0.353)	0.157(0.047,0.233)	$\sigma_{1,untreated}^{2}$	0.157(0.047,0.233)
$\sigma_{2,untreated}^{2}$	0.274(0.151,0.394)	0.154(0.052,0.229)	$\sigma_{2,untreated}^{2}$	0.154(0.052,0.229)
$\sigma_{3,untreated}^{2}$	0.223(0.076,0.361)	0.078(0.010,0.104)	$\sigma_{3,untreated}^{2}$	0.078(0.010,0.104)
**Model for G2/M phase drugs**			**Model for S Phase Treatment**	
*q*_1,*G*2/*M*_	0.575(0.548, 0.601)	0.619(0.536, 0.663)	*q*_1,*S*_	0.789(0.770, 0.807)
*q*_2,*G*2/*M*_	0.056(0.028, 0.089)	0.119(0.089, 0.135)	*q*_2,*S*_	0.218(0.139, 0.259)
*q*_3,*G*2/*M*_	0.130(0.091, 0.170)	0.102(0.073, 0.148)	*q*_3,*S*_	0.726(0.701, 0.816)
*q*_4,*G*2/*M*_	0.416(0.204, 0.621)	0.390(0.369, 0.428)	$m_{d,FR,S}^{max}$	0.443(0.425, 0.469)
$m_{d,FR,G2/M}^{max}$	0.599(0.417, 0.757)	0.143(0.039, 0.304)	$m_{d,UR,S}^{max}$	0.470(0.356, 0.656)
$m_{d,UR,G2/M}^{max}$	0.858(0.695, 0.926)	0.146(0.054, 0.398)	$m_{d,G2/Marrest}^{max}$	0.657(0.532, 0.789)
$m_{d,MS,G2/M}^{max}$	0.335(0.222, 0.560)	0.296(0.294, 0.297)	$m_{d,G1block}^{max}$	0.307(0.304, 0.311)
$m_{d,G1arrest}^{max}$	0.168(0.099, 0.232)	0.282(0.277, 0.307)	$\lambda_{d,G2/M}^{max}$	36.280(32.518, 41.305)
$m_{d,Sarrest}^{max}$	0.788(0.756, 0.804)	0.311(0.134, 0.708)	$\lambda_{UR,S}^{max}$	16.224(15.217, 17.447)
$\lambda_{d,G1}^{max}$	19.292(13.823, 27.256)	2.075 (1.282, 5.152)	$\lambda_{FR,S}^{max}$	45.185(44.301, 45.923)
$\lambda_{UR,G2/M}^{max}$	55.708(50.041, 65.969)	20.426(19.577, 21.346)	$\lambda_{G2/Marrest}^{max}$	43.440(23.375, 68.424)
$\lambda_{FR,G2/M}^{max}$	23.375(20.745, 26.851)	5.753(5.026, 6.190)	$\lambda_{G1block}^{max}$	80.704(78.610, 83.224)
$\lambda_{MS,G2/M}^{max}$	15.076(12.687, 16.995)	4.831(3.248, 10.583)	*b*_*S*_	14.039(11.376, 18.501)
$\lambda_{G1arrest}^{max}$	56.392(52.187, 60.217)	71.437(65.544, 72.767)	*b*_*d*,*S*_	10.744(6.819, 16.659)
$\lambda_{d,S}^{max}$	45.501(42.876, 48.687)	76.436(64.437, 86.657)	*n*_*S*_	1(fixed)
$\lambda_{Sarrest}^{max}$	41.429(35.456, 47.167)	59.984(57.204, 61.474)	*EC*_50,*d*,*S*_	10.066(fixed)
*b*_*G*2/*M*_	6.845(4.975, 8.348)	21.964(17.708, 23.707)	*EC*_50,*S*_	26.073(fixed)
*b*_*d*,*G*2/*M*_	8.969(6.641, 11.511)	18.483(17.867, 18.939)	*a*_*G*2/*M*_	4.205(2.340, 5.925)
*n*_*G*2/*M*_	1(fixed)	1(fixed)	*b*_*G*2/*M*_	4.089(1.710, 6.861)
*EC*_50,*d*,*G*2/*M*_	10.295(fixed)	5.802(fixed)	$\sigma_{1,treated}^{2}$	6.537e-04(5.973e-04, 6.796e-04)
*EC*_50,*G*2/*M*_	2.301(fixed)	12.023(fixed)	$\sigma_{2,treated}^{2}$	6.392e-04(5.956e-04, 6.709e-04)
*a*_*S*_	0.404(0.207, 0.616)	0.646(0.391, 0.918)	$\sigma_{3,treated}^{2}$	6.648e-04(6.334e-04, 6.844e-04)
*b*_*S*_	2.583(1.141, 5.161)	11.172(10.255, 12.736)		
$\sigma_{1,treated}^{2}$	8.052e-04(7.550e-04,8.515e-04)	8.398e-04(7.682e-04,8.921e-04)		
$\sigma_{2,treated}^{2}$	7.854e-04(7.375e-04, 8.381e-04)	8.726e-04(8.384e-04, 8.801e-04)’		
$\sigma_{3,treated}^{2}$	7.848e-04(7.386e-04, 8.446e-04)	8.479e-04(8.038e-04, 8.897e-04)		

### Treatment-specific models capture cell cycle dynamics observed in the literature

To calibrate parameters in the treated model, we adopt a two-step modeling strategy: (1) First, we determined the values of the parameter *EC*_50_ in the dose–response (Hill) function using publicly available cell viability and growth inhibition assay data. Specifically, the parameters *EC*_50,*d*,*i*_, for i={G2/M,S} in Eq (10) and Eq (12), are derived from cell viability assay data obtained from the Genomics of Drug Sensitivity in Cancer (GDSC) database [83]. The parameters *EC*_50,*i*_, for i={G2/M,S} in Eq (9) and Eq (11), are derived from growth inhibition assay data provided by the NCI Developmental Therapeutics Program (DTP) database. (2) Second, we fixed the *EC*_50_ parameters in the Hill function and calibrated the remaining model parameters using experimental data collected at a single drug concentration. Hill coefficients in *E*_*i*_, i={G2/M,S} (Eq (9) and Eq (11)) and *E*_*d*,*i*_, i={G2/M,S} (Eq (10) and Eq (12)) are fixed as 1.

When modeling treatment effects, we initiated the simulation at time 0 under control conditions and allowed the system to evolve until it reached $T_{SSD}$. By this point, the cell population has entered the exponential growth phase, and the cell cycle phase distribution stabilizes, matching the steady-state percentages observed in experimental data. At $T_{SSD}$, we introduced the treatment by modifying the transition matrix **m** and the diagonal distribution matrix **G**, which are then used to compute the generation expansion matrix 𝐌(t). A detailed description of this adjustment is provided in Eq (S1.36) of the Supporting Information S1 File. This modeling approach is consistent with common experimental protocols, where drug treatments are introduced during the exponential growth phase to evaluate their effects on actively dividing cells. Because our study is based on *in vitro* data, we assumed that the treatment effect persists throughout the simulation, consistent with experimental protocols that involve continuous drug exposure. A time step of 0.1 hours is used to balance computational efficiency with sufficient resolution in the simulated cell population dynamics.

**Fig 3:**
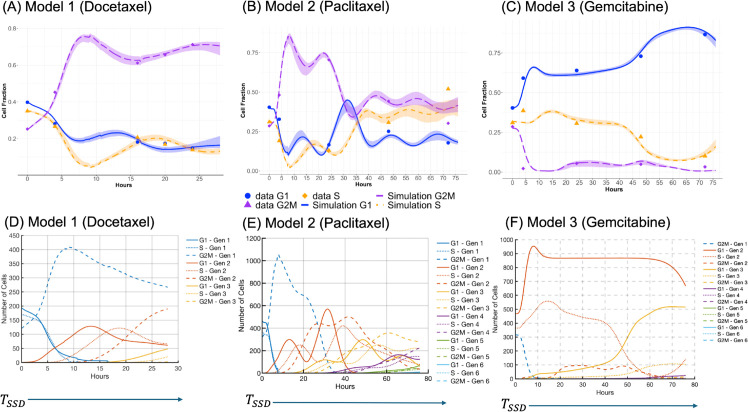
Simulated cell cycle dynamics and generational progression following docetaxel, paclitaxel, and gemcitabine treatments. Panels (A) through (C) illustrate the cell cycle dynamics under chemotherapeutic agents in three separate models respectively. Each panel in the top row shows the simulated cell cycle fractions (G1, S, and G2/M phases) alongside experimental data points over time, with shaded regions indicating the 50% credible intervals around the simulation curves. The solid lines represent the expected cell fractions in each phase calculated using Eq (S1.37) in Supporting Information S1 File, while the symbols (circles for G1, triangles for G2/M, and diamonds for S phase) denote experimental data points. Panels (D) through (F) provide a breakdown of the generational progression within the three models, highlighting the shifts in cell cycle distributions across multiple generations following treatment. The y-axis in panels (D) through (F) indicates the number of cells in each state after *T*_*SSD*_, computed from a branching process initiated by a single G1 ancestor cell at time zero.

The models accurately capture cell cycle progression dynamics under treatment with docetaxel, paclitaxel, and gemcitabine. Simulation results closely match experimental observations across the G1, S, and G2/M phases, as shown in panels A–C of Fig 3. Panels D–F of Fig 3 further present a generational breakdown of treatment response, revealing differential responses across cell generations. The elevated cell counts observed in early generations (e.g., G2/M generation 1 for G2/M phase drugs docetaxel and paclitaxel, and G1 generation 2 for S phase drug gemcitabine) arise because most directly impacted cells transition into either the unfaithful repair (UR) or faithful repair (FR) states. This observation is consistent with the parameter calibration results in Table 5 which shows that for G2/M phase drug, the probability of entering unfaithful repair in G2/M phase is higher than for other fates (*q*_1,*G*2/*M*_ = 0.575 (CI: 0.548–0.601) for docetaxel and *q*_1,*G*2/*M*_ = 0.619 (CI: 0.536–0.663) for paclitaxel). Because the UR state is elongated to allow for repair attempts, cells transiently accumulate there, leading to high early counts. Some G2/M cells instead undergo faithful repair and progress to the next phase, contributing to additional survivors. A similar trend can be observed in G1 cells arrested at the G1/S border (*q*_1,*S*_ = 0.789, CI: 0.770-0.807) for S phase drug gemcitabine. In later generations, cell counts decline because a substantial fraction of cells in the previous generations enter cell states in which death can be triggered. This reduction is further compounded by intergenerational transmission of damage, where daughter cells inherit stress or unresolved lesions from their mothers, and by the cumulative effects of direct drug-induced damage introduced during successive cycles.

### Estimated parameters reveal drug-specific patterns of arrest, repair, and cell death

The models with treatment incorporate various parameters to capture the complexities of drug action. These parameters include probabilities of cells entering different states after treatment, such as faithful DNA repair, unfaithful DNA repair, and mitotic slippage, as well as the death probabilities associated with these states. These parameters are estimated to capture the dynamic responses of cells to G2/M and S phase drugs, highlighting how checkpoint activation and residual DNA damage can slow down cell cycle progression. As shown in Table 5, which lists the mean values and 50% credible intervals (CI) for each parameter, paclitaxel and gemcitabine elicit distinct patterns of cell cycle arrest and DNA repair. In gemcitabine-treated cells, there is a high probability of arrest at the G1/S border (*q*_1,*S*_ = 0.789, CI: 0.770–0.807), unfaithful repair (*q*_2,*S*_ = 0.218, CI: 0.139–0.259) and faithful repair (*q*_3,*S*_ = 0.726, CI: 0.701–0.816). These cells also exhibit elevated death probabilities during both unfaithful ($m_{d,UR,S}^{\max}=0.470$, CI: 0.356–0.656) and faithful repair ($m_{d,FR,S}^{\max}=0.443$, CI: 0.425–0.469). In contrast, paclitaxel-treated cells are more likely to undergo cell death during S phase arrest in later generations ($m_{d,Sarrest}^{\max}=0.311$, CI: 0.134–0.708) and a higher probability to transition to unfaithful repair (*q*_1,*G*2/*M*_ = 0.619, CI: 0.536–0.663).

Docetaxel was modeled in the AGS gastric cancer cell line and is therefore not directly compared with the H460 NSCLC line used for paclitaxel and gemcitabine. However, it serves as an additional test case for the model. In AGS cells, docetaxel induces high death probabilities during both faithful ($m_{d,FR,G2/M}^{\max}=0.599$, CI: 0.417–0.757) and unfaithful repair ($m_{d,UR,G2/M}^{\max}=0.858$, CI: 0.695–0.926), as well as during arrest in the subsequent S phase ($m_{d,Sarrest}^{\max}=0.788$, CI: 0.756–0.804).

The posterior distributions of model parameters are shown in Fig D–F in Supplementary Information S2 File. As shown in those figures, not all parameters can be precisely identified from the available data. Accurate inference of these particular values could therefore benefit from sufficient experimental information, enhancing the precision and reliability of these parameter estimates.

### Treatment-induced DNA damage repair prolongs cell cycle duration in subsequent generations following treatment

**Fig 4:**
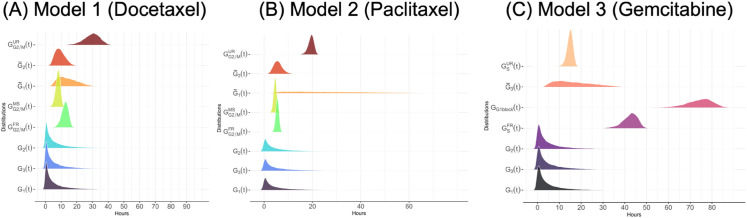
Ridgeline plots illustrating the distribution of cell cycle phase durations under control conditions and various treatment-induced states. (A) Model 1 (Docetaxel); (B) Model 2 (Paclitaxel); (C) Model 3 (Gemcitabine). The x-axis measures time in hours, and the y-axis represents the density of the distribution for each condition. *G*_*p*_(*t*),*p* = {1,2,3} represent the cell cycle time distributions for the G1, S, and G2/M phases, respectively. The cdf of the time elapsed in the unfaithful repair state are denoted as $G_{G2/M}^{UR}(t)$ for G2/M phase cells and $G_{S}^{UR}(t)$ for S phase cells. Similarly, $G_{G2/M}^{FR}(t)$ and $G_{S}^{FR}(t)$ represent the cdfs of the time elapsed in the faithful repair state for G2/M and S phase cells, respectively. ${\tilde{G}}_{p}(t)$ represents the cdf of the time spent in phase *p* during the new cell cycle after treatment. *G*_*G*1*block*_(*t*) represents the cdf of the time that cells spend when being blocked in the G1 phase after S phase treatment.

The comparative ridgeline plots presented in Fig 4 demonstrate the distinct effects of gemcitabine, docetaxel, and paclitaxel on the progression of the cell cycle. By comparing treatment-induced changes against control distributions for the G1, S, and G2/M phases, we observed that gemcitabine primarily prolongs the G1 and S phase duration of the treatment-hit cells. In contrast, docetaxel and paclitaxel predominantly affect the G2/M phase of the treatment-hit cells, as shown by the shifts in the distribution peaks and spreads. These drugs also impact cells generated post *T*_*SSD*_, with prolonged G1 (${\tilde{G}}_{1}(t)$) and S phases (${\tilde{G}}_{2}(t)$) under G2/M drugs and extended G2/M phases (${\tilde{G}}_{3}(t)$) under S phase drugs. This indicates long-term impacts on cell cycle progression that extend beyond the immediate treatment phases. The analysis further differentiates between faithful repair (FR) and unfaithful repair (UR). In H460 NSCLC cells treated with gemcitabine, UR displays tight distribution peaks, suggesting rapid but less accurate resolution that could introduce genomic instability, while FR shows prolonged phases, indicative of efficient and accurate DNA repair efforts aimed at preserving genomic stability. In contrast, paclitaxel-treated H460 cells exhibit the opposite trend: more time is spent in unfaithful repair than in faithful repair. This suggests that unresolved damage persists longer and cells remain in error-prone repair states. In AGS gastric cancer cells treated with docetaxel, more time is spent in UR and less time in FR. Taken together, the simulation results highlight that both the balance between faithful and unfaithful repair and the duration of these repair states vary across drugs and cell lines. In particular, a greater reliance on unfaithful repair may indicate vulnerability to treatments targeting NHEJ, while prolonged FR could signal a dependence on HR pathways. The choice between DNA repair pathways NHEJ and HR, however, is also regulated by other factors such as regulated expression and phosphorylation of repair proteins and chromatin modulation of repair factor accessibility [84]. Given the scope of this paper, we restricted our focus to how repair fidelity, specifically the distinction between faithful and unfaithful repair, shapes variability in treatment response.

### Global sensitivity analysis reveals model parameters that significantly influence the model simulation

We performed a Sobol global sensitivity analysis to evaluate how interactions among model parameters influence model outputs. The results, shown in Fig G and Fig H in Supporting Information S2 File identify the model parameters that significantly influence simulation outcomes for both the S phase drug and G2/M phase drug models. For the G2/M drug model, the parameters related to the G2/M checkpoint activation triggered by the G2/M phase drug and subsequent repair outcomes, such as *q*_1,*G*2/*M*_, *q*_2,*G*2/*M*_ and *q*_3,*G*2/*M*_, exhibited high sensitivity indices. This aligns with the critical role this checkpoint plays in mediating the cellular response to DNA damage. Similarly, in the S phase drug model, parameters governing repair pathways, such as the probability of unfaithful repair *q*_2,*S*_ and the probability of faithful repair *q*_3,*S*_, were identified as critical influencers of model behavior, reflecting how disruptions in DNA replication can strongly affect cell cycle progression. The analysis also revealed the importance of the scale parameters associated with the duration of unfaithful DNA repair triggered by drugs that affected the G2/M and S phases. Their roles in modulating output suggest they may serve as potential therapeutic targets. Adjusting these parameters to perturb DNA repair fidelity and checkpoint regulation could sensitize cancer cells to treatment. In particular, impairing repair fidelity may increase the vulnerability of cells to agents that act during DNA synthesis and mitosis, thereby enhancing drug efficacy in the S and G2/M phases.

### Effect of dose on the cell cycle dynamics

To systematically explore how varying chemotherapeutic doses influence cell cycle dynamics, we extended the model calibrated at a representative drug concentration to simulate population responses across a range of doses. A Hill function was used to capture how drug exposure parametrically scales the cdfs describing cell-state transition times and cell-death probabilities, whose parameters were estimated from the model fitted at a representative drug concentration. The Hill formulation was chosen as a phenomenological approximation to capture the saturable nature of drug effects and the graded transition between low and high response regimes, enabling interpolation across concentrations beyond those directly measured. The dose ranges for docetaxel were obtained from *in vitro* dose-response assays as detailed in [85], while those for paclitaxel and gemcitabine were based on the assays described in [48].

**Fig 5:**
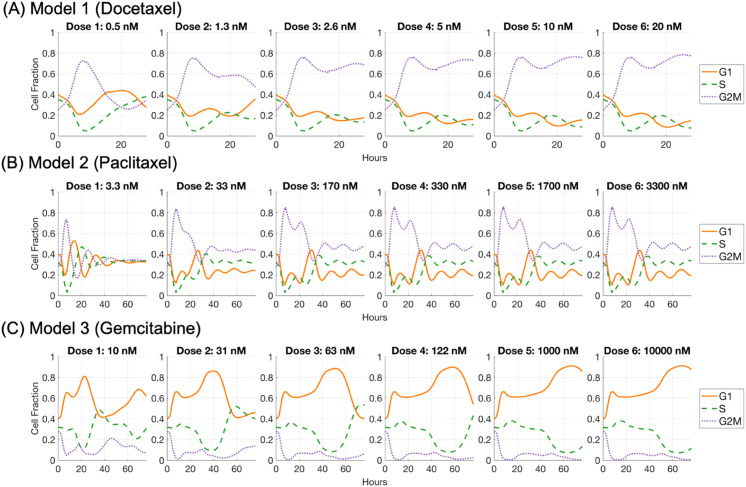
Dose-dependent cell cycle dynamics for the three chemotherapeutic models. Panels (A), (B), and (C) represent the temporal evolution of cell cycle phase distribution across a range of doses for docetaxel, paclitaxel, and gemcitabine widely adopted in *in vitro* dose-response studies. Each line within the panels corresponds to a specific phase of the cell cycle, with solid orange for the G1 phase (G1), dashed green for the S phase (S), and dotted purple for the G2/M phase (G2/M). The dose levels for each drug are shown above the respective plots, illustrating the resulting fluctuations in the distribution of cells across different phases over a span of hours post-treatment.

The simulation indicates the drug exposure modulates cell cycle behavior in a dose-dependent manner. As shown in Fig 5, the cell cycle distributions respond distinctly to increasing doses of docetaxel (Model 1), paclitaxel (Model 2), and gemcitabine (Model 3). At lower doses, cells show a degree of tolerance, maintaining near-normal cycling despite moderate fluctuations. This may reflect efficient DNA repair or the activation of survival signaling pathways. As the dosage increases, we saw more pronounced effects, particularly in the S and G2/M phases of the cell cycle. These findings suggest the presence of a dose threshold at which chemotherapeutic agents shift from transiently halting cell proliferation (cytostatic effect) toward inducing cell death (cytotoxic effect). This transition is reflected in increased cell accumulation during drug-treated phases, indicative of sustained checkpoint activation and arrest. When damage is extensive or persists beyond the cell’s repair capacity, these checkpoints can engage apoptotic pathways, reducing the cycling population.

This analysis helps delineate the range of doses associated with partial versus extensive cell cycle perturbation and identify both the minimum effective dose and the point at which increasing the dose ceases to enhance therapeutic benefit. In the case of gemcitabine and paclitaxel, these observations broadly align with growth inhibition curves reported in [48], where relative growth levels off after 1.7 μM for paclitaxel and 5 μM for gemcitabine. Future experimental work will be needed to validate the simulated dose–response behavior and refine the quantitative aspects of these predictions.

### Prediction of cell cycle kinetics under combination therapy

The combination of gemcitabine and paclitaxel is widely used as the first-line treatment for advanced breast cancer, with gemcitabine administered 1200 *mg*/*m*^2^ on days 1 and 8, and paclitaxel 175 *mg*/*m*^2^ on day 1 (before gemcitabine) in 21-day cycles for up to 10 cycles [86, 87]. This regimen has demonstrated significant efficacy in clinical settings, providing a strong foundation for its continued use and study. Motivated by its therapeutic relevance, we extended our modeling framework to study the effects of this combination in NSCLC cancer cells. Computational modeling provides an efficient and cost-effective platform to screen drug interactions and dose combinations, serving as a first step before conducting more resource-intensive *in vivo* experiments.

**Fig 6:**
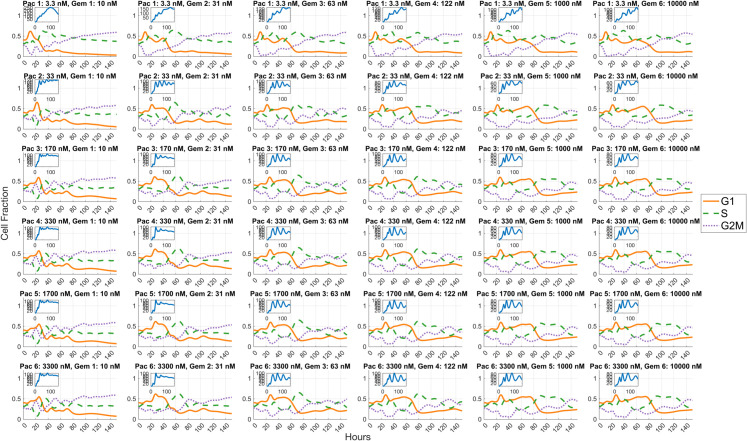
Cell cycle progression and cytotoxic response to concurrent administration of paclitaxel and gemcitabine across a range of concentrations. Each main plot depicts the fraction of cells in G1, S, and G2/M phases over time, while the inset plot illustrates the number of apoptotic cells initiated by cells at the treatment time. A 5-hour apoptotic duration was assumed based on peak caspase activation timing in H460 cells and the typically swift *in vivo* clearance by macrophages [89, 90].

The simulation results of concurrent administration of paclitaxel and gemcitabine are shown in Fig 6. The concurrent dosing simulations of paclitaxel and gemcitabine demonstrate a distinct dose-dependent interplay between the two drugs. The dose range used here is the same as the one in Section “Effect of dose on the cell cycle dynamics". As the concentration of gemcitabine escalates, there is a noticeable attenuation in the magnitude of initial apoptotic events, contrary to expectations based on the cytotoxic profile of paclitaxel alone. This observation indicates a potential antagonistic effect on cytotoxicity where higher levels of gemcitabine may mitigate the cytotoxicity induced by paclitaxel, in line with the empirical findings from a series of *in vitro* assays [88]. Additionally, the expansion of the apoptotic response over time with increasing gemcitabine doses suggests a possible alteration in cell death kinetics. As these simulations are obtained from a single-dose–calibrated framework using a Hill-type dose–effect relationship, the results represent model-based predictions of relative drug interactions under defined assumptions. These findings underscore the complexity of predicting outcomes in combination chemotherapy and highlight the need for careful consideration of dose timing and interaction effects when designing cancer treatment regimens.

## Discussion

Our computational framework employs a branching process based methodology to analyze the temporal dynamics of cell cycle progression in response to pharmacological interventions. The model’s parameters capture the modulation of DNA repair pathway activities, as well as checkpoint activation and cell death mechanisms, enabling the simulation of the phenotypic outcomes of cell cycle-specific therapies. Moreover, the model assumes statistical independence of cell transitions among cells to simplify the integration of biological processes such as checkpoint activation, DNA damage response, and apoptosis. We adopt this abstraction to enhance the interpretability and the model’s flexibility in simulating various treatment scenarios. For instance, we assume that cells carrying the residual damage inherited from their mother cells have the same transition rates to faithful repair and unfaithful repair as their mother cells. We acknowledge that such cells may be more susceptible to further damage during subsequent G2/M phases and could exhibit altered repair kinetics. Exploring the correlation between the extent of residual damage carried by the offspring cells and their rate of experiencing DNA damage repair is a valuable direction for future work. We also assume that all the treatment-induced processes share the same parameters in the sigmoid dose-effect curve. However, whether or not these processes truly share the same parameters remains an open question, and future experimental validation will be necessary to confirm or refine this assumption.

The model shows good alignment with empirical data and captures key features of drug-induced cell cycle perturbations. Simulation suggests that treatment-induced DNA damage repair can extend cell cycle duration in subsequent generations. Specifically, H460 NSCLC cells treated with gemcitabine tend to remain longer in faithful repair states, while those treated with paclitaxel exhibit prolonged unfaithful repair. Similarly, AGS gastric cancer cells treated with docetaxel also show extended time in unfaithful repair. However, it does not yet account for the role of regulatory checkpoint proteins that modulate how cells respond to chemotherapy. Their future integration, supported by experimental data, could enhance predictive accuracy. For example, proteins like Mad2 or BubR1 are known to influence the effectiveness of drugs like paclitaxel [91], and incorporating these molecular details, potentially through a biochemical network dynamics model that considers the cell cycle, such as [92–94], could enhance the model’s predictive power by allowing time-dependent protein concentration to govern death rates or transition probabilities. This is similar to hybrid modeling practices where an agent-based framework is coupled to a mechanistic model [95].

Unlike ODE-based frameworks that capture average population-level dynamics [96, 97], or stochastic and Markov approaches that capture variability but typically omit multi-generational inheritance of damage [98–100], our branching-process framework integrates cell-to-cell variability in progression with treatment-specific effects such as repair fidelity and intergenerational damage transmission. The flexibility of branching processes to incorporate different cell states and their distributions has led to applications in diverse biological contexts, including neurogenesis [32], immunogenesis [30], and blood cell differentiation [31]. Branching processes have also been employed to explore heterogeneous DNA damage; for example, Bastogne et al. [101] simulated radiotherapy-induced mutation accumulation *in vitro* by initializing cell states with different levels of proliferation, repair capacity, and genomic stability. By contrast, our framework focuses on how phase-specific chemotherapy affects cells directly, through induced damage, or indirectly, through the transmission of unresolved damage across generations. A natural extension would be to incorporate additional phenotypic states beyond cell cycle phases, enabling the model to capture how repair fidelity varies across phenotypes and to examine how these differences shape overall population dynamics. This requires higher-resolution experimental data, such as time-lapse microscopy or single-cell omics, to support model calibration and validation.

Simulating multiple dose levels and combination therapies has clear clinical relevance, as optimizing treatment schedules is essential for improving therapeutic outcomes. By enabling the exploration of diverse drug regimens, the framework offers a quantitative means to examine how chemotherapeutic agents interact and reshape tumor cell cycle dynamics. The model extends predictions across concentrations using a sigmoid response function, which represents a simplifying assumption that captures checkpoint arrest, mitotic slippage, and related outcomes along a shared DNA damage response axis. This formulation serves as an abstraction of a complex signaling network but provides a practical starting point for linking drug concentration to population-level behavior within a unified probabilistic framework. *In silico* simulations suggest that increasing drug concentrations shift cellular responses from transient delays to sustained arrest and apoptosis, reflecting a gradual transition from cytostatic to cytotoxic effects. Extending the framework to concurrent paclitaxel and gemcitabine treatment in NSCLC cells reveals a distinct dose-dependent interplay that can manifest as antagonism under certain in vitro conditions. Future work should explore how varying the sequence and timing of administration, such as staggered delivery or altered dosing intervals, might mitigate antagonism and improve therapeutic synergy.

Our model is implemented with *in vitro* experiments, offering a controlled setting to analyze drug-tumor interactions. It assumes continuous drug exposure, which simplifies the dynamics of drug activity and reduces the need for extensive parametrization. This assumption also makes the steady-state approximation for underlying biological processes more applicable. While this approach facilitates a concentrated study on the immediate cytotoxic effects of chemotherapeutic agents, it omits the pharmacokinetic processes and systemic variables present in *in vivo* systems, such as metabolic clearance, immune modulation, and heterogeneous tissue environments. Unlike the constant exposure in *in vitro* assays, *in vivo* drug administration typically follows episodic or time-varying profiles. These introduce additional complexity through ADME processes (i.e., absorption, distribution, metabolism, and excretion.) Accurately capturing these dynamics would require a compartmentalized pharmacokinetic model with time-dependent parameters. The dose-dependent dynamics in the model are captured using a Hill function, which is commonly used to describe sigmoidal drug response [97]. While this approach provides a tractable and interpretable framework for modeling single-drug and combination effects, it is based on empirical assumptions and does not account for mechanistic drug interactions or nonlinear pharmacodynamics. As such, the simulation results derived from the Hill-based combination model require further experimental validation.

In addition to these pharmacokinetic considerations, our model assumes uniform drug distribution, constant nutrient and oxygen availability, and excludes nonlinear crowding effects such as contact inhibition, all of which are reasonable for *in vitro* monolayer cultures but may not hold in *in vivo* tissue contexts. We also did not explicitly model spontaneous cell death or a G0 quiescent phase. Instead, we approximated temporary cell cycle stalling through stochastic G1-to-S phase transitions. While these simplifications allow us to focus on analytically tractable and biologically interpretable *in vitro* behavior, they may underestimate long-term population heterogeneity and growth suppression mechanisms such as contact inhibition and spontaneous cell death, which are likely to have a significant impact when adapting the model to *in vivo* settings.

## Supporting information

S1 File Model derivation. (PDF)

S2 File Global sensitivity analysis of treatment model and identifiability analysis of model parameters. (PDF)

S1 Fig Total number of cells after treatment from a branching process initiated by a single G1 ancestor cell at time zero. The plot shows the total number of cells following treatment with docetaxel, paclitaxel, or gemcitabine, at doses listed in Table 4 compared to baseline exponential-phase growth. The y-axis indicates the number of cells in each state after *T*_*SSD*_, as computed from a branching process initiated by a single G1 ancestor cell at simulation time 0.
(TIF)

S2 Fig Visualization of Eq (9), Eq (10), Eq (11), and Eq (12). Panel (A) shows the values of *E*_*G*2/*M*_ and *E*_*d*,*G*2/*M*_ for docetaxel at concentrations of 0.5, 1.3, 2.6, 5, 10, and 20 nM. Panel (B) shows the values of *E*_*G*2/*M*_ and *E*_*d*,*G*2/*M*_ for paclitaxel at concentrations of 3.3, 33, 170, 330, 1700, and 3300 nM. Panel (C) shows the values of *E*_*S*_ and *E*_*d*,*S*_ for gemcitabine at concentrations of 10, 31, 63, 122, 1000, and 10000 nM.
(TIF)

## References

[1] Cheung AH-K, Hui CH-L, Wong KY, Liu X, Chen B, Kang W, et al. Out of the cycle: impact of cell cycle aberrations on cancer metabolism and metastasis. Int J Cancer. 2023;152(8):1510–25. doi: 10.1002/ijc.34288 36093588

[2] Rottenberg S, Disler C, Perego P. The rediscovery of platinum-based cancer therapy. Nat Rev Cancer. 2021;21(1):37–50. doi: 10.1038/s41568-020-00308-y 33128031

[3] Pawlik TM, Keyomarsi K. Role of cell cycle in mediating sensitivity to radiotherapy. Int J Radiat Oncol Biol Phys. 2004;59(4):928–42. doi: 10.1016/j.ijrobp.2004.03.005 15234026

[4] Alghamian Y, Abou Alchamat G, Murad H, Madania A. Effects of *γ*-radiation on cell growth, cell cycle and promoter methylation of 22 cell cycle genes in the 1321 NI astrocytoma cell line. Adv Med Sci. 2017;62(2):330–7. doi: 10.1016/j.advms.2017.03.004 28511071

[5] Fisi V, Kátai E, Bogner P, Miseta A, Nagy T. Timed, sequential administration of paclitaxel improves its cytotoxic effectiveness in a cell culture model. Cell Cycle. 2016;15(9):1227–33. doi: 10.1080/15384101.2016.1158361 27104236 PMC4889271

[6] Ma C, Gurkan-Cavusoglu E. A comprehensive review of computational cell cycle models in guiding cancer treatment strategies. NPJ Syst Biol Appl. 2024;10(1):71. doi: 10.1038/s41540-024-00397-7 38969664 PMC11226463

[7] Pugh K, Davies M, Powathil G. A mathematical model to investigate the effects of ceralasertib and olaparib in targeting the cellular DNA damage response pathway. J Pharmacol Exp Ther. 2023;387(1):55–65. doi: 10.1124/jpet.122.001558 37391224

[8] Miao X, Koch G, Ait-Oudhia S, Straubinger RM, Jusko WJ. Pharmacodynamic modeling of cell cycle effects for gemcitabine and trabectedin combinations in pancreatic cancer cells. Front Pharmacol. 2016;7:421. doi: 10.3389/fphar.2016.00421 27895579 PMC5108803

[9] Gross SM, Mohammadi F, Sanchez-Aguila C, Zhan PJ, Liby TA, Dane MA, et al. Analysis and modeling of cancer drug responses using cell cycle phase-specific rate effects. Nat Commun. 2023;14(1):3450. doi: 10.1038/s41467-023-39122-z 37301933 PMC10257663

[10] Panetta JC, Evans WE, Cheok MH. Mechanistic mathematical modelling of mercaptopurine effects on cell cycle of human acute lymphoblastic leukaemia cells. Br J Cancer. 2006;94(1):93–100. doi: 10.1038/sj.bjc.6602893 16333308 PMC2361089

[11] Alkan O, Schoeberl B, Shah M, Koshkaryev A, Heinemann T, Drummond DC, et al. Modeling chemotherapy-induced stress to identify rational combination therapies in the DNA damage response pathway. Sci Signal. 2018;11(540):eaat0229. doi: 10.1126/scisignal.aat0229 30042127

[12] Chaffey GS, Lloyd DJB, Skeldon AC, Kirkby NF. The effect of the G1-S transition checkpoint on an age structured cell cycle model. PLoS One. 2014;9(1):e83477. doi: 10.1371/journal.pone.0083477 24416166 PMC3886982

[13] Clairambault J, Michel P, Perthame B. A mathematical model of the cell cycle and its circadian control. Mathematical Modeling of Biological Systems, Volume I. Boston, MA: Birkhauser. 2007. p. 239–51. 10.1007/978-0-8176-4558-8

[14] Liu Y-H, Bi J-X, Zeng A-P, Yuan J-Q. A population balance model describing the cell cycle dynamics of myeloma cell cultivation. Biotechnol Prog. 2007;23(5):1198–209. doi: 10.1021/bp070152z 17691814

[15] Billy F, Clairambaultt J, Fercoq O, Gaubertt S, Lepoutre T, Ouillon T, et al. Synchronisation and control of proliferation in cycling cell population models with age structure. Mathematics and Computers in Simulation. 2014;96:66–94. doi: 10.1016/j.matcom.2012.03.005

[16] Basse B, Ubezio P. A generalised age- and phase-structured model of human tumour cell populations both unperturbed and exposed to a range of cancer therapies. Bull Math Biol. 2007;69(5):1673–90. doi: 10.1007/s11538-006-9185-6 17361361

[17] Basse B, Baguley BC, Marshall ES, Joseph WR, van Brunt B, Wake G, et al. Modelling cell death in human tumour cell lines exposed to the anticancer drug paclitaxel. J Math Biol. 2004;49(4):329–57. doi: 10.1007/s00285-003-0254-2 15657794

[18] Chaffey GS, Lloyd DJB, Skeldon AC, Kirkby NF. The effect of the G1-S transition checkpoint on an age structured cell cycle model. PLoS One. 2014;9(1):e83477. doi: 10.1371/journal.pone.0083477 24416166 PMC3886982

[19] Yates CA, Ford MJ, Mort RL. A multi-stage representation of cell proliferation as a Markov process. Bull Math Biol. 2017;79(12):2905–28. doi: 10.1007/s11538-017-0356-4 29030804 PMC5709504

[20] Belluccini G, López-García M, Lythe G, Molina-París C. Counting generations in birth and death processes with competing Erlang and exponential waiting times. Sci Rep. 2022;12(1):11289. doi: 10.1038/s41598-022-14202-0 35789162 PMC9253354

[21] Falcetta F, Lupi M, Colombo V, Ubezio P. Dynamic rendering of the heterogeneous cell response to anticancer treatments. PLoS Comput Biol. 2013;9(10):e1003293. doi: 10.1371/journal.pcbi.1003293 24146610 PMC3798276

[22] James DW, Filby A, Brown MR, Summers HD, Francis LW, Rees P. Data driven cell cycle model to quantify the efficacy of cancer therapeutics targeting specific cell-cycle phases from flow cytometry results. Front Bioinform. 2021;1:662210. doi: 10.3389/fbinf.2021.662210 36303763 PMC9581040

[23] Altinok A, Lévi F, Goldbeter A. Identifying mechanisms of chronotolerance and chronoefficacy for the anticancer drugs 5-fluorouracil and oxaliplatin by computational modeling. Eur J Pharm Sci. 2009;36(1):20–38. doi: 10.1016/j.ejps.2008.10.024 19041394

[24] Bernard D, Mondesert O, Gomes A, Duthen Y, Lobjois V, Cussat-Blanc S, et al. A checkpoint-oriented cell cycle simulation model. Cell Cycle. 2019;18(8):795–808. doi: 10.1080/15384101.2019.1591125 30870080 PMC6527278

[25] Orlando DA, Iversen ES Jr, Hartemink AJ, Haase SB. A branching process model for flow cytometry and budding index measurements in cell synchrony experiments. Ann Appl Stat. 2009;3(4):1521–41. doi: 10.1214/09-AOAS264 21853014 PMC3156593

[26] Miao H, Jin X, Perelson AS, Wu H. Evaluation of multitype mathematical models for CFSE-labeling experiment data. Bull Math Biol. 2012;74(2):300–26. doi: 10.1007/s11538-011-9668-y 21681605 PMC3196768

[27] Kimmel M, Axelrod DE. Branching processes in biology. New York: Springer; 2015. 10.1007/978-1-4939-1559-0

[28] Hyrien O, Chen R, Mayer-Pröschel M, Noble M. Saddlepoint approximations to the moments of multitype age-dependent branching processes, with applications. Biometrics. 2010;66(2):567–77. doi: 10.1111/j.1541-0420.2009.01281.x 19508238 PMC2888915

[29] Hyrien O, Chen R, Zand MS. An age-dependent branching process model for the analysis of CFSE-labeling experiments. Biol Direct. 2010;5:41. doi: 10.1186/1745-6150-5-41 20569476 PMC2914727

[30] Hyrien O, Chen R, Zand MS. An age-dependent branching process model for the analysis of CFSE-labeling experiments. Biol Direct. 2010;5(1):41. doi: 10.1186/1745-6150-5-41 20569476 PMC2914727

[31] Nordon RE, Ko K-H, Odell R, Schroeder T. Multi-type branching models to describe cell differentiation programs. J Theor Biol. 2011;277(1):7–18. doi: 10.1016/j.jtbi.2011.02.006 21333658

[32] Li B, Sierra A, Deudero JJ, Semerci F, Laitman A, Kimmel M, et al. Multitype Bellman-Harris branching model provides biological predictors of early stages of adult hippocampal neurogenesis. BMC Syst Biol. 2017;11(Suppl 5):90. doi: 10.1186/s12918-017-0468-3 28984196 PMC5629620

[33] Syljuåsen RG, Jensen S, Bartek J, Lukas J. Adaptation to the ionizing radiation-induced G2 checkpoint occurs in human cells and depends on checkpoint kinase 1 and Polo-like kinase 1 kinases. Cancer Res. 2006;66(21):10253–7. doi: 10.1158/0008-5472.CAN-06-2144 17079442

[34] Cahuzac N, Studény A, Marshall K, Versteege I, Wetenhall K, Pfeiffer B, et al. An unusual DNA binding compound, S23906, induces mitotic catastrophe in cultured human cells. Cancer Lett. 2010;289(2):178–87. doi: 10.1016/j.canlet.2009.08.014 19758748

[35] Demarcq C, Bunch RT, Creswell D, Eastman A. The role of cell cycle progression in cisplatin-induced apoptosis in Chinese hamster ovary cells. Cell Growth Differ. 1994;5(9):983–93. 7819136

[36] Krenning L, van den Berg J, Medema RH. Life or death after a break: what determines the choice?. Mol Cell. 2019;76(2):346–58. doi: 10.1016/j.molcel.2019.08.023 31561953

[37] Arora M, Moser J, Phadke H, Basha AA, Spencer SL. Endogenous replication stress in mother cells leads to quiescence of daughter cells. Cell Rep. 2017;19(7):1351–64. doi: 10.1016/j.celrep.2017.04.055 28514656 PMC5533606

[38] Lezaja A, Altmeyer M. Inherited DNA lesions determine G1 duration in the next cell cycle. Cell Cycle. 2018;17(1):24–32. doi: 10.1080/15384101.2017.1383578 28980862 PMC5815429

[39] Barr AR, Cooper S, Heldt FS, Butera F, Stoy H, Mansfeld J, et al. DNA damage during S-phase mediates the proliferation-quiescence decision in the subsequent G1 via p21 expression. Nat Commun. 2017;8:14728. doi: 10.1038/ncomms14728 28317845 PMC5364389

[40] Petit J, Lambiotte R, Carletti T. Classes of random walks on temporal networks with competing timescales. Appl Netw Sci. 2019;4(1):1–20. doi: 10.1007/s41109-019-0204-6

[41] Petit J, Gueuning M, Carletti T, Lauwens B, Lambiotte R. Random walk on temporal networks with lasting edges. Phys Rev E. 2018;98(5). doi: 10.1103/physreve.98.052307

[42] Chong T, Sarac A, Yao CQ, Liao L, Lyttle N, Boutros PC, et al. Deregulation of the spindle assembly checkpoint is associated with paclitaxel resistance in ovarian cancer. J Ovarian Res. 2018;11(1):27. doi: 10.1186/s13048-018-0399-7 29618387 PMC5885411

[43] Wang J. A bivalent recombinant vaccine: a promising strategy against both SARS-CoV-2 variants and wild type of the virus. Signal Transduct Target Ther. 2021;6(1):278. doi: 10.1038/s41392-021-00691-4 34274941 PMC8285695

[44] Koche RP, Smith ZD, Adli M, Gu H, Ku M, Gnirke A, et al. Reprogramming factor expression initiates widespread targeted chromatin remodeling. Cell Stem Cell. 2011;8(1):96–105. doi: 10.1016/j.stem.2010.12.001 21211784 PMC3220622

[45] Raab M, Sanhaji M, Zhou S, Rödel F, El-Balat A, Becker S, et al. Blocking mitotic exit of ovarian cancer cells by pharmaceutical inhibition of the anaphase-promoting complex reduces chromosomal instability. Neoplasia. 2019;21(4):363–75. doi: 10.1016/j.neo.2019.01.007 30851646 PMC6407080

[46] Henriques AC, Silva PMA, Sarmento B, Bousbaa H. Antagonizing the spindle assembly checkpoint silencing enhances paclitaxel and Navitoclax-mediated apoptosis with distinct mechanistic. Sci Rep. 2021;11(1):4139. doi: 10.1038/s41598-021-83743-7 33603057 PMC7893169

[47] Balcer-Kubiczek EK, Attarpour M, Wang JZ, Regine WF. The effect of docetaxel (taxotere) on human gastric cancer cells exhibiting low-dose radiation hypersensitivity. Clin Med Oncol. 2008;2:301–11. doi: 10.4137/cmo.s463 21892291 PMC3161637

[48] Kroep JR, Giaccone G, Tolis C, Voorn DA, Loves WJ, Groeningen CJ, et al. Sequence dependent effect of paclitaxel on gemcitabine metabolism in relation to cell cycle and cytotoxicity in non-small-cell lung cancer cell lines. Br J Cancer. 2000;83(8):1069–76. doi: 10.1054/bjoc.2000.1399 10993656 PMC2363564

[49] Mishra S, Berah T, Mellan TA, Unwin HJT, Vollmer MA, Parag KV, et al. On the derivation of the renewal equation from an age-dependent branching process: an epidemic modelling perspective. arXiv preprint 2020. doi: 10.48550/arXiv.2006.16487

[50] Sherer E, Tocce E, Hannemann RE, Rundell AE, Ramkrishna D. Identification of age-structured models: cell cycle phase transitions. Biotechnol Bioeng. 2008;99(4):960–74. doi: 10.1002/bit.21633 17787014

[51] Murphy H, Jaafari H, Dobrovolny HM. Differences in predictions of ODE models of tumor growth: a cautionary example. BMC Cancer. 2016;16:163. doi: 10.1186/s12885-016-2164-x 26921070 PMC4768423

[52] Weber TS, Jaehnert I, Schichor C, Or-Guil M, Carneiro J. Quantifying the length and variance of the eukaryotic cell cycle phases by a stochastic model and dual nucleoside pulse labelling. PLoS Comput Biol. 2014;10(7):e1003616. doi: 10.1371/journal.pcbi.1003616 25058870 PMC4109856

[53] LeCam L. On the distribution of sums of independent random variables. Bernoulli 1713, Bayes 1763, Laplace 1813. Berlin, Germany: Springer; 1965. p. 179–202.

[54] Harris TE. The theory of branching processes. Springer; 1963.

[55] Cowan R. Branching process results in terms of moments of the generation-time distribution. Biometrics. 1985;41(3):681. doi: 10.2307/2531288 4074819

[56] Lonati L, Barbieri S, Guardamagna I, Ottolenghi A, Baiocco G. Radiation-induced cell cycle perturbations: a computational tool validated with flow-cytometry data. Sci Rep. 2021;11(1):925. doi: 10.1038/s41598-020-79934-3 33441727 PMC7806866

[57] Yang K, Guo R, Xu D. Non-homologous end joining: advances and frontiers. Acta Biochim Biophys Sin (Shanghai). 2016;48(7):632–40. doi: 10.1093/abbs/gmw046 27217473

[58] Levatić J, Salvadores M, Fuster-Tormo F, Supek F. Mutational signatures are markers of drug sensitivity of cancer cells. Nat Commun. 2022;13(1):2926. doi: 10.1038/s41467-022-30582-3 35614096 PMC9132939

[59] Birkelbach M, Ferraiolo N, Gheorghiu L, Pfäffle HN, Daly B, Ebright MI, et al. Detection of impaired homologous recombination repair in NSCLC cells and tissues. J Thorac Oncol. 2013;8(3):279–86. doi: 10.1097/JTO.0b013e31827ecf83 23399959 PMC3573529

[60] Konstantinopoulos PA, Ceccaldi R, Shapiro GI, D’Andrea AD. Homologous recombination deficiency: exploiting the fundamental vulnerability of ovarian cancer. Cancer Discov. 2015;5(11):1137–54. doi: 10.1158/2159-8290.CD-15-0714 26463832 PMC4631624

[61] Mutter RW, Riaz N, Ng CK, Delsite R, Piscuoglio S, Edelweiss M, et al. Bi-allelic alterations in DNA repair genes underpin homologous recombination DNA repair defects in breast cancer. J Pathol. 2017;242(2):165–77. doi: 10.1002/path.4890 28299801 PMC5516531

[62] Willers H, Azzoli CG, Santivasi WL, Xia F. Basic mechanisms of therapeutic resistance to radiation and chemotherapy in lung cancer. Cancer J. 2013;19(3):200–7. doi: 10.1097/PPO.0b013e318292e4e3 23708066 PMC3668666

[63] Rothkamm K, Krüger I, Thompson LH, Löbrich M. Pathways of DNA double-strand break repair during the mammalian cell cycle. Mol Cell Biol. 2003;23(16):5706–15. doi: 10.1128/MCB.23.16.5706-5715.2003 12897142 PMC166351

[64] Khongkow P, Gomes AR, Gong C, Man EPS, Tsang JW-H, Zhao F, et al. Paclitaxel targets FOXM1 to regulate KIF20A in mitotic catastrophe and breast cancer paclitaxel resistance. Oncogene. 2016;35(8):990–1002. doi: 10.1038/onc.2015.152 25961928 PMC4538879

[65] Weaver BA. How Taxol/paclitaxel kills cancer cells. Mol Biol Cell. 2014;25(18):2677–81. doi: 10.1091/mbc.E14-04-0916 25213191 PMC4161504

[66] Kuczler MD, Olseen AM, Pienta KJ, Amend SR. ROS-induced cell cycle arrest as a mechanism of resistance in polyaneuploid cancer cells (PACCs). Prog Biophys Mol Biol. 2021;165:3–7. doi: 10.1016/j.pbiomolbio.2021.05.002 33991583 PMC8511226

[67] Zasadil LM, Andersen KA, Yeum D, Rocque GB, Wilke LG, Tevaarwerk AJ, et al. Cytotoxicity of paclitaxel in breast cancer is due to chromosome missegregation on multipolar spindles. Sci Transl Med. 2014;6(229):229ra43. doi: 10.1126/scitranslmed.3007965 24670687 PMC4176609

[68] Li Y, Cucinotta FA. Modeling non-homologous end joining. J Theor Biol. 2011;283(1):122–35. doi: 10.1016/j.jtbi.2011.05.015 21635903

[69] Ramanathan B, Jan K-Y, Chen C-H, Hour T-C, Yu H-J, Pu Y-S. Resistance to paclitaxel is proportional to cellular total antioxidant capacity. Cancer Res. 2005;65(18):8455–60. doi: 10.1158/0008-5472.CAN-05-1162 16166325

[70] Mohiuddin M, Kasahara K. The mechanisms of the growth inhibitory effects of paclitaxel on gefitinib-resistant non-small cell lung cancer cells. Cancer Genomics Proteomics. 2021;18(5):661–73. doi: 10.21873/cgp.20288 34479918 PMC8441760

[71] Gonçalves A, Braguer D, Carles G, André N, Prevôt C, Briand C. Caspase-8 activation independent of CD95/CD95-L interaction during paclitaxel-induced apoptosis in human colon cancer cells (HT29-D4). Biochem Pharmacol. 2000;60(11):1579–84. doi: 10.1016/s0006-2952(00)00481-0 11077039

[72] Pienta KJ. Preclinical mechanisms of action of docetaxel and docetaxel combinations in prostate cancer. Semin Oncol. 2001;28(4 Suppl 15):3–7. doi: 10.1016/s0093-7754(01)90148-4 11685722

[73] Mackler NJ, Pienta KJ. Drug insight: use of docetaxel in prostate and urothelial cancers. Nat Clin Pract Urol. 2005;2(2):92–100; quiz 1 p following 112. doi: 10.1038/ncpuro0099 16474654

[74] Crown J, O’Leary M, Ooi W-S. Docetaxel and paclitaxel in the treatment of breast cancer: a review of clinical experience. Oncologist. 2004;9 Suppl 2:24–32. doi: 10.1634/theoncologist.9-suppl_2-24 15161988

[75] Hamed SS, Straubinger RM, Jusko WJ. Pharmacodynamic modeling of cell cycle and apoptotic effects of gemcitabine on pancreatic adenocarcinoma cells. Cancer Chemother Pharmacol. 2013;72(3):553–63. doi: 10.1007/s00280-013-2226-6 23835677 PMC3777243

[76] Plunkett W, Huang P, Xu YZ, Heinemann V, Grunewald R, Gandhi V. Gemcitabine: metabolism, mechanisms of action, and self-potentiation. 22:3–10. 7481842

[77] Plunkett W, Huang P, Gandhi V. Preclinical characteristics of gemcitabine. Anticancer Drugs. 1995;6 Suppl 6:7–13. doi: 10.1097/00001813-199512006-00002 8718419

[78] Mini E, Nobili S, Caciagli B, Landini I, Mazzei T. Cellular pharmacology of gemcitabine. Ann Oncol. 2006;17 Suppl 5:v7-12. doi: 10.1093/annonc/mdj941 16807468

[79] Petit J, Gueuning M, Carletti T, Lauwens B, Lambiotte R. Random walk on temporal networks with lasting edges. Phys Rev E. 2018;98(5). doi: 10.1103/physreve.98.052307

[80] Ma C, Almasan A, Gurkan-Cavusoglu E. Computational analysis of 5-fluorouracil anti-tumor activity in colon cancer using a mechanistic pharmacokinetic/pharmacodynamic model. PLoS Comput Biol. 2022;18(11):e1010685. doi: 10.1371/journal.pcbi.1010685 36395103 PMC9671373

[81] Cappella P, Tomasoni D, Faretta M, Lupi M, Montalenti F, Viale F, et al. Cell cycle effects of gemcitabine. Int J Cancer. 2001;93(3):401–8. doi: 10.1002/ijc.1351 11433406

[82] Vihola M. Robust adaptive Metropolis algorithm with coerced acceptance rate. Stat Comput. 2011;22(5):997–1008. doi: 10.1007/s11222-011-9269-5

[83] Yang W, Soares J, Greninger P, Edelman EJ, Lightfoot H, Forbes S, et al. Genomics of Drug Sensitivity in Cancer (GDSC): a resource for therapeutic biomarker discovery in cancer cells. Nucleic Acids Res. 2013;41(Database issue):D955-61. doi: 10.1093/nar/gks1111 23180760 PMC3531057

[84] Shrivastav M, De Haro LP, Nickoloff JA. Regulation of DNA double-strand break repair pathway choice. Cell Res. 2008;18(1):134–47. doi: 10.1038/cr.2007.111 18157161

[85] Paiva KLR, Radicchi MA, Báo SN. In vitro evaluation of NLS-DTX activity in triple-negative breast cancer. Molecules. 2022;27(15):4920. doi: 10.3390/molecules27154920 35956870 PMC9370415

[86] Delfino C, Caccia G, Riva Gonzáles L, Mickiewicz E, Rodger J, Balbiani L, et al. Gemcitabine/paclitaxel as first-line treatment of advanced breast cancer. Oncology (Williston Park). 2003;17(12 Suppl 14):22–5. 14768401

[87] Allouache D, Gawande SR, Tubiana-Hulin M, Tubiana-Mathieu N, Piperno-Neumann S, Mefti F, et al. First-line therapy with gemcitabine and paclitaxel in locally, recurrent or metastatic breast cancer: a phase II study. BMC Cancer. 2005;5:151. doi: 10.1186/1471-2407-5-151 16316459 PMC1315334

[88] Sui M, Xiong X, Kraft AS, Fan W. Combination of gemcitabine antagonizes antitumor activity of paclitaxel through prevention of mitotic arrest and apoptosis. Cancer Biol Ther. 2006;5(8):1015–21. doi: 10.4161/cbt.5.8.2909 16855376

[89] Danish L, Imig D, Allgöwer F, Scheurich P, Pollak N. Bcl-2-mediated control of TRAIL-induced apoptotic response in the non-small lung cancer cell line NCI-H460 is effective at late caspase processing steps. PLoS One. 2018;13(6):e0198203. doi: 10.1371/journal.pone.0198203 29927992 PMC6013189

[90] Darzynkiewicz Z, Li X, Bedner E. Use of flow and laser-scanning cytometry in analysis of cell death. Methods Cell Biol. 2001;66:69–109. doi: 10.1016/s0091-679x(01)66005-9 11396020

[91] Sudo T, Nitta M, Saya H, Ueno NT. Dependence of paclitaxel sensitivity on a functional spindle assembly checkpoint. Cancer Res. 2004;64(7):2502–8. doi: 10.1158/0008-5472.can-03-2013 15059905

[92] Erdem C, Mutsuddy A, Bensman EM, Dodd WB, Saint-Antoine MM, Bouhaddou M, et al. A scalable, open-source implementation of a large-scale mechanistic model for single cell proliferation and death signaling. Nat Commun. 2022;13(1):3555. doi: 10.1038/s41467-022-31138-1 35729113 PMC9213456

[93] Bouhaddou M, Barrette AM, Stern AD, Koch RJ, DiStefano MS, Riesel EA, et al. A mechanistic pan-cancer pathway model informed by multi-omics data interprets stochastic cell fate responses to drugs and mitogens. PLoS Comput Biol. 2018;14(3):e1005985. doi: 10.1371/journal.pcbi.1005985 29579036 PMC5886578

[94] Novák B, Tyson JJ. A model for restriction point control of the mammalian cell cycle. J Theor Biol. 2004;230(4):563–79. doi: 10.1016/j.jtbi.2004.04.039 15363676

[95] Ruiz-Martinez A, Gong C, Wang H, Sové RJ, Mi H, Kimko H, et al. Simulations of tumor growth and response to immunotherapy by coupling a spatial agent-based model with a whole-patient quantitative systems pharmacology model. PLoS Comput Biol. 2022;18(7):e1010254. doi: 10.1371/journal.pcbi.1010254 35867773 PMC9348712

[96] Iwamoto K, Hamada H, Eguchi Y, Okamoto M. Mathematical modeling of cell cycle regulation in response to DNA damage: exploring mechanisms of cell-fate determination. Biosystems. 2011;103(3):384–91. doi: 10.1016/j.biosystems.2010.11.011 21095219

[97] Alkan O, Schoeberl B, Shah M, Koshkaryev A, Heinemann T, Drummond DC, et al. Modeling chemotherapy-induced stress to identify rational combination therapies in the DNA damage response pathway. Sci Signal. 2018;11(540):eaat0229. doi: 10.1126/scisignal.aat0229 30042127

[98] Tzamali E, Tzedakis G, Sakkalis V. Modeling how heterogeneity in cell cycle length affects cancer cell growth dynamics in response to treatment. Front Oncol. 2020;10:1552. doi: 10.3389/fonc.2020.01552 33042800 PMC7518087

[99] Yates CA, Ford MJ, Mort RL. A multi-stage representation of cell proliferation as a Markov process. Bull Math Biol. 2017;79(12):2905–28. doi: 10.1007/s11538-017-0356-4 29030804 PMC5709504

[100] Sarmah D, Meredith WO, Weber IK, Price MR, Birtwistle MR. Predicting anti-cancer drug combination responses with a temporal cell state network model. PLoS Comput Biol. 2023;19(5):e1011082. doi: 10.1371/journal.pcbi.1011082 37126527 PMC10174488

[101] Bastogne T, Marchand J-L, Pinel S, Vallois P. A branching process model of heterogeneous DNA damages caused by radiotherapy in in vitro cell cultures. Math Biosci. 2017;294:100–9. doi: 10.1016/j.mbs.2017.09.006 29054768

