# Supplementary material for: Probabilistic modeling of cell cycle dynamics in response to cell cycle targeting chemotherapy drugs to guide treatment strategies
Source: PLoS Comput Biol. 2025 Dec 16;21(12):e1013790. doi: 10.1371/journal.pcbi.1013790 (PMC12707676; doi:10.1371/journal.pcbi.1013790)
Supplement: S1 File — (PDF) [file pcbi.1013790.s001.pdf]

Probabilistic modeling of cell cycle dynamics in response to cell cycle targeting chemotherapy drugs to guide treatment strategies

Chenhui Ma[1,*], Evren Gurkan-Cavusoglu[1]

[1]Department of Electrical, Computer and Systems Engineering, Case Western Reserve University, Cleveland, Ohio, United States of America

[*]Corresponding author: cxm590@case.edu

Supporting Information S1 File: Model Derivation

---

1 Branching process of dividing cells without treatment

The completion time of each phase in the cell cycle, denoted as τ, is treated as a random variable. A cell's entry into the next phase depends on its age in the current phase. The random variables τ_1, τ_2, and τ_3 (representing the completion times of the G1, S, and G2/M phases, respectively) are modeled using the gamma distribution. The choice of the gamma distribution on modeling cell cycle phase time is well justified by both empirical measurements and *in silico* simulations as in the study [1]. The function $\psi_i(\tau)$ in Eq. S1.1 denotes the probability density function (pdf) of the phase duration τ for the transition from phase i to phase $i+1$, with $\psi_1(\tau)$, $\psi_2(\tau)$, and $\psi_3(\tau)$ corresponding to the durations τ_1 (G1), τ_2 (S), and τ_3 (G2/M), respectively. The corresponding cumulative distribution functions (cdfs) are denoted by $G_1(t)$, $G_2(t)$, and $G_3(t)$.

$$\psi_i(\tau) = \frac{\beta_i^{\alpha_i}}{\Gamma(\alpha_i)}\tau^{\alpha_i-1}e^{-\beta_i\tau} \tag{S1.1}$$

Here, Γ represents the gamma function. β_i and α_i are the rate and shape parameters respectively. We simplify the parameterization of the gamma distribution by setting a common rate parameter, $\beta_i = \beta_0$, for all phases ($i = 1, 2, 3$). The utilization of a single rate parameter β_0 enables us to directly relate the mean cell cycle length and its variance to the sum of the shape parameters ($\sum_{i=1}^{3}\alpha_i$) and the common rate parameter β_0, through the relationships $\frac{\sum_{i=1}^{3}\alpha_i}{\beta 0}$ for the mean and $\frac{\sum_{i=1}^{3}\alpha_i}{\beta_0^2}$ for the variance. The values of α_i and β_0 were obtained by fitting the model to the literature data. The Laplace transform of $\psi_i(\tau)$ is given by

$$\hat{\psi}_i(s) = \frac{\beta_0^{\alpha_i}}{(s+\beta_0)^{\alpha_i}} \tag{S1.2}$$

We later use the Laplace transform in the derivation of formulas for the steady state of cell cycle fractions given in Eq S1.23.

In our model, we begin with an initial population of N_0 ancestor cells at time $t = 0$. While theoretically these ancestor cells could begin in any phase of the cell cycle, we primarily assume that all ancestors initiate from the G1 phase at time 0. This assumption represents a specific scenario within the broader framework of a multi-type branching process. To maintain generality, we also incorporated the possibility of ancestor cells starting in the S or G2/M phases in our calculations. Reflecting cell cycle biology, each G2/M cell produces exactly two offspring, while G1 and S cells advance sequentially. After completing all cell cycle phases with a random time T with cdf $G(T)$, each G2/M cell undergoes mitosis, producing two daughter cells that begin their own cycles. We denote the number of ancestor cells in the ith phase at time 0 as $N_0^i, i = 1, 2, 3$, and the sum of N_0^i is N_0. Each cell is assumed to behave independently of all others. In particular, cell cycle phase durations are assumed to be independent and identically distributed. The total number of cells ($\Phi(t)$) is the sum of the number of cells produced in the independent copy of the branching process initiated

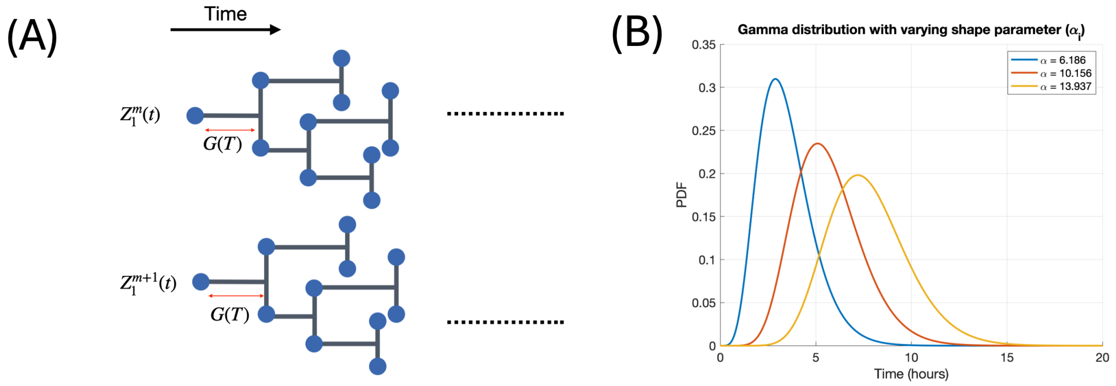

Figure A. Illustration of the branching process without death over time for cell divisions and Gamma distribution with varying shape parameter. (A) The random variable $Z_1^{(m)}(t)$ represents the number of cells in the mth independent realization of the process initiated by an ancestor cell in the G1 phase at time $t = 0$. The random time interval between branching events follows a distribution denoted by $G(T)$, which governs the timing of cell divisions in the process. As time progresses, cells proliferate according to this stochastic process, producing offspring and generating a growing population over successive generations. Created in BioRender. Ma, C. (2025) https://BioRender.com/wpctlm8. (B) Gamma distributions with varying shape parameters and a fixed rate parameter, as used in the model formulation.

by each ancestor cell in the ith phase. Mathematically, $\Phi(t)$ can be expressed as Eq S1.3.

$$\Phi(t) = \sum_{i=1}^{3} \sum_{m=1}^{N_0^i} Z_i^{(m)}(t) \tag{S1.3}$$

where $Z_i^{(m)}(t)$ is a random variable representing the number of cells produced in the mth independent copy of the process initiated by an ancestor cell in the ith phase at time 0. The illustration of the branching process of the cell cycle is shown in Fig A. Therefore, $\Phi(t)$ is the sum of a random number of iid random variables with nonnegative integer values. A useful tool for handling distributions of such random sums is the probability generating function (pgf), as it can be used to calculate the moments of the random variables. Let us assume that the initial phase of the mth ancestor cell at time 0 is the ith phase, and it will spend time τ in phase i. We can obtain the multivariate generating function of the number of cells of all phases present in the mth process initiated by an ancestor starting from i th phase $(\sum_{j=1}^{3} Z_{ij}^{m}(t))$, denoted as $g_i^{(m)}(\boldsymbol{s}, t)$, conditioned on the lifetime of the ith type ancestor cell (τ), where the lifetime is defined as the duration that a cell spends in phase i before transitioning to the next phase. Eq S1.4 is the multivariate version of conditioned pgf shown in [2].

$$g_i^m(\boldsymbol{s}, t) = \mathrm{E}\left[\prod_{j=1}^{3} s_j^{Z_{ij}^{(m)}(t)}\right] = \begin{cases} s_i, & t < \tau \\ f_i[g_1^m(\boldsymbol{s}, t-\tau), g_2^m(\boldsymbol{s}, t-\tau), g_3^m(\boldsymbol{s}, t-\tau)], & t \geq \tau \end{cases} \tag{S1.4}$$

Here $f_i(\boldsymbol{s})$ represents the multivariate progeny generating function of ith type cells. $\boldsymbol{s}$ is a state vector of arbitrary variables, denoted as $\boldsymbol{s} = [s_1, s_2, s_3]$, $|s_1| \leq 1, \ldots, |s_3| \leq 1$.

By removing the conditioning and integrating with respect to the cdf G_i of the length of the i-th phase, we obtain the following expression

$$g_i^m(\boldsymbol{s}, t) = s_i[1 - G_i(t)] + \int_0^t f_i[g_1^m(\boldsymbol{s}, t-\tau), g_2^m(\boldsymbol{s}, t-\tau), g_3^m(\boldsymbol{s}, t-\tau)]dG_i(\tau) \tag{S1.5}$$

Given that $Z_i^m(t)$ are iid variable, we can drop the superscript m for $g_i^m(\boldsymbol{s}, t)$. The pgf of the entire

process $(F(\boldsymbol{s},t))$ is

$$F(\boldsymbol{s},t) = \prod_{i=1}^{3} g_i(\boldsymbol{s},t)^{N_0^i} \tag{S1.6}$$

The marginal distribution for individuals in j phase is found by substituting the vector $\boldsymbol{s_j} = s_j\mathbf{e}_j + \sum_{\substack{l=1 \\ l\neq j}}^{3} \mathbf{e}_l$ into $F(\boldsymbol{s},t)$, where $\mathbf{e}_x$ is a 3-dimensional unit vector with the x-th component equal to 1. By utilizing the properties of probability generating functions, we can calculate the expected number of jth phase cells in the entire process at time t by taking the derivative of $F(\boldsymbol{s},t)$ with respect to s_j, which is Eq S1.7

$$\mathrm{E}[\Phi_j(t)] = \left.\frac{\partial F(\boldsymbol{s_j},t)}{\partial s_j}\right|_{s_j=1} \tag{S1.7}$$

The unique solution of Eq S1.7 is

$$\mathrm{E}[\Phi_j(t)] = \left.\sum_{i=1}^{3} N_0^i g_i(\boldsymbol{s}_j,t)^{N_0^i-1}\frac{\partial g_i(\boldsymbol{s}_j,t)}{\partial s_j} \prod_{\substack{q=1..3 \\ q\neq i}} g_q(\boldsymbol{s}_j,t)^{N_0^q}\right|_{s_j=1} \tag{S1.8}$$

Given that the generating functions $g_i(\boldsymbol{s}_j,t)$ evaluate to 1 at $s_j = 1$, we can get

$$\mathrm{E}[\Phi_j(t)] = \left.\sum_{i=1}^{3} N_0^i \frac{\partial g_i(\boldsymbol{s}_j,t)}{\partial s_j}\right|_{s_j=1} \tag{S1.9}$$

Next, we solve $\left.\frac{\partial g_i(\boldsymbol{s}_j,t)}{\partial s_j}\right|_{s_j=1}$ (denoted it as $M_{ij}(t)$) based on Eq S1.5, and call it the expected number of cells in j th phase at time t, in the process initiated by the ancestor cell in phase i [3].

$$M_{ij}(t) = \int_0^t \sum_{k=1}^{3} m_{ik} M_{kj}(t-\tau)dG_i(\tau) + \delta_{ij}\left[1 - G_i(t)\right] \tag{S1.10}$$

where m_{ij} is the expected number of progeny in phase j of a cell in phase i. $\delta_{i,j} = 1$, if $i = j$.

Next, we extended the above analysis by including generation k in calculating the expected number of cells in jth phase at time t. We add the generation factor k in $M_{ij}(t)$. The expected number of cells in phase j in generation k_2, initiated by cells in phase i in generation k_1, is denoted as $M_{i+3(k_1-1),j+3(k_2-1)}(t)$. For example, to determine how many S-phase cells in the second generation arise from a G1 cell in the first generation, we calculate $M_{1,5}(t)$. In general, $M_{i+3(k_1-1),,j+3(k_2-1)}(t)$ is obtained using the convolution integral given in Eq. S1.11.

$$\begin{aligned} M_{i+3(k_1-1),j+3(k_2-1)}(t) = &\int_0^t \sum_{n=1}^{3}\sum_{k'=k_1}^{k_2} m_{i+3(k_1-1),n+3(k'-1)} M_{n+3(k'-1),j+3(k_2-1)}(t-\tau)dG_i^{k_1}(\tau) \\ &+ \delta_{i+3(k_1-1),j+3(k_2-1)}\left[1 - G_i^{(k_1)}(t)\right], k_1 >= 1, k_2 >= 1 \end{aligned} \tag{S1.11}$$

in which $m_{i+3(k_1-1),n+3(k'-1)}$ represents the expected number of nth phase progeny in k'th generation produced by ith phase cells in k_1th generation such that $m_{1+3(k_1-1),2+3(k_1-1)} = 1, m_{2+3(k_1-1),3+3(k_1-1)} = 1, m_{3+3(k_1-1),1+3k_1} = 2$. The indicator $\delta_{i+3(k_1-1),j+3(k_2-1)} = 1$, if $i + 3(k_1 - 1) = j + 3(k_2 - 1)$. The first term in Eq S1.11 represents the expected number of cells of type $j + 3(k_2 - 1)$ at time t, produced from cells of type $i + 3(k_1 - 1)$ via intermediate cell states, integrating their propagation over time. The second term, $\delta_{i+3(k_1-1),j+3(k_2-1)}\left[1 - G_i^{(k_1)}(t)\right]$, accounts for the probability that a cell remains in the same state $i + 3(k_1 - 1)$ up to time t. Here,

the Kronecker delta (δ) ensures this contribution is non-zero only when the indices coincide, while $[1 - G_i^{(k_1)}(t)]$ gives the probability that a cell has not yet exited its current state by time t. To avoid confusion, we note that in the following equations, the indices i and j encode the generation information.

We then formulated Eq S1.11 in a matrix representation by employing the Lebesgue-Stieltjes integral, a mathematical approach essential for the convolution of functions within our model. We designate the matrix $\mathbf{M}$ as the cell lineage propagation matrix. For the Lebesgue-Stieltjes integral, when $A(t)$ and $B(t)$ are right-continuous functions with locally bounded variation on the interval $[0, \infty)$, their convolution is defined as $A(t) * B(t) = \int_0^t A(t - \tau) d_\tau B(\tau)$. This integral is particularly useful in our context as it captures the cumulative interaction between $A(t)$ and $B(t)$, where $B(t)$ is treated as a distribution function. The matrix form of Eq S1.11 is

$$\mathbf{M}(t) = (\mathbf{G}(t)\mathbf{m}) * (\mathbf{M}(t)) + [\mathbf{I} - \mathbf{G}(t)] \tag{S1.12}$$

in which each element $\mathbf{M}_{ij}(t)$ in matrix $\mathbf{M}$ represents the expected number of progeny of cell of type j produced by a cell of type i. $\mathbf{G}(t) = \mathrm{diag}\left(G_1(t), \ldots, G_K(t)\right), K > 1$ represents a diagonal matrix with each diagonal component representing the cdf of time each cell spends in state i. $\mathbf{m}$ represents the transition matrix, and $m_{ij}(t)$ is the expected number of progeny of a cell of type j produced by a cell of type i. $\mathbf{I}$ represents the identity matrix.

The product term $\mathbf{G}(t)\mathbf{m}$ can be considered as the linear integral (convolution) operator acting on $\mathbf{M}(t)$. We used $\mathcal{T}$ to represent $\mathbf{G}(t)\mathbf{m}$ for further demonstration. In this case, Eq S1.12 can also be written as

$$\mathbf{M}(t) = \mathcal{T}\mathbf{M}(t) + [\mathbf{I} - \mathbf{G}(t)] \tag{S1.13}$$

where $\mathcal{T}$ is the linear integral (convolution) operator acting on $\mathbf{M}(t)$.

Using the Laplace transform to solve the integral function Eq S1.12 is complicated. Instead, we can solve directly in the time domain by applying the Neumann series. According to Neumann's Lemma, if $\mathcal{T}$ is a linear bounded operator on a Banach space X and $\|\mathcal{T}\| = \sup_{\|x\| \le 1} \|\mathcal{T}x\| < 1$, then $I - \mathcal{T}$ is invertible. The inverse is expressed by the Neumann series as shown in Eq S1.14.

$$(I - \mathcal{T})^{-1} = \sum_{k=0}^{\infty} \mathcal{T}^k = I + \mathcal{T} + \mathcal{T}^2 + \ldots \tag{S1.14}$$

Hence, Eq S1.12 can be calculated as

$$\begin{aligned} \mathbf{M}(t) &= (I - \mathcal{T})^{-1}[\mathbf{I} - \mathbf{G}(t)] \\ &= \sum_{k=0}^{\infty} \mathcal{T}^k [\mathbf{I} - \mathbf{G}(t)] \end{aligned} \tag{S1.15}$$

In general, the Neumann series does not offer a practical way for computing $(I - \mathcal{T})^{-1}$ since it involves an infinite number of terms. In our analysis, the series was cut after $3 \times n_{gen}$ iterations. n_{gen} represents the number of generations.

Plugging in $\mathbf{G}(t)\mathbf{m}$, Eq S1.12 can be calculated as Eq S1.16.

$$\begin{aligned} \mathbf{M}(t) &= \sum_{k=0}^{3n_{gen}} (\mathbf{Gm})^{*k}(t) * [\mathbf{I} - \mathbf{G}(t)] \\ &= \sum_{k=0}^{3n_{gen}} \int \Big(\mathbf{I} - \mathbf{G}(t - u)\Big) d(\mathbf{Gm})^{*k}(u) \end{aligned} \tag{S1.16}$$

where $(\mathbf{Gm})^{*k}(t)$, a matrix in $\in \mathbb{R}^{(3n_{gen}) \times (3n_{gen}) \times n_T}$, represent the k-fold convolution (in the form $\int f(t - s) dg(s)$) of the distribution function $\mathbf{G}(t)\mathbf{m}$ with itself. Here, n_{gen} denotes the number of generations, and n_T represents the number of time points. Then, the iterates of $\mathbf{G}(t)\mathbf{m}$ act on $\mathbf{I} - \mathbf{G}$. The element $[(\mathbf{G})^{*k}(t)]_{i,i+k}$ in the three-dimensional diagonal matrix corresponds to the distribution

of time required for cells in phase $\mod(i+k,3)$ in $ceil((i+k)/3)$th generation to originate from cells in phase $\mod(i,3)$ in $ceil(i/3)$ th generation. Here, $\mod()$ returns the remainder after division of one number by another. $ceil()$ rounds a number up to the nearest integer. More generally, $[(\mathbf{G})^{*k}]_{i,i+k}$ gives probability distribution for the random variable T_k, which is the total time $T_k = \sum_{t=i}^{i+k-1} T_{t,t+1}$ taken to transition from state i to state $i+k$. Each $T_{t,t+1}$ in the summation represents the time duration between two consecutive phases. The pseudocode for computing $\mathbf{M}(t)$ (Eq S1.16) is shown in box 1.

Algorithm 1 Compute Matrix $\mathbf{M}(t)$

Initialization:
Set number of generations: num_gen
Set total state dimension: $TotalState = 3 \times num\_gen + 1$
Set number of time steps: num_t
Initialize $\mathbf{I} - \mathbf{G}$ (identity matrix minus $\mathbf{G}$)
Initialize $\mathbf{Gm}$ (matrix product of $\mathbf{G}$ and $\mathbf{m}$)
Set counter $k = 0$
while $k \leq TotalState$ **do**
  if $k = 0$ **then**
    Set next matrix $\mathbf{M}^k_{\text{next}}(t) \leftarrow \mathbf{I} - \mathbf{G}$
  else if $k = 1$ **then**
    Set $\mathbf{M}_{\text{curr}}(t) \leftarrow \mathbf{M}_{\text{next}}(t)$
    Set transition matrix $\mathbf{T}^k_{\text{next}}(t) \leftarrow \mathbf{Gm}$
    Initialize $\mathbf{M}^k_{\text{next}}(t) \leftarrow \mathbf{0}_{TotalState \times TotalState \times num\_t}$
    for $i = 1$ to $TotalState$ **do**
      if $i + k < TotalState$ **then**
        Compute convolution:

$$\mathbf{M}^k_{\text{next}}(i, i+k, :) \leftarrow (\mathbf{I} - \mathbf{G})(i+k, i+k, :) * \frac{\mathrm{d}\mathbf{T}^k_{\text{next}}(i, i+k, :)}{\mathrm{d}t} \Delta t$$

      end if
    end for
    Update next matrix $\mathbf{M}_{\text{next}}(t) \leftarrow \mathbf{M}_{\text{curr}}(t) + \mathbf{M}^k_{\text{next}}(t)$
  else
    Set $\mathbf{M}_{\text{curr}}(t) \leftarrow \mathbf{M}_{\text{next}}(t)$
    Set $\mathbf{T}^k_{\text{curr}}(t) \leftarrow \mathbf{T}^k_{\text{next}}(t)$
    Initialize $\mathbf{T}^k_{\text{next}}(t) \leftarrow \mathbf{0}_{TotalState \times TotalState \times num\_t}$
    for $i = 1$ to $TotalState$ **do**
      if $i + k < TotalState$ **then**
        Compute convolution:

$$\mathbf{T}^k_{\text{next}}(i, i+k, :) \leftarrow \mathbf{T}^k_{\text{curr}}(i, i+k-1, :) * \frac{\mathrm{d}\mathbf{Gm}(i+k-1, i+k, :)}{\mathrm{d}t} \Delta t$$

      end if
    end for
    Initialize $\mathbf{M}^k_{\text{next}}(t) \leftarrow \mathbf{0}_{TotalState \times TotalState \times num\_t}$
    for $i = 1$ to $TotalState$ **do**
      if $i + k < TotalState$ **then**
        Compute convolution:

$$\mathbf{M}^k_{\text{next}}(i, i+k, :) \leftarrow (\mathbf{I} - \mathbf{G})(i+k, i+k, :) * \frac{\mathrm{d}\mathbf{T}^k_{\text{next}}(i, i+k, :)}{\mathrm{d}t} \Delta t$$

      end if
    end for
    Update next matrix $\mathbf{M}_{\text{next}}(t) \leftarrow \mathbf{M}_{\text{curr}}(t) + \mathbf{M}^k_{\text{next}}(t)$
  end if
  Increment counter $k \leftarrow k + 1$
end while
  return $\mathbf{M}_{\text{next}}(t)$

Once we solve the derivative part of Eq S1.9, we can get the closed-form expression of $E[\Phi_j(t)]$. Since it is assumed that the branching process is initiated by G1-phase cells in the first generation, only the top row of matrix $\mathbf{M}(t)$ is required. Hence, the expected number of cells in jth phase across all

the n_{gen} generations is described as

$$E[\Phi_j(t)] = N_0^1 \sum_{k=1}^{n_{gen}} M_{1,3(k-1)+j}(t) \tag{S1.17}$$

The cell fraction in jth phase is expressed as $\mathrm{E}\left[\Phi_j(t)\right]/(\mathrm{E}[\Phi_1(t)] + \mathrm{E}[\Phi_2(t)] + \mathrm{E}[\Phi_3(t)])$, which is used to evaluate the goodness-of-fit.

Next, we examined the asymptotic behavior of the cell population with size $\Phi(t)$, which originates from initial cells, each initiating an independent branching process with variable lifetimes. In the long-time limit ($t \gg 1$), and under the assumptions of unlimited space and nutrients and no therapeutic perturbations, the population approaches an exponential growth regime in which the relative densities of cell cycle phases converge to stationary fractions [4]. Accordingly, the expected number of cells as $t \gg 1$ can be expressed by Eq. S1.18.

$$E[\Phi(t)] \sim \theta \exp(\gamma t) \text{ as } t \gg 1 \tag{S1.18}$$

in which γ is the mean growth rate of the cell population. θ is a scaling factor that accounts for the initial conditions of the system. The doubling time t_d of an exponentially growing population is expressed as

$$t_d = \frac{\ln 2}{\gamma}, \tag{S1.19}$$

where γ is the Malthusian parameter, which is also the positive root of Eq S1.20.

$$m\hat{f}(\gamma) = 1 \tag{S1.20}$$

in which $\hat{f}(\gamma)$ is the Laplace transform of the density of the cell cycle length. m denotes the mean of the number of daughter cells produced by each cell, kept as 2. γ from Eq S1.20 can be approximated by inverting a series expansion of Eq S1.20 [5]. Ridout et al. adopted an alternative approach to approximate γ [6], which is reported to be accurate as compared to the exact numerical solution of Eq S1.20 and doesn't require an estimate of the skewness parameter of the cell life length. Substituting the mean and variance of cell cycle duration T into the approximate solution of γ and θ shown in [6], we can get

$$\gamma \approx \frac{\ln(2)}{\sum_{i=1}^{3} \alpha_i/\beta_0} + \frac{(\ln 2)^2 \beta_0}{2(\sum_{i=1}^{3} \alpha_i)^2} \tag{S1.21}$$

$$\begin{aligned} \ln(\theta) &\approx \left(\frac{1}{\sum_{i=1}^{3} \alpha_i} - 1\right) \frac{\ln 2}{2} + \frac{(\ln 2)^2}{24} \left[\frac{1}{\left(\sqrt{\sum_{i=1}^{3} \alpha_i}\right)^3} \left(\frac{15}{\sqrt{\sum_{i=1}^{3} \alpha_i}} - \frac{16}{\sqrt{\sum_{i=1}^{3} \alpha_i}}\right) + 1\right] \\ &\approx \left(\frac{1}{\sum_{i=1}^{3} \alpha_i} - 1\right) \frac{\ln 2}{2} + \frac{(\ln 2)^2}{24} \left[-\frac{1}{\left(\sum_{i=1}^{3} \alpha_i\right)^2} + 1\right] \end{aligned} \tag{S1.22}$$

Cowan approximated the fractions of cells in different phases in terms of moments of the life length of cells and of the distribution of duration in cell cycle phases and suggested that there is a numerical relationship between the doubling time and the distribution of cell cycle duration (T) [5]. In [5], the expression for probabilities that a cell is in two hypothetical phases is provided, which can be regarded as the fraction of cells in cell cycle phases when $N(t)$ is large enough. Here, we extended the two-phase analysis to three phases. Eq S1.23 provides a numerical solution of the constant percentage

of cells in the G1, S, and G2/M phases when the population is in the asymptotic state.

$$p_{G1} = \frac{m\left[1-\hat{\psi}_1(\gamma)\right]}{m-1} = 2\left[1-\Big(\frac{\beta_0}{\gamma+\beta_0}\Big)^{\alpha_1}\right]$$
$$p_S = \frac{m\left[\hat{\psi}_1(\gamma)-\hat{\psi}_1(\gamma)\hat{\psi}_2(\gamma)\right]}{m-1} = 2\left[\Big(\frac{\beta_0}{\gamma+\beta_0}\Big)^{\alpha_1} - \Big(\frac{\beta_0}{\gamma+\beta_0}\Big)^{(\alpha_1+\alpha_2)}\right]$$
$$p_{G2/M} = \frac{m\left[\hat{\psi}_1(\gamma)\hat{\psi}_2(\gamma)-\hat{\psi}_1(\gamma)\hat{\psi}_2(\gamma)\hat{\psi}_3(\gamma)\right]}{m-1} = 2\left[\Big(\frac{\beta_0}{\gamma+\beta_0}\Big)^{(\alpha_1+\alpha_2)} - \Big(\frac{\beta_0}{\gamma+\beta_0}\Big)^{(\alpha_1+\alpha_2+\alpha_3)}\right] \tag{S1.23}$$

where the derivation for Eq S1.23 is shown in Section 'Derivation of steady state'.

2 Continuous time random walk (CTRW) model

To investigate how inherited unrepaired DNA damage affects cell cycle progression, we employed the continuous time random walk (CTRW) model [7, 8]. This approach allowed us to characterize: (1) G1-phase daughter cells inheriting DNA lesions from mother cells exposed to drug treatment; (2) S-phase daughter cells entering S with unresolved lesions that persisted despite attempted repair in the preceding G1 phase; and (3) G2/M-phase cells that had been exposed to S-phase-specific drugs during their S phase. In this framework, the CTRW model serves as a probabilistic tool to simulate cell transitions in daughter cells as a form of a random walk on an acyclic graph. The "walker" represents individual cells navigating through a network of "nodes", with each node symbolizing a specific cell cycle phase. The "edges" between these nodes represent possible transitions from one state to another, governed by probabilities and timing that reflect treatment-related influences. The duration of the cell cycle phases for these daughter cells is influenced by three competing factors: (1) the intrinsic waiting time imposed by the walker, representing the duration cells typically spend in the individual phase without treatment; (2) the downtime on edges, capturing the delays caused by checkpoint activation and DNA damage responses; and (3) the uptime on edges, representing recovery period following repair of inherited drug-induced damage. Employing the CTRW model in this focused manner allows us to investigate the intricacies of cell cycle progression in daughter cells under the impact of inherited DNA damage. This approach provides a nuanced understanding of the timing and duration of the cell cycle phase transitions influenced by unresolved DNA damage. The detailed mathematical analysis is shown as follows. Figure B depicts an example scenario of the CTRW process of S phase cells as they undergo DNA damage repair and successfully transition to the next phase.

Each node n_i $(i = 1, 2, 3)$ in the random walk network represents a cell cycle phase: G1 (n_1), S (n_2), and G2/M (n_3). An additional node n_d captures apoptosis to model treatment-induced cell death. We chose the Weibull distribution for the downtime distribution of edge n_d from n_i (i.e., $X_{D,n_d\leftarrow n_i}$) to capture the process of triggering the programmed cell death caused by DSBs beyond the repair capacity (Eq S1.24). The Weibull distribution is particularly advantageous as it can represent a range of different distribution shapes depending on its parameters, making it suitable for modeling the variability in cellular responses to damage over different doses.

$$D_d(t) = \frac{b_d}{\lambda_d}\left(\frac{t}{\lambda_d}\right)^{b_d-1} e^{-(t/\lambda_d)^{b_d}} \tag{S1.24}$$

Please refer to Table 2 in main text for the meaning of the parameters.

The downtime (X_D) for all edges within the daughter cells' cell cycles, excluding those progressing towards cell death (n_d), is characterized by a Weibull distribution, detailed in Eq S1.25. The uptime (X_U) for all edges, including those leading to n_d, follows an exponential distribution with a probability density function as specified in Eq S1.26. It is assumed that the distributions governing both uptime and downtime remain consistent across various generations of daughter cells that experience cell cycle arrest.

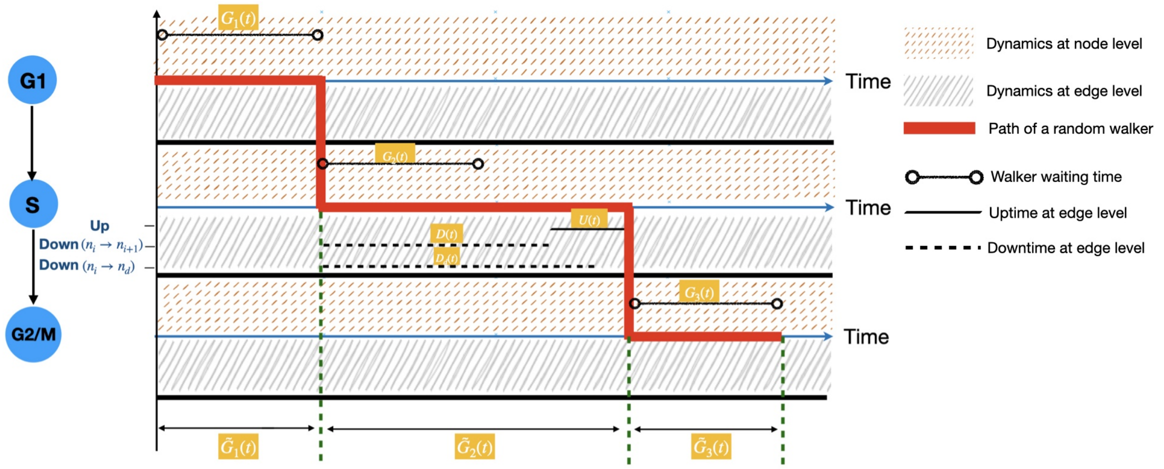

Figure B. Schematic representation of a continuous-time random walk (CTRW) on a cell cycle network. The walker (a cell) starts in the G1 phase and progresses through the S and G2/M phases, with waiting times $G_1(t), G_2(t)$, and $G_3(t)$ governing phase transitions. These waiting times imposed by the walker represent phase-specific durations from a baseline model without treatment. In the S phase, the walker encounters a temporary arrest due to unresolved double-strand breaks (DSBs), indicated by the dashed lines representing downtime, during which edges $n_i \to n_{i+1}$ and $n_i \to n_d$ are inactive. When downtime at edge $n_i \to n_{i+1}$ ends, but remains active for $n_i \to n_d$, the walker resumes movement, transitioning to the next phase via edge activation $U(t)$ (solid line), as shown by the red path. This diagram illustrates the interaction between cellular repair mechanisms and phase transitions in response to treatment-induced damage at both the node and edge levels.

$$D(t) = \frac{b}{\lambda}\left(\frac{t}{\lambda}\right)^{b-1} e^{-(t/\lambda)^b} \tag{S1.25}$$

$$U(t) = \lambda_u exp(-\lambda_u t) \tag{S1.26}$$

To capture the dynamics of cell cycle transitions in damaged daughter cells, we derive the pdf for the time taken by a cell to progress from node i to node $i+1(\text{mod } 3)$, which is formalized in Eq S1.27. The corresponding cdf is denoted as $\tilde{G}_i(t)$ (equation not shown). This transition time encapsulates two distinct scenarios: (A) After the walker arrives at the node i at time t', the cell successfully repairs treatment-induced double-strand breaks (DSBs), overcoming the checkpoint barrier to advance to the subsequent phase at time t. This also means that the waiting time imposed by the walker is reached, and at the same time, the link connecting to the next cell cycle phase is available; (B) After the walker arrives at the node i at time t', the cell encounters a temporary cell cycle arrest due to unresolved DSBs at node i at time t'. During this time, while the waiting time imposed by the walker is reached, the edges $n_d \leftarrow n_i$ and $n_{i+1(\text{mod } 3)} \leftarrow n_i$ are disabled until t, leaving the walker trapped at node i. However, if the edge $n_{i+1(\text{mod } 3)} \leftarrow n_i$ becomes active precisely at time t, the walker will be able to move to the next phase exactly at that moment.

$$\begin{aligned} &f\left(t-t'; n_{i+1(\text{mod } 3)} \leftarrow n_i \mid n_i \leftarrow n_{i-1}\right) = \\ &\quad \psi_i(t-t')\Big(1-\tilde{p}_{i+1(\text{mod } 3\ )\leftarrow i}(t-t')\Big) + \\ &\int_{t'}^{t} \psi_i(x-t')[(1-\tilde{p}_{d\leftarrow i})\mathrm{P_d}\{w > t-x\}] \times \tilde{p}_{i+1(\text{mod } 3)\leftarrow i}(x-t')\mathscr{D}(t-x)\mathrm{d}x \end{aligned} \tag{S1.27}$$

which also reads as

$$\begin{aligned} &f\left(\tau; n_{i+1(\text{mod } 3)} \leftarrow n_i \mid n_i \leftarrow n_{i-1}\right) = \\ &\quad \psi_i(\tau)\Big(1-\tilde{p}_{i+1(\text{mod } 3\ )\leftarrow i}(\tau)\Big) + \\ &\int_{0}^{\tau} \psi_i(r)[(1-\tilde{p}_{d\leftarrow i})\mathrm{P_d}\{w > \tau-r\}] \times \tilde{p}_{i+1(\text{mod } 3)\leftarrow i}(r)\mathscr{D}(\tau-r)\mathrm{d}r \end{aligned} \tag{S1.28}$$

$$\mathscr{D}(t) = \frac{1}{\langle D \rangle} \int_t^{\infty} D(\nu)\mathrm{d}\nu \tag{S1.29}$$

where $\tilde{p}_{d \leftarrow i}$ refers to the probability that a edge $d \leftarrow i$ is active, and is dependent on the $\langle U \rangle, \langle D \rangle$ (Eq S1.33). $\mathscr{D}(w)$ is the pdf of the waiting time w of the walker being trapped on node i before the edge $n_{i+1(\text{mod } 3)} \leftarrow n_i$ becomes available and after walker is ready to jump (Eq S1.29). $\mathrm{P_d}\{w > \tau - r\}$ represents the probability that the walker will be trapped in the edge $n_d \leftarrow n_i$ longer than $\tau - r$. $\tilde{p}_{i+1(\text{mod } 3) \leftarrow i}(r)$ is the probability that the edge is unavailable for transport at r time units since the walker arrived at node i, and is calculated by accounting for all possible on-off switches, that is Eq S1.30. It is easier to calculate the complement of $\tilde{p}_{i+1(\text{mod } 3) \leftarrow i}(x - t')$, that denotes the probability that the edge is up for transport at x since the walker arrived at node i at time t'.

$$\begin{aligned} \tilde{p}_{i+1(\text{mod } 3) \leftarrow i}(s) &= 1 - P\{\text{edge } i+1(\text{mod } 3) \leftarrow i \text{ is up at } s\} \\ &= 1 - \int_0^s D(r) \int_{s-r}^{\infty} U(\tau)\mathrm{d}\tau \, \mathrm{d}r \end{aligned} \tag{S1.30}$$

Conversely, the pdf for the transition time from node i to the death node d is described in Eq S1.31, which defines the scenario where a cell fails to repair DSBs and activates the apoptotic pathway at time t. The sole scenario considered here posits that upon the cell's arrival at node i at time t', if both the progression (edge $n_{i+1(\text{mod } 3)} \leftarrow n_i$) and the death pathways (edge $n_d \leftarrow n_i$) are blocked until time t, but the path to apoptosis is activated precisely at time t, the cell will proceed to cell death.

$$\begin{aligned} & f\left(t - t'; n_d \leftarrow n_i \mid n_i \leftarrow n_{i-1}\right) = \\ & \int_{t'}^{t} \psi_i(x - t')[\tilde{p}_{i+1(\text{mod } 3) \leftarrow i}(x - t')\mathrm{P}\{w > t - x\}] \times (1 - \tilde{p}_{d \leftarrow i})\mathscr{D}_d(t - x)\mathrm{d}x \end{aligned} \tag{S1.31}$$

which also reads as

$$\begin{aligned} & f\left(\tau; n_d \leftarrow n_i \mid n_i \leftarrow n_{i-1}\right) = \\ & \int_0^{\tau} \psi_i(r)[\tilde{p}_{i+1(\text{mod } 3) \leftarrow i}(r)\mathrm{P}\{w > \tau - r\}] \times (1 - \tilde{p}_{d \leftarrow i})\mathscr{D}_d(\tau - r)\mathrm{d}r \end{aligned} \tag{S1.32}$$

$$\tilde{p}_{d \leftarrow i} = \mathrm{P}\{\text{ edge } n_d \leftarrow n_i \text{ is active at a random time }\} = \frac{1/\lambda_u}{1/\lambda_u + \lambda_d \Gamma(1 + 1/b_d)} \tag{S1.33}$$

The density of resting time on node i (also known as transition density) satisfies the normalization condition, that is

$$\int_0^{\infty} f\left(\tau; n_{i+1(\text{mod } 3)} \leftarrow n_i \mid n_i \leftarrow n_{i-1}\right) + f\left(\tau; n_d \leftarrow n_i \mid n_i \leftarrow n_{i-1}\right) d\tau = 1, \tag{S1.34}$$

meaning that a jump will eventually occur since the out-degree in the underlying graph is positive. We use $\tilde{G}_i(t)$, the corresponding cdf of $f\left(\tau; n_{i+1(\text{mod } 3)} \leftarrow n_i \mid n_i \leftarrow n_{i-1}\right)$ to quantify the likelihood of a cell advancing from node i to node $i+1(\text{mod } 3)$ within the time interval t. Likewise, we use $\tilde{G}_d(t)$, the corresponding cdf of $f\left(\tau; n_d \leftarrow n_i \mid n_i \leftarrow n_{i-1}\right)$ to quantify the likelihood of a cell advancing from node i to node n_d within the time interval t. There is no correlation between $\tilde{G}_i(t)$ and $\tilde{G}_d(t)$, meaning that apoptosis and cell cycle progression are independent competing processes.

3 Simulation of model under treatment conditions

3.1 Residual time that cells would remain in their current phase post-exposure

In the main text, we categorize the cell population into three groups based on their state at the time of treatment and track how drug exposure differs between cells that were already mid-cycle when treatment began and those generated afterward. The figure C illustrates the three populations.

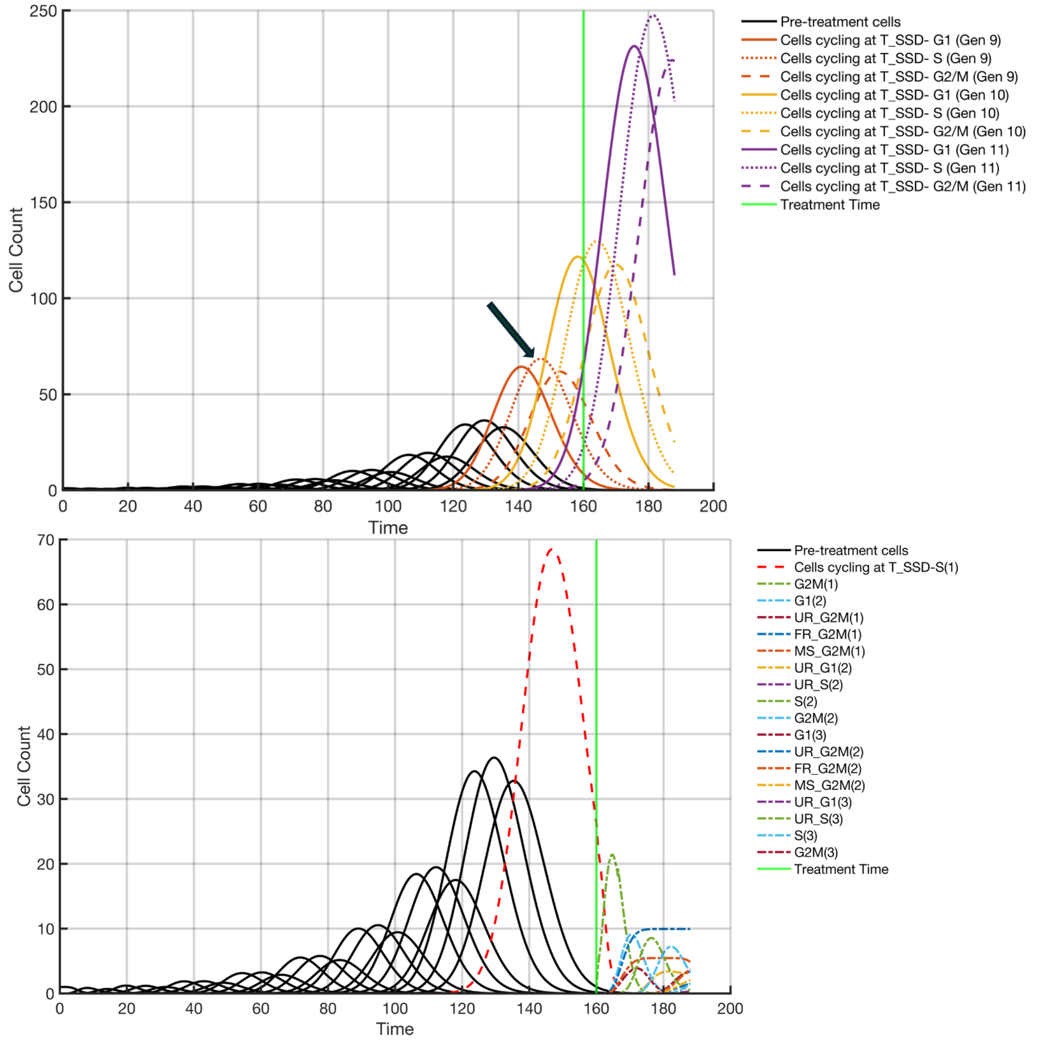

Figure C. Illustration of three cell types in treatment scenarios under G2/M phase drugs. The solid black lines in both the upper and lower panels represent cells that completed division before the start of treatment with only one ancestor cell at the start of simulation (time 0). The red dashed lines indicate cells that are actively cycling at time T_{SSD}, meaning their counts at T_{SSD} exceed a predefined threshold. Each of these actively cycling cells initiates a new branching process, with dynamics influenced by whether the drug targets the phase the cell is in. To illustrate cells generated after T_{SSD}, we focus on cells cycling in the S phase of the 9th generation since time 0 (highlighted by a black arrow). The post-T_{SSD} progeny (i.e., the cells generated after treatment begins) are shown as colorful dot-dashed lines in the lower panel. UR: unfaithful repair; FR: faithful repair; MS: mitotic slippage. The numbers in parentheses in the legend of the upper panel indicate the generation number counted from the start of the simulation (time 0). In the lower panel, the numbers in parentheses indicate the generation number counted since T_{SSD}. The top panel illustrates the cell population dynamics in the absence of treatment, while the bottom panel shows the dynamics under treatment.

After the introduction of G2/M and S phase-specific drugs, a subset of cancer cells may either avoid treatment-induced DNA damage by being outside the targeted cell cycle phases or, if targeted, temporarily maintain their current undamaged state before jumping to the next state, depending on transition probabilities. To simulate this behavior in our model, a critical step involves identifying these "cycling cells" and determining their phase status at the time of treatment.

The process of identifying cycling cells at the time of treatment (T_{SSD}) involves examining cell number trajectories, denoted as $M_{1,i}(t)$, for each cell cycle phase i to see which cells are actively progressing through their cycles at the onset of treatment. This involves establishing a threshold

criterion to evaluate the cell number trajectories $M_{1,i}(t)$, which represents the first row of the $M(t)$ matrix corresponding to the state i cell initiated by G1 cells at the treatment time T_{SSD}. We set a threshold, a cell count of 2, to identify active cycling status. Cells whose count at T_{SSD} exceeds this threshold are classified as actively cycling and are grouped into a designated set A.

Once the cycling cells are identified, we focus on estimating the residual time they will spend in their current phase post-treatment. This involves calculating the cdf of the total time elapsed from the initiation of the branching process at time 0 to the end of a specific division class (k_t), represented by the random variable T_{k_t}. The cdf of T_{k_t} is calculated as $\left[(\mathbf{G})^{*k_t}(\mathrm{t})\right]_{1,1+k_t}$. From this, we derive the distribution of residual time in the current division class, a random variable R_{k_t} with distribution function $\hat{G}_{k_t}$, which satisfies:

$$P(T_{k_t} <= T_{SSD}) * \hat{G}_{k_t} = \left[(\mathbf{G})^{*k_t}(\mathrm{t})\right]_{1,1+k_t} \tag{S1.35}$$

where $*$ represents convolution. Eq S1.35 allows us to isolate $\hat{G}_{k_t}$ via deconvolution.

Computing the residual time distribution enables us to characterize how cycling cells will continue to progress following treatment. Accordingly, cells within division class k_t initiate a new branching process governed by a modified transition matrix and a distinct diagonal distribution matrix that reflects the altered dynamics induced by treatment.

3.2 Modification of transition and diagonal distribution matrices to reflect treatment-induced dynamics

We define the post-treatment transition matrix $\hat{\mathbf{m}}_p$ and the diagonal distribution matrix $\hat{\mathbf{G}}_p$, for $p = \{1, 2, 3\}$, corresponding to cells that were actively cycling at the time of treatment. These matrices are phase-specific, determined by the cell's position in the cell cycle at T_{SSD}. To account for treatment effects, we also adapted the calculation of cell lineage propagation matrix $\mathbf{M}(t)$ in Eq S1.16 to a modified form. Specifically, cells cycling at T_{SSD} initiate a new branching process governed by the post-treatment transition matrix $\hat{\mathbf{m}}_p$ and diagonal distribution matrix $\hat{\mathbf{G}}_p$, which reflects treatment-altered progression dynamics. The corresponding cell lineage propagation matrix $\hat{\mathbf{M}}_p(t)$, defined in Eq S1.36, captures the altered dynamics of cells cycling at the time of treatment and those generated after T_{SSD}.

$$\hat{\mathbf{M}}_p(t) = \sum_{k=0}^{N_{sgen}^{D} n_{gen}^{t}} (\hat{\mathbf{G}}_p \hat{\mathbf{m}}_p)^{*k}(t) * [\mathbf{I} - \hat{\mathbf{G}}_p(t)] \tag{S1.36}$$

In which each diagonal component in the 3D matrix $\hat{\mathbf{G}}_p$ represents the cdf of age-dependent transition distribution post-treatment for each cell cycle state. N_{sgen}^{D} represents the number of states in one generation post-treatment of drug affecting phase D. n_{gen}^{t} represents the number of generations after T_{SSD}.

The expected number of cells in phase p post-treatment can be calculated as

$$E\left[\Phi_p(t)\right] = \sum_{k=1}^{3} N_k^{T_{SSD}} \sum_{g=1}^{N_{sgen}^{D} n_{gen}^{t}} \hat{M}_{k,g}(t), \text{if } g \in S_p \tag{S1.37}$$

where S_p denotes the set of states occurring in cell cycle phase p. For example, if the treatment is G2/M phase treatment, and the current cycling cells are in the G1 phase, then the transition matrix of the branching process starting from G1 phase cells at T_{SSD} ($\hat{\mathbf{m}}_\mathbf{1}$) up to two generations after exposure will be the following matrix.

where the column and row names are the cells states post-treatment. The number within the parentheses represents the number of generations post-treatment. G1_2 represents the state whose distribution dictates the residual time a G1 phase cell will remain in the current phase after treatment.

The distribution of the lifetime of apoptotic cells is assumed to be exponential, with an average of 5 hours, based on peak caspase activation in H460 cells following TRAIL stimulation and the typically rapid in vivo clearance of apoptotic cells by macrophages [9, 10].

	G1_2	S	G2/M	G1(2)	UR_G2/M	FR_G2/M	MS_G2/M	G1arrest(2)	Sarrest(2)	S(2)	G2/M(2)	Apop
G1_2	0	1	0	0	0	0	0	0	0	0	0	0
S	0	0	1	0	0	0	0	0	0	0	0	0
G2/M	0	0	0	$2(1-q_1-q_2-q_3)$	q_1	q_2	q_3	0	0	0	0	0
G1(2)	0	0	0	0	0	0	0	0	0	1	0	0
UR_G2/M	0	0	0	0	0	0	0	$2(1-m_{d,UR,G2/M})$	0	0	0	$m_{d,UR,G2/M}$
FR_G2/M	0	0	0	$2(1-m_{d,FR,G2/M})$	0	0	0	0	0	0	0	$m_{d,FR,G2/M}$
MS_G2/M	0	0	0	0	0	0	0	$(1-m_{d,MS,G2/M})$	0	0	0	$m_{d,MS,G2/M}$
G1arrest(2)	0	0	0	0	0	0	0	0	$1-m_{d,G1arrest}-q4$	q_4	0	$m_{d,G1arrest}$
Sarrest	0	0	0	0	0	0	0	0	0	0	$1-m_{d,Sarrest}$	$m_{d,Sarrest}$
S(2)	0	0	0	0	0	0	0	0	0	0	1	0
G2/M(2)	0	0	0	0	0	0	0	0	0	0	0	0
Apop	0	0	0	0	0	0	0	0	0	0	0	0

282 ## 3.3 Cell cycle simulation of cotreatment with paclitaxel and gemcitabine

283 The simulation of the cotreatment is implemented by modifying the post-treatment transition matrix
284 $\hat{m}_p$ in Eq S1.36. Specifically, we construct an integrated transition matrix that combines the effects
285 of both paclitaxel and gemcitabine. This matrix accounts for the joint impact of the two drugs on cell state transitions following treatment. The integrated transition matrix is shown below.

	G1_2	G1_block	S	UR_S	FR_S	G2M	UR_G2M	FR_G2M	MS_G2M	G1(2)	G1_block(2)	UR_G1(2)	S(2)	UR_S(2)	FR_S(2)	G2M(2)	UR_G2M(2)	FR_G2M(2)	MS_G2M(2)	Apop
G1_2		$q_{1,S}$	$1-q_{1,S}$																	
G1_block			$1-m_{d,G1block}$																	$m_{d,G1block}$
S				$q_{2,S}$	$q_{3,S}$	$1-q_{2,S}-q_{3,S}$														
UR_S							$1-m_{d,UR,S}$													$m_{d,UR,S}$
FR_S						$1-m_{d,FR,S}$														$m_{d,FR,S}$
G2M							$q_{1,G2M}$	$q_{2,G2M}$	$q_{3,G2M}$	$2(1-q_{1,G2M}-q_{2,G2M}-q_{3,G2M})$										
UR_G2M												$2(1-m_{d,UR,G2M})$								$m_{d,UR,G2M}$
FR_G2M										$2(1-m_{d,FR,G2M})$										$m_{d,FR,G2M}$
MS_G2M												$(1-m_{d,MS,G2M})$								$m_{d,MS,G2M}$
G1(2)											$q_{1,S}$		$1-q_{1,S}$							
G1_block(2)													$1-m_{d,G1block}$							$m_{d,G1block}$
UR_G1(2)													$q_{4,G2M}$	$1-m_{d,UR,G1}-q_{4,G2M}$						$m_{d,UR,G1}$
S(2)														$q_{2,S}$	$q_{3,S}$	$1-q_{2,S}-q_{3,S}$				
UR_S(2)																	$1-m_{d,UR,S}$			$m_{d,UR,G1}$
FR_S(2)																$1-m_{d,FR,S}$				$m_{d,FR,S}$
G2M(2)																	$q_{1,G2M}$	$q_{2,G2M}$	$q_{3,G2M}$	
UR_G2M(2)																				
FR_G2M(2)																				
MS_G2M(2)																				
Apop																				

287 # 4 Derivation of the steady state

288 In our study, the cell cycle is divided into three phases. Thus, the cdf of the duration of phase i
289 $(i = G1, S, G2/M)$ to be $G_i(t)$, that is

$$G_i(x) = \text{ prob } \{\text{ duration of phase } i \text{ cell } \leqslant x\} \tag{S1.38}$$

290 The cdf of the duration of the entire cell cycle is denoted by $G(t)$. We assume that the durations of
291 the individual cell phases are independently and identically distributed. Likewise, the total duration
292 of the cell cycle for each cell also follows an independent and identical distribution. A given cell in
293 the nth generation is present in phase G1 at time t if and only if the sum of (n-1) lifetimes does not
294 exceed t while the sum of $(n-1)$ lifetimes plus the duration of G1 phase is not reached within t.
295 Thus, the probability that a nth generation cell is in phase G1 at time t is

$$G^{*(n)}(t) - G^{*(n)} * G_1(t) \tag{S1.39}$$

296 where $G^{*n}(t)$ denotes n-fold convolution of function $G(t)$ itself ie., $G^{*n}(t) = G * G * \ldots * G(t)$. The
297 convolution is defined to be a Lebesgue-Stieltjes integral such that $A(t) * B(t) = \int_0^t A(t-\tau) d_\tau B(\tau)$
298 when $A(t)$ and $B(t)$ are two right continuous functions with a locally bounded variation on $[0, \infty)$.
299 $G^{*(n)} * G_1$ is the distribution function for the sum of n independent cell cycle times from generations
300 $0, 1, \ldots, n-1$ plus one phase G1 duration sampled from Eq S1.38. Likewise, the probability that a
301 nth generation cell is in phase S at time t is

$$G^{*(n)}(t) * G_1(t) - G^{*(n)} * G_1(t) * G_2(t) \tag{S1.40}$$

302 The probability that an nth generation cell is in phase G2/M at time t is

$$G^{*(n)} * G_1 * G_2(t) - G^{*(n+1)}(t) \tag{S1.41}$$

303 Then the expected number of cells in phase G1 at time t is given by

$$E(N_1(t)) = K \sum_{n=0}^{\infty} 2^n \{G^{*n}(t) - G^{*n} * G_1(t)\} \tag{S1.42}$$

K represents the initial total number of ancestor cells.

The expected number of cells in phase S at time t is given by

$$E(N_2(t)) = K \sum_{n=0}^{\infty} 2^n \left\{G^{*n} * G_1(t) - G^{*n} * G_1 * G_2(t)\right\} \tag{S1.43}$$

The expected number of cells in phases G2/M at time t is given by

$$E(N_3(t)) = K \sum_{n=0}^{\infty} 2^n \left\{G^{*n} * G_1 * G_2(t) - G^{*(n+1)}(t)\right\} \tag{S1.44}$$

The expected number of cells at time t is given by

$$E(N(t)) = K \sum_{n=0}^{\infty} 2^n \left\{G^{*n}(t) - G^{*(n+1)}(t)\right\} \tag{S1.45}$$

The population growth rate in the exponential growth phase is denoted as γ, which is also the Malthusian parameter defined as the positive root of the equation

$$m \int_0^{\infty} \mathrm{e}^{-\gamma x} \, \mathrm{d}G(x) = m \cdot \mathcal{L}\{G(x)\} = 1 \tag{S1.46}$$

where m denotes the number of offsprings produced by each cell. $\mathcal{L}\{\cdot\}$ represents the Laplace transform. Using this growth rate γ, the asymptotic population distribution as $t \to \infty$ is expressed as

$$\begin{aligned} p_1^* &= \frac{\int_0^{\infty} e^{-\gamma \ell} dE\left(N_1(\ell)\right)}{\int_0^{\infty} e^{-\gamma \ell} dE(N(\ell))} \\ p_2^* &= \frac{\int_0^{\infty} e^{-\gamma \ell} dE\left(N_2(\ell)\right)}{\int_0^{\infty} e^{-\gamma \ell} dE(N(\ell))} \\ p_3^* &= \frac{\int_0^{\infty} e^{-\gamma \ell} dE\left(N_3(\ell)\right)}{\int_0^{\infty} e^{-\gamma \ell} dE(N(\ell))} \end{aligned} \tag{S1.47}$$

From the expressions for $E\left(N_1(t)\right)$, $E\left(N_2(t)\right)$, $E\left(N_3(t)\right)$, and $E\left(N(t)\right)$ we can get

$$\begin{aligned} p_1^* &= \frac{\int_0^{\infty} e^{-\gamma \ell} d\left[K \sum_{n=0}^{\infty} 2^n \left(G^{*n}(\ell) - G^{*n} * G_1(\ell)\right)\right]}{\int_0^{\infty} e^{-\gamma \ell} d\left[K \sum_{n=0}^{\infty} 2^n \left(G^{*n}(\ell) - G^{*(n+1)}(\ell)\right)\right]} \\ p_2^* &= \frac{\int_0^{\infty} e^{-\gamma \ell} d\left[K \sum_{n=0}^{\infty} 2^n \left(G^{*n} * G_1(\ell) - G^{*n} * G_1 * G_2(\ell)\right)\right]}{\int_0^{\infty} e^{-\gamma \ell} d\left[K \sum_{n=0}^{\infty} 2^n \left(G^{*n}(\ell) - G^{*(n+1)}(\ell)\right)\right]} \\ p_3^* &= \frac{\int_0^{\infty} e^{-\gamma \ell} d\left[K \sum_{n=0}^{\infty} 2^n \left(G^{*n} * G_1 * G_2(\ell) - G^{*(n+1)}(\ell)\right)\right]}{\int_0^{\infty} e^{-\gamma \ell} d\left[K \sum_{n=0}^{\infty} 2^n \left(G^{*n}(\ell) - G^{*(n+1)}(\ell)\right)\right]} \end{aligned} \tag{S1.48}$$

We can factor out K and the sum over n from both the numerator and denominator, which gives us:

$$\begin{aligned} p_1^* &= \frac{\sum_{n=0}^{\infty} 2^n \int_0^{\infty} e^{-\gamma \ell} d\left(G^{*n}(\ell) - G^{*n} * G_1(\ell)\right)}{\sum_{n=0}^{\infty} 2^n \int_0^{\infty} e^{-\gamma \ell} d\left(G^{*n}(\ell) - G^{*(n+1)}(\ell)\right)} \\ p_2^* &= \frac{\sum_{n=0}^{\infty} 2^n \int_0^{\infty} e^{-\gamma \ell} d\left(G^{*n} * G_1(\ell) - G^{*n} * G_1 * G_2(\ell)\right)}{\sum_{n=0}^{\infty} 2^n \int_0^{\infty} e^{-\gamma \ell} d\left(G^{*n}(\ell) - G^{*(n+1)}(\ell)\right)} \\ p_3^* &= \frac{\sum_{n=0}^{\infty} 2^n \int_0^{\infty} e^{-\gamma \ell} d\left(G^{*n} * G_1 * G_2(\ell) - G^{*(n+1)}(\ell)\right)}{\sum_{n=0}^{\infty} 2^n \int_0^{\infty} e^{-\gamma \ell} d\left(G^{*n}(\ell) - G^{*(n+1)}(\ell)\right)} \end{aligned} \tag{S1.49}$$

Since the Laplace transform of a cdf $G(t)$ is defined as $\mathcal{L}\{G(t)\} = \int_0^{\infty} e^{-\gamma \ell} dG(\ell)$, applying this to the functions S1.49, we have

$$
\begin{aligned}
p_1^* &= \frac{\sum_{n=0}^{\infty} 2^n \left[\mathcal{L}\left\{G^{*n}(t)\right\} - \mathcal{L}\left\{G^{*n} * G_1(t)\right\}\right]}{\sum_{n=0}^{\infty} 2^n \left[\mathcal{L}\left\{G^{*n}(t)\right\} - \mathcal{L}\left\{G^{*(n+1)}(t)\right\}\right]} \\
p_2^* &= \frac{\sum_{n=0}^{\infty} 2^n \left[\mathcal{L}\left\{G^{*n} * G_1(t)\right\} - \mathcal{L}\left\{G^{*n} * G_1 * G_2(t)\right\}\right]}{\sum_{n=0}^{\infty} 2^n \left[\mathcal{L}\left\{G^{*n}(t)\right\} - \mathcal{L}\left\{G^{*(n+1)}(t)\right\}\right]} \\
p_3^* &= \frac{\sum_{n=0}^{\infty} 2^n \left[\mathcal{L}\left\{G^{*n} * G_1 * G_2(t)\right\} - \mathcal{L}\left\{G^{*(n+1)}(t)\right\}\right]}{\sum_{n=0}^{\infty} 2^n \left[\mathcal{L}\left\{G^{*n}(t)\right\} - \mathcal{L}\left\{G^{*(n+1)}(t)\right\}\right]}
\end{aligned} \tag{S1.50}
$$

Given that $G^{*n}(t)$ is the n-fold convolution of $G(t)$ with itself, the Laplace transform of $G^{*n}(t)$ is $(\mathcal{L}\{G(t)\})^n$. Using this, the above expressions (Eq S1.50) simplify to

$$
\begin{aligned}
p_1^* &= \frac{\sum_{n=0}^{\infty} 2^n \left[\mathcal{L}\{G(t)\}^n - \mathcal{L}\{G(t)\}^n \cdot \mathcal{L}\left\{G_1(t)\right\}\right]}{\sum_{n=0}^{\infty} 2^n \left[\mathcal{L}\{G(t)\}^n - \mathcal{L}\{G(t)\}^{n+1}\right]} \\
p_2^* &= \frac{\sum_{n=0}^{\infty} 2^n \left[\mathcal{L}\{G(t)\}^n \cdot \mathcal{L}\left\{G_1(t)\right\} - \mathcal{L}\{G(t)\}^n \cdot \mathcal{L}\left\{G_1(t)\right\} \cdot \mathcal{L}\left\{G_2(t)\right\}\right]}{\sum_{n=0}^{\infty} 2^n \left[\mathcal{L}\{G(t)\}^n - \mathcal{L}\{G(t)\}^{n+1}\right]} \\
p_3^* &= \frac{\sum_{n=0}^{\infty} 2^n \left[\mathcal{L}\{G(t)\}^n \cdot \mathcal{L}\left\{G_1(t)\right\} \cdot \mathcal{L}\left\{G_2(t)\right\} - \mathcal{L}\{G(t)\}^{(n+1)}\right]}{\sum_{n=0}^{\infty} 2^n \left[\mathcal{L}\{G(t)\}^n - \mathcal{L}\{G(t)\}^{n+1}\right]}
\end{aligned} \tag{S1.51}
$$

We know from the Malthusian parameter γ that $m\int_0^{\infty} e^{-\gamma x} dG(x) = m \cdot \mathcal{L}\{G(x)\} = 1$. Thus, $\mathcal{L}\{G(t)\} = \frac{1}{m}$. Substituting this into the expression and factoring out $\left(\frac{1}{m}\right)^n$, we can get

$$
\begin{aligned}
p_1^* &= \frac{\sum_{n=0}^{\infty} \left(\frac{2}{m}\right)^n \left[1 - \mathcal{L}\left\{G_1(t)\right\}\right]}{\sum_{n=0}^{\infty} \left(\frac{2}{m}\right)^n \left[1 - \frac{1}{m}\right]} \\
p_1^* &= \frac{\sum_{n=0}^{\infty} \left(\frac{2}{m}\right)^n \left[\mathcal{L}\left\{G_1(t)\right\} - \mathcal{L}\left\{G_1(t)\right\} \mathcal{L}\left\{G_2(t)\right\}\right]}{\sum_{n=0}^{\infty} \left(\frac{2}{m}\right)^n \left[1 - \frac{1}{m}\right]} \\
p_3^* &= \frac{\sum_{n=0}^{\infty} \left(\frac{2}{m}\right)^n \left[\mathcal{L}\left\{G_1(t)\right\} \mathcal{L}\left\{G_2(t)\right\} - \mathcal{L}\left\{G(t)\right\}\right]}{\sum_{n=0}^{\infty} \left(\frac{2}{m}\right)^n \left[1 - \frac{1}{m}\right]}
\end{aligned} \tag{S1.52}
$$

These can be further simplified to

$$
\begin{aligned}
p_1^* &= \frac{m[1 - \mathcal{L}\left\{G_1(t)\right\}]}{m-1} \\
p_2^* &= \frac{m[\mathcal{L}\left\{G_1(t)\right\} - \mathcal{L}\left\{G_1(t)\right\} \mathcal{L}\left\{G_2(t)\right\}]}{m-1} \\
p_3^* &= \frac{m[\mathcal{L}\left\{G_1(t)\right\} \mathcal{L}\left\{G_2(t)\right\} - \mathcal{L}\left\{G(t)\right\}]}{m-1}
\end{aligned} \tag{S1.53}
$$

References

[1] Tom Serge Weber, Irene Jaehnert, Christian Schichor, Michal Or-Guil, and Jorge Carneiro. Quantifying the Length and Variance of the Eukaryotic Cell Cycle Phases by a Stochastic Model and Dual Nucleoside Pulse Labelling. *PLoS Comput Biol*, 10(7):e1003616, July 2014.

[2] Marek Kimmel and David E. Axelrod. *Branching Processes in Biology.* Springer, New York, NY, USA, 2015.

[3] Biao Li, Amanda Sierra, Juan Jose Deudero, Fatih Semerci, Andrew Laitman, Marek Kimmel, and Mirjana Maletic-Savatic. Multitype bellman-harris branching model provides biological predictors of early stages of adult hippocampal neurogenesis. *BMC systems biology*, 11:1–16, 2017.

[4] Theodore Edward Harris. *The theory of branching processes*, volume 6. Springer, 1963.

[5] Richard Cowan. Branching Process Results in Terms of Moments of the Generation-Time Distribution. *Biometrics*, 41(3):681–689.

[6] M. S. Ridout, D. J. Cole, B. J. T. Morgan, L. J. Byrne, and M. F. Tuite. New approximations to the Malthusian parameter. *Biometrics*, 62(4):1216–1223.

[7] Julien Petit, Renaud Lambiotte, and Timoteo Carletti. Classes of random walks on temporal networks with competing timescales. *Applied Network Science*, 4(1):1–20.

[8] Julien Petit, Martin Gueuning, Timoteo Carletti, Ben Lauwens, and Renaud Lambiotte. Random walk on temporal networks with lasting edges. *Phys. Rev. E*, 98(5):052307, November 2018.

[9] Zbigniew Darzynkiewicz, Xun Li, and Elzbieta Bedner. Use of flow and laser-scanning cytometry in analysis of cell death. In Zbigniew Darzynkiewicz and Juan C. Reed, editors, *Methods in Cell Biology*, volume 66, pages 69–109. Academic Press, 2001.

[10] L. Danish, D. Imig, F. Allgöwer, P. Scheurich, and N. Pollak. Bcl-2-mediated control of TRAIL-induced apoptotic response in the non-small lung cancer cell line NCI-H460 is effective at late caspase processing steps. *PLoS ONE*, 13(6):e0198203, 2018.
