# Supplementary material for: Probabilistic modeling of cell cycle dynamics in response to cell cycle targeting chemotherapy drugs to guide treatment strategies
Source: PLoS Comput Biol. 2025 Dec 16;21(12):e1013790. doi: 10.1371/journal.pcbi.1013790 (PMC12707676; doi:10.1371/journal.pcbi.1013790)
Supplement: S2 File — (PDF) [file pcbi.1013790.s002.pdf]

Probabilistic modeling of cell cycle dynamics in response to cell cycle targeting chemotherapy drugs to guide treatment strategies

Chenhui Ma[1,*], Evren Gurkan-Cavusoglu[1]

[1]Department of Electrical, Computer and Systems Engineering, Case Western Reserve University, Cleveland, Ohio, United States of America

[*]Corresponding author: cxm590@case.edu

Supplementary Information S2 File: Sensitivity analysis of the treatment model and identifiability analysis of the model parameters

---

1 Prior predictive checks

We used uniform priors for all model parameters, meaning that all parameter values within the specified ranges are considered equally plausible. This choice reflects the lack of empirical data to inform prior distributions. To evaluate the appropriateness of these priors, we performed prior predictive checks by generating synthetic data from the prior distributions and assessing whether they produce biologically plausible outputs. The procedure for conducting a prior predictive check for each model is as follows:

We first draw N parameter sets from the prior distribution $q(\boldsymbol{\Theta})$ as shown in Figures D–F:

$$\boldsymbol{\Theta}^{\text{sim},n} \sim q(\boldsymbol{\Theta}) \quad n = 1, \ldots, N \tag{S2.1}$$

In the case of a uniform distribution, the parameter sample matrix $\boldsymbol{\Theta}_i^{\text{sim}} \in \mathbb{R}^{N \times N_{\text{para},i}}$ is generated for each model i, where N is the number of parameter sets and $N_{\text{para},i}$ is the number of parameters in model i. The matrix $\boldsymbol{\Theta}_i^{\text{sim}}$ is computed as:

$$\boldsymbol{\Theta}_i^{\text{sim}} = \mathbf{u}_{\text{lb},i} + \texttt{sobolset}_i \cdot (\mathbf{u}_{\text{ub},i} - \mathbf{u}_{\text{lb},i}) \tag{S2.2}$$

where $\texttt{sobolset}_i$ is a matrix of quasi-random numbers generated using MATLAB's `sobolset` function. $\mathbf{u}_{\text{lb},i}$ and $\mathbf{u}_{\text{ub},i}$ represent lower bounds and upper bounds of parameters in model i.

We then simulate data from the treated model using these parameter sets:

$$y^{\text{sim},n} \sim p(y \mid \boldsymbol{\Theta}^{\text{sim},n}) \quad n = 1, \ldots, N \tag{S2.3}$$

We then assess the appropriateness of the prior distribution by comparing the simulated outputs $y^{\text{sim},n}$ with the observed data. The results are shown in Figure A, which demonstrates that the chosen parameter ranges are sufficiently broad: the observed data lie within the 95% credible intervals (i.e., between the 2.5th and 97.5th percentiles, $[q_{0.025}, q_{0.975}]$) of the prior predictive simulations.

2 Identifiability analysis of the model parameters after model fitting

The posterior distributions of parameters in baseline models are shown in Fig B-C. The posterior distributions of parameters in treatment models are shown in Fig D-F. These distributions encapsulate the uncertainty and variability inherent in the parameter estimates following the Bayesian framework applied in both baseline models and models with treatment. The width of the distributions reflects the level of certainty in parameter estimates, with narrower distributions indicating more precise estimations.

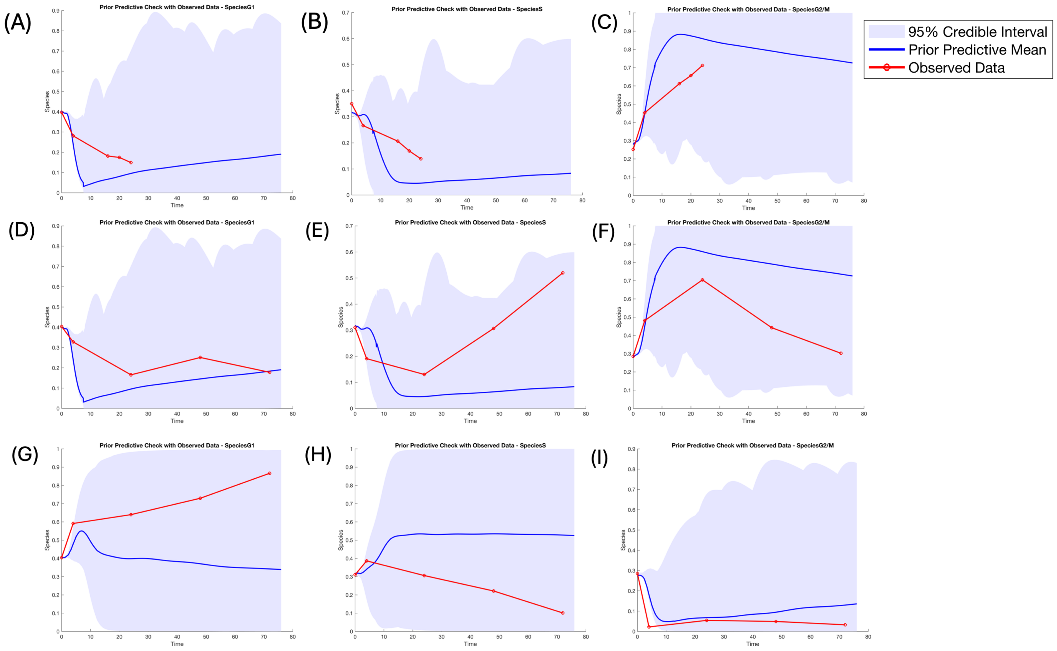

Fig A. Prior predictive check with observed data (A)-(C) docetaxel model; (D)-(F) paclitaxel model; (G)-(I) gemcitabine model.

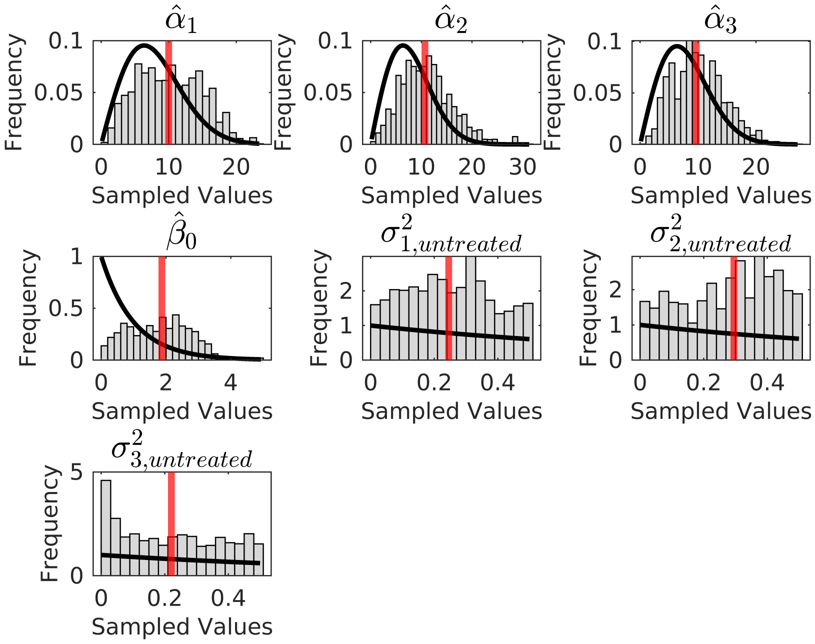

Fig B. Posterior distributions of parameters in baseline model 1. Overlayed with their respective prior distributions (black lines), histograms display the frequency distributions of MCMC sampled parameter values for $\hat{\alpha}_1, \hat{\alpha}_2, \hat{\alpha}_3, \hat{\beta}_0$, $\sigma^2_{1,\text{ untreated}}$, $\sigma^2_{2,\text{ untreated}}$, and $\sigma^2_{3,\text{ untreated}}$ from the posterior analysis. The vertical red line represents the median of the sampled values. The y axis represents the normalized frequency.

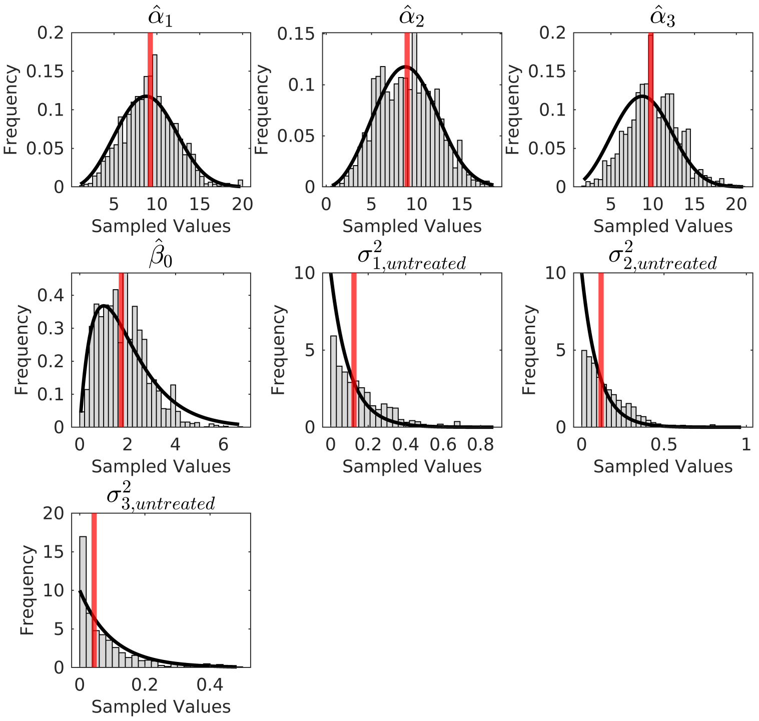

Fig C. Posterior distributions of parameters in baseline model 2 and baseline model 3. Overlayed with their respective prior distributions (black lines), histograms display the frequency distributions of MCMC sampled parameter values for $\hat{\alpha}_1, \hat{\alpha}_2, \hat{\alpha}_3, \hat{\beta}_0$, $\sigma^2_{1,\text{ untreated}}$, $\sigma^2_{2,\text{ untreated}}$, and $\sigma^2_{3,\text{ untreated}}$ from the posterior analysis. The vertical red line represents the median of the sampled values. The y axis represents the normalized frequency.

3 Sobol global analysis

The Sobol global sensitivity analysis was used to assess the impact of joint interactions among input parameters on the model outputs. First-order and total-order Sobol indices were calculated for the selected parameters. The first-order Sobol index measures the fraction of total variance that is attributed to the variations in each individual input parameter. The total-order Sobol index quantifies the fraction of the overall response variance that can be attributed to any joint parameter variations that include variations of the concerned parameter. The approximations of the first-order and total-order Sobol index are detailed in [1].

The first order and total order Sobol index of each parameter for both the G2/M phase model and the S phase model, calculated for the G2/M phase percentage at the $4.00, 24.00, 48.00$, and 72.00 hours after treatment initiation, are shown in Fig G and Fig H respectively. Fig G suggests that transition probabilities have a greater impact on model outputs at 24th hour posttreatment, while Fig H suggests that model outputs at the 4th hour posttreatment are more sensitive to transition probabilities. In Fig G, the parameters of probability distributions that represent the treatment-induced cell cycle protraction have a higher Sobol index when G2/M phase percentage at the $24.00, 48.00$, and 72.00 hours after treatment initiation is used as outputs, which is consistent with findings in Fig H. Furthermore, both Fig G and Fig H highlight the pronounced impact of the scale parameters associated with the unfaithful repair activated by drugs targeting the G2/M and S phases on the model's predictions. This influence is particularly pronounced around the 72-hour mark post-treatment. These parameters emerge as pivotal in modulating the model's output and could be potential targets for therapeutic intervention. The analysis in both Fig G and Fig H also indicates that the model's sensitivity to specific parameters can evolve over time, reflecting the complex biological processes in responses to pharmacological interventions at play.

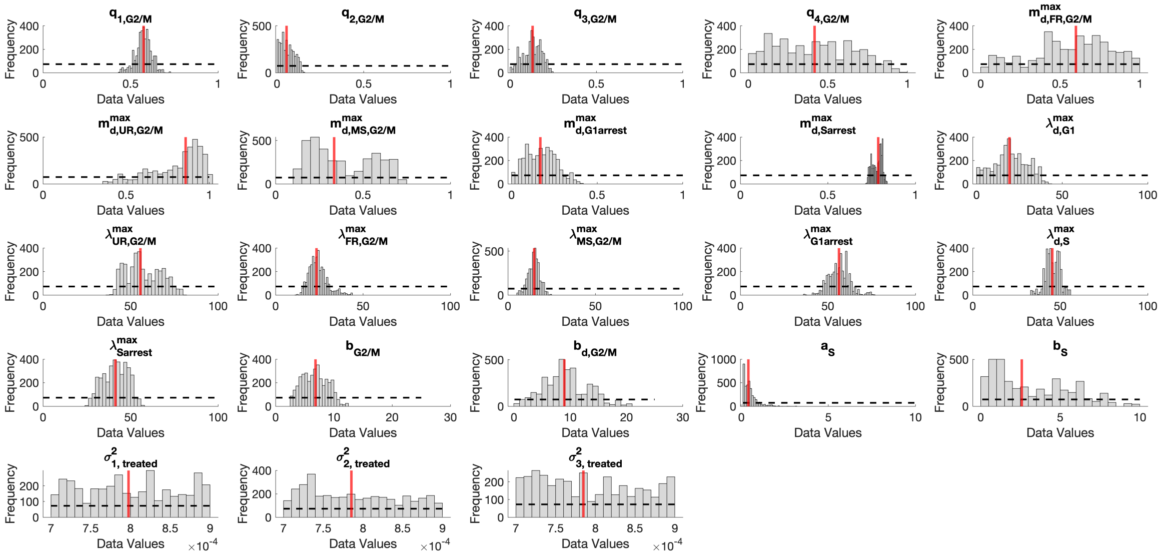

Fig D. Histograms of posterior distributions for G2/M-phase drug (docetaxel) model (model 1) parameters. With the overlay of the uniform prior distributions (black dashed line), these histograms illustrate the frequency of sampled values for transition probabilities ($q_{1,G2/M}$, $q_{2,G2/M}$, $q_{2,G2/M}$, $q_{3,G2/M}$, $q_{4,G2/M}$), maximum death rates ($m^{\max}_{d,FR,G2/M}$, $m^{\max}_{d,UR,G2/M}$, $m^{\max}_{d,MS,G2/M}$, and $m^{\max}_{d,G1arrest}$), variance of measurement noise in MCMC framework ($\sigma^2_{1,\text{ treated}}$,$\sigma^2_{2,\text{ treated}}$,$\sigma^2_{3,\text{ treated}}$), and parameters describing cell state transition time distributions. The vertical red line represents the median of the sampled values. The posterior distributions of the sampled parameters vary, with most parameters showing narrow, unimodal distributions. $q_{4,G2/M}$, $m^{\max}_{d,MS,G2/M}$ and the shape parameters $b_{d,G2/M}$ and b_S exhibit wider spreads and multimodal characteristics, suggesting higher uncertainty or variability. These broader, multimodal distributions indicate that additional data may be needed to better constrain these parameters and reduce estimation uncertainty.

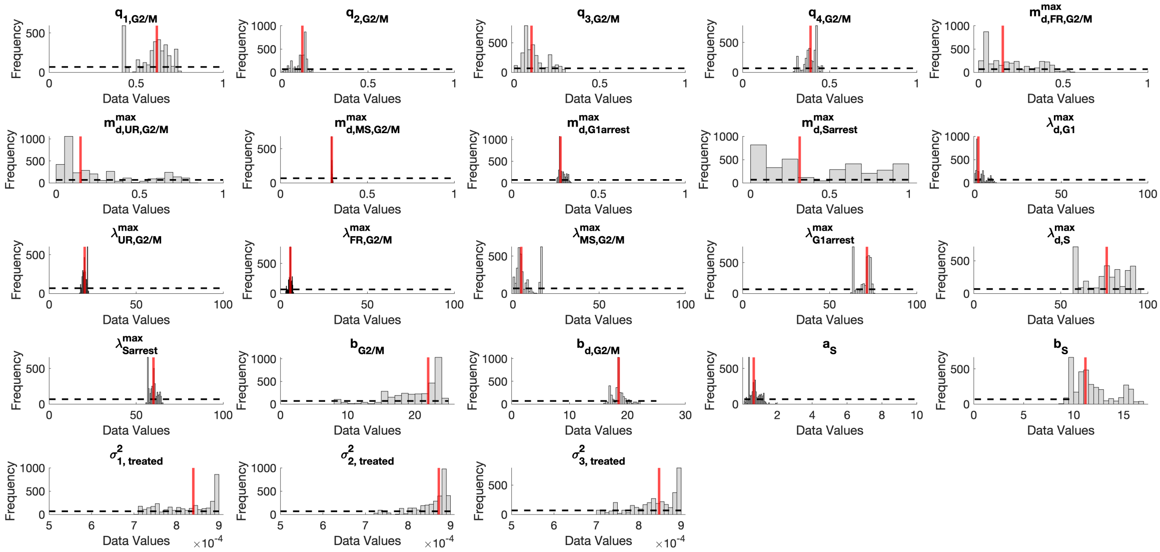

Fig E. Histograms of posterior distributions for G2/M-phase drug (paclitaxel) model (model 2) parameters. With the overlay of the uniform prior distributions (black dashed line), these histograms illustrate the frequency of sampled values for transition probabilities ($q_{1,G2/M}$, $q_{2,G2/M}$, $q_{2,G2/M}$, $q_{3,G2/M}$, $q_{4,G2/M}$), maximum death rates ($m^{\max}_{d,FR,G2/M}$, $m^{\max}_{d,UR,G2/M}$, $m^{\max}_{d,MS,G2/M}$, and $m^{\max}_{d,G1arrest}$), variance of measurement noise in MCMC framework ($\sigma^2_{1,\text{ treated}}$, $\sigma^2_{2,\text{ treated}}$, $\sigma^2_{3,\text{ treated}}$), and parameters describing cell state transition time distributions. The vertical red line represents the median of the sampled values. The histograms for G2/M phase drug model parameters exhibit varying degrees of spread and modality, with narrow spreads and unimodal distributions indicating good identifiability for most parameters. Conversely, wider spreads and potential multimodality in others (such as $q_{1,G2/M}$, $m^{\max}_{d,Sarrest}$, $\lambda^{max}_{MS,G2/M}$, $\lambda^{max}_{G1arrest}$, $\lambda^{max}_{d,S}$, and b_S) suggest higher uncertainty and the need for additional data to refine estimations.

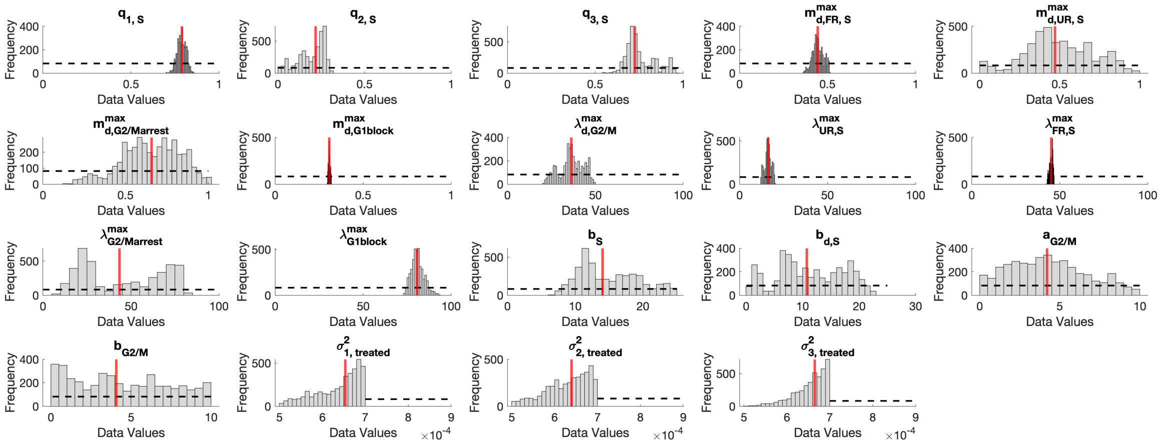

Fig F. Histograms of posterior distributions for S-phase drug (gemcitabine) model (model 3) parameters. With the overlay of uniform prior distributions (black dashed line), these histograms illustrate the frequency of sampled values for a set of model parameters. The vertical red line represents the median of the sampled values. The histograms for G2/M phase drug model parameters exhibit varying degrees of spread and modality, with narrow spreads and unimodal distributions indicating good identifiability for some parameters (such as $q_{1,S}$ through $q_{3,S}$, $m^{\max}_{d,FR,S}$, $m^{\max}_{d,G1block}$, $\lambda^{\max}_{d,G2/M}$, $\lambda^{\max}_{UR,S}$, $\lambda^{\max}_{FR,S}$, $\lambda^{\max}_{G1block}$). In contrast, wider spreads and potential multimodality in others (such as $m^{\max}_{d,UR,S}$, $m^{\max}_{d,G2/Marrest}$, $\lambda^{\max}_{G2/Marrest}$, b_S, $b_{d,S}$, $a_{G2/M}$, and $b_{G2/M}$) suggest higher uncertainty and the need for additional data to refine estimations.

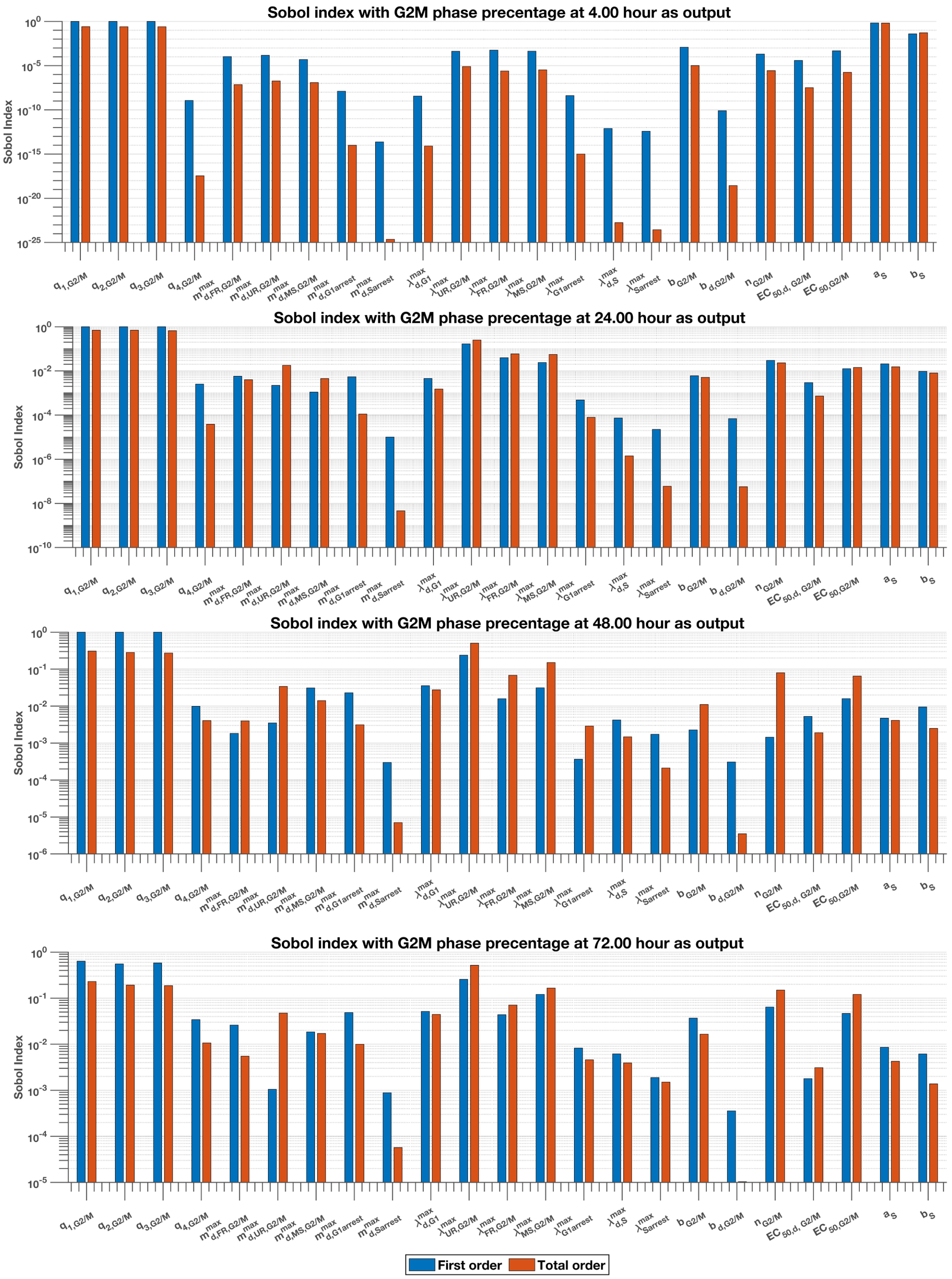

Fig G. Sensitivity analysis of the G2/M phase drug model at various time points posttreatment. The graphs display the Sobol indices for model parameters, with blue bars representing first-order effects and red bars representing total-order effects. The sensitivity indices are calculated for G2/M phase cell percentage as the output at $4.00, 24.00, 48.00,$ and 72.00 hours after treatment initiation. The y-axis is logarithmically scaled to capture the wide range of index values.

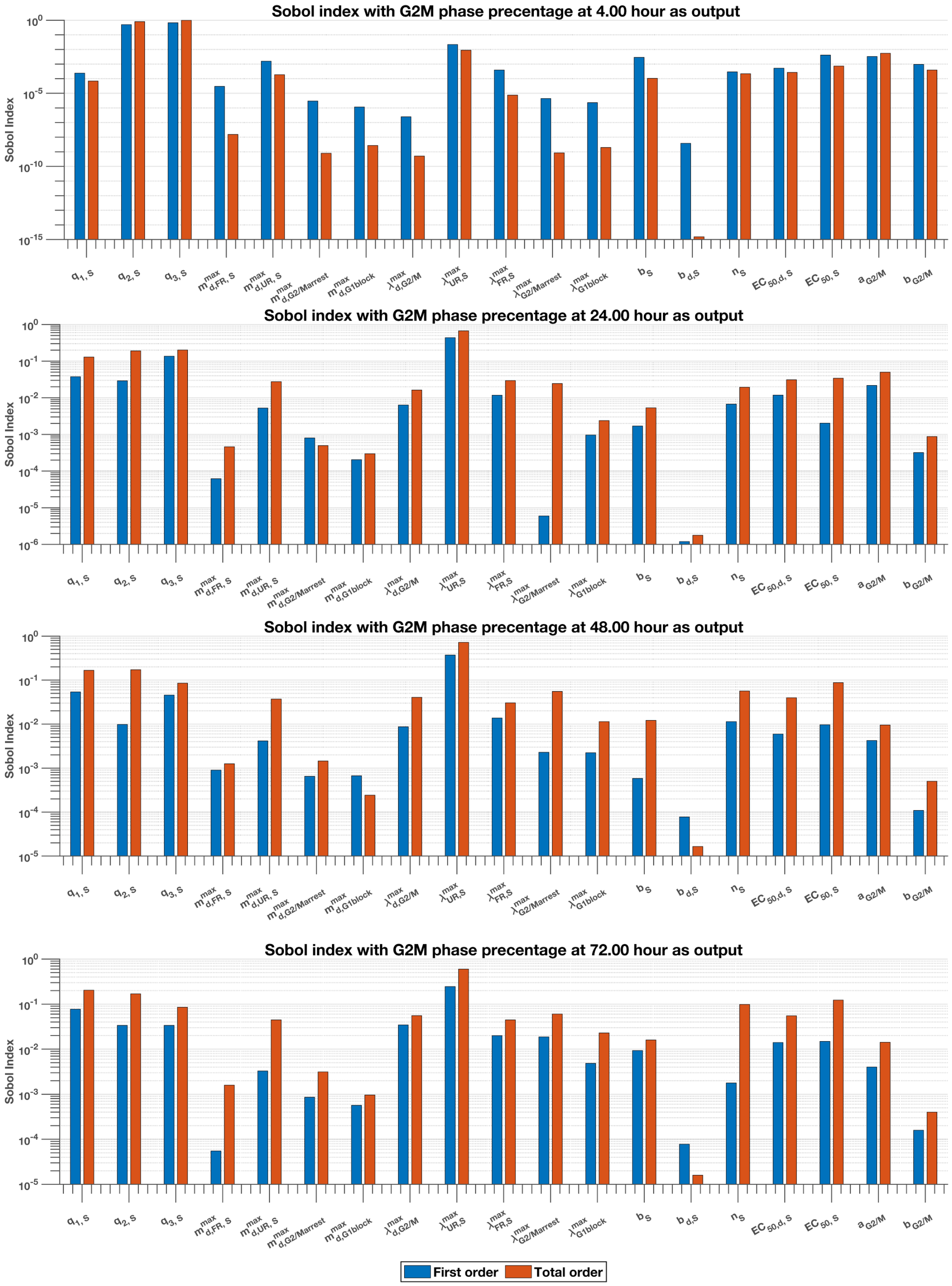

Fig H. Sensitivity analysis of the S phase drug model at various time points posttreatment. The graphs display the Sobol indices for model parameters, with blue bars representing first-order effects and red bars representing total-order effects. The sensitivity indices are calculated for G2/M phase cell percentage as the output at $4.00, 24.00, 48.00$, and 72.00 hours after treatment initiation. The y-axis is logarithmically scaled to capture the wide range of index values.

References

[1] Andrea Saltelli, Paola Annoni, Ivano Azzini, Francesca Campolongo, Marco Ratto, and Stefano Tarantola. Variance based sensitivity analysis of model output. Design and estimator for the total sensitivity index. 181(2):259–270.
