# Supplementary figures and images for: Probabilistic modeling of cell cycle dynamics in response to cell cycle targeting chemotherapy drugs to guide treatment strategies
Source: PLoS Comput Biol. 2025 Dec 16;21(12):e1013790. doi: 10.1371/journal.pcbi.1013790 (PMC12707676; doi:10.1371/journal.pcbi.1013790)

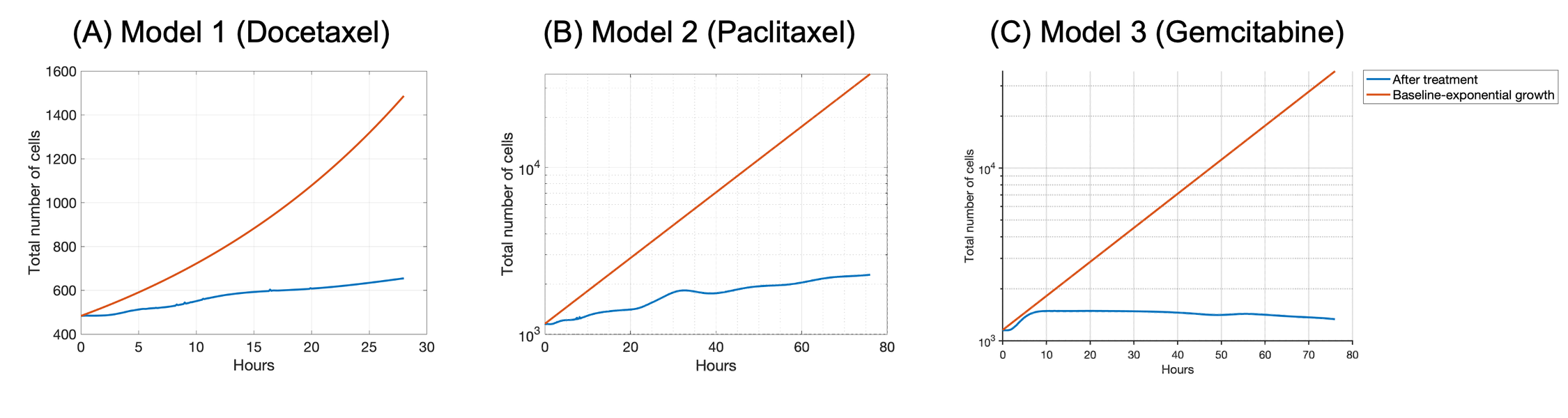

Supplement: S1 Fig — The plot shows the total number of cells following treatment with docetaxel, paclitaxel, or gemcitabine, at doses listed in Table 4 compared to baseline exponential-phase growth. The y-axis indicates the number of cells in each state after TSSD, as computed from a branching process initiated by a single G1 ancestor cell at simulation time 0. (TIF) [file pcbi.1013790.s003.tif]

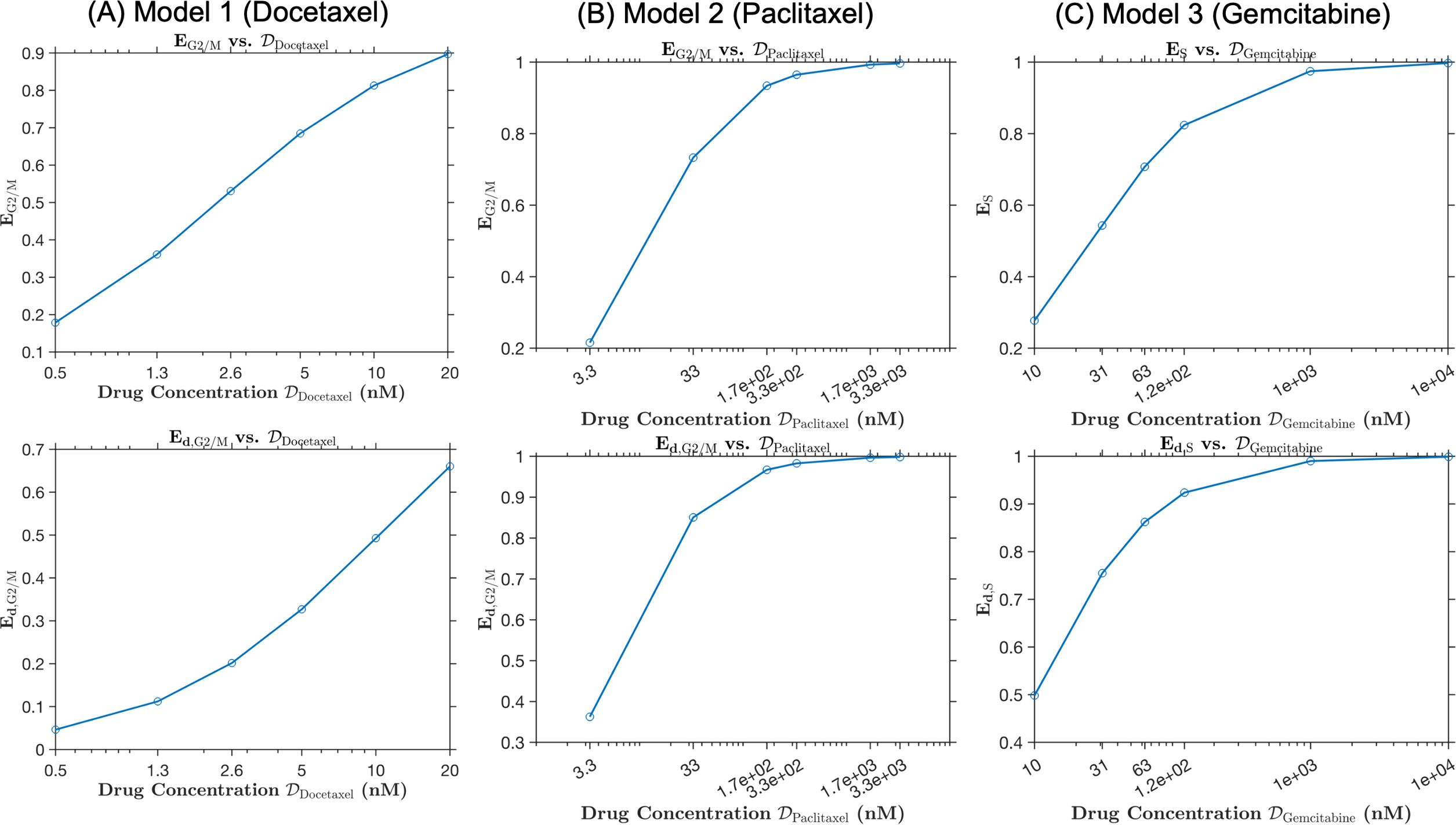

Supplement: S2 Fig — Panel (A) shows the values of EG2/M and Ed,G2/M for docetaxel at concentrations of 0.5, 1.3, 2.6, 5, 10, and 20 nM. Panel (B) shows the values of EG2/M and Ed,G2/M for paclitaxel at concentrations of 3.3, 33, 170, 330, 1700, and 3300 nM. Panel (C) shows the values of ES and Ed,S for gemcitabine at concentrations of 10, 31, 63, 122, 1000, and 10000 nM. (TIF) [file pcbi.1013790.s004.tif]
